# Synthesis of 2‑Aryl-3-(organoselanyl)‑4*H*‑benzo[4,5]imidazo[2,1‑*b*][1,3]thiazines Promoted by *N*‑Fluorobenzenesulfonimide (NFSI)

**DOI:** 10.1021/acs.joc.5c01598

**Published:** 2025-09-29

**Authors:** Ricardo H. Bartz, Pedro S. Souza, Leonardo Rocha, Raquel G. Jacob, Eder J. Lenardão, Márcio S. Silva, Gelson Perin

**Affiliations:** Laboratório de Síntese Orgânica Limpa - LASOL, CCQFA, Universidade Federal de Pelotas - UFPel, P.O. Box 354, 96010-900 Pelotas, RS, Brazil

## Abstract

An approach has been developed for the direct synthesis of various selenium-functionalized benzo­[4,5]­imidazo­[2,1-*b*]­[1,3]­thiazine derivatives via the electrophilic cyclization reaction between 2-(methylthio)-1-(3-arylprop-2-yn-1-yl)-1*H*-benzo­[*d*]­imidazoles and diorganyl diselenides promoted by *N*-fluorobenzenesulfonimide (NFSI). This strategy is used to construct fused heterocyclic scaffolds based on selenium-functionalized 4*H*-benzo­[4,5]­imidazo­[2,1-*b*]­[1,3]­thiazines using NFSI as a stable and nonhazardous oxidizing agent in the presence of CH_3_CN at 80 °C. The protocol allowed a broad substrate scope leading to the synthesis of 16 novel selenium-functionalized benzo­[4,5]­imidazo­[2,1-*b*]­[1,3]­thiazines in yields of up to 95%. Furthermore, control studies, including ^1^H, ^19^F, and ^77^Se NMR experiments, were conducted to identify intermediates, which contributed to the elucidation of the reaction mechanism.

## Graphical abstract

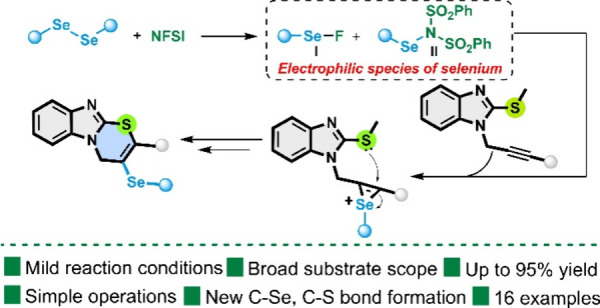

## Introduction

Benzimidazole-based compounds are privileged structures that possess remarkable biological activities.^1^ More specifically, benzo­[4,5]­imidazo­[2,1-*b*]­[1,3]­thiazine derivatives exhibit considerable pharmacological activities, including antituberculous^2^ and antithrombotic activities,^3^ and have potential effects as modulators of α-synuclein amyloid aggregation^4^ (Figure 1, compounds **A**–**C**, respectively). In addition, benzo­[4,5]­imidazo­[2,1-*b*]­[1,3]­thiazines have applications in materials science, as is the case for compound **D**, which has been shown to be a type of electron acceptor core in organic light-emitting diodes (OLEDs)^5^ (Figure 1). Considering the interesting properties presented by benzo­[4,5]­imidazo­[2,1-*b*]­[1,3]­thiazine derivatives, their synthesis became a significant source of inspiration for chemists.

**Figure 1:**
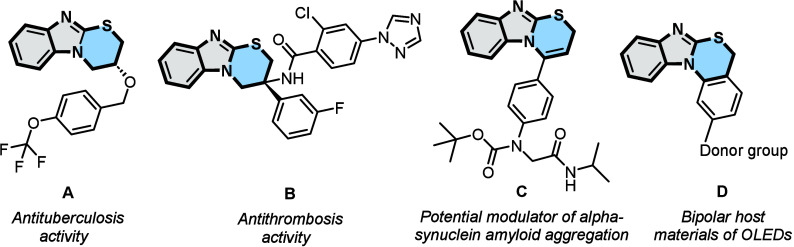
Potential applications of benzo­[4,5]­imidazo­[2,1-*b*]­[1,3]­thiazine derivatives.

Another class of substances with broad application prospects consists of organoselenium compounds, since they exhibit considerable pharmacological activities, including antioxidant,^6^ antiviral,^7^ and anticancer activities.^8^ In addition to their diverse medicinal applications,^9^ organoselenium compounds also serve as important nutrients in supplements, which protect the body against oxidative damage that can trigger neurodegenerative and cardiovascular diseases, cancer, diabetes, and viral infections.^10^ Therefore, given the broad application prospects of organoselenium compounds, the development of new bioactive molecules, such as benzo­[4,5]­imidazo­[2,1-*b*]­[1,3]­thiazine derivatives hybridized with an organoselenium moiety, is quite attractive from a medicinal point of view.

To the best of our knowledge, there is only one synthetic method that describes the synthesis of selenium-functionalized benzo­[4,5]­imidazo­[2,1-*b*]­[1,3]­thiazines, described by Sarkar and co-workers in 2021.^11^ This electrochemical procedure involves the cyclization reaction of thiopropargyl benzoimidazole with diorganoyl diselenides using an undivided cell equipped with a graphite anode and a platinum plate cathode. Thus, using CH_3_CN as the solvent and LiClO_4_ as the electrolyte at a constant current of 10 mA, only one example was obtained with 92% yield after reaction for 1 h (Scheme 1A).

**Scheme 1:**
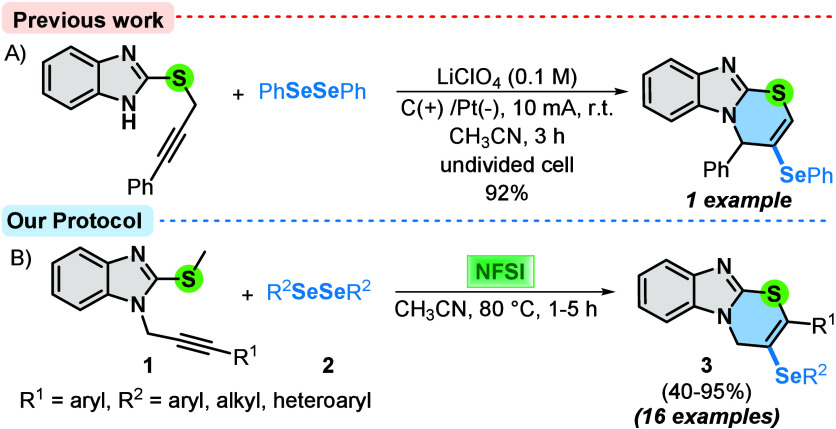
Synthesis of Selenium-Functionalized Benzo­[4,5]­imidazo­[2,1-*b*]­[1,3]­thiazines

The use of transition metals or oxidizing species such as I_2_, Oxone, trichloroisocyanuric acid (TCCA), and SelectFluor to generate electrophilic selenium species *in situ* is currently one of the main strategies used to synthesize selenium-functionalized organic molecules.^12^ On the other hand, the search for sustainable reagents for the insertion of organoselenium compounds into organic molecules is a topic of great interest. In this sense, *N*-fluorobenzenesulfonimide (NFSI) has been explored as an oxidizing agent along with diselenides in several transformations.^13^ NFSI is easy to handle, stable, and cheap, providing the desirable characteristics for an oxidant.^14^ However, to the best of our knowledge, the use of NFSI and diselenides in cyclization reactions has not been reported, which makes this an interesting research topic.

As part of our interest in the synthesis of selenium-decorated heterocycles,^15^ we describe here a versatile and efficient approach for obtaining several selenium-functionalized benzo­[4,5]­imidazo­[2,1-*b*]­[1,3]­thiazine derivatives **3** (Scheme 1B). The protocol proceeds through a cyclization reaction of 2-(methylthio)-1-(3-arylprop-2-yn-1-yl)-1*H*-benzo­[*d*]­imidazoles **1** with diorganoyl diselenides **2** using NFSI as the oxidant and acetonitrile as the solvent at 80 °C. With this strategy, two new C–Se and C–S bonds are simultaneously formed, offering a practical approach for the building of fused heterocyclic compounds.

## Results and Discussion

At the beginning, starting materials **1a**–**h** were synthesized using protocols already described, with minor modifications.^16^ Thus, initially 2-(methylthio)-1*H*-benzo­[*d*]­imidazole **5** was previously synthesized from 1*H*-benzo­[*d*]­imidazole-2-thiol **4**. Substrate **5** was then reacted with propargyl bromide to generate terminal alkyne **6**, followed by the Sonogashira cross-coupling reaction with aryl iodide, producing 2-(methylthio)-1-(3-arylprop-2-yn-1-yl)-1*H*-benzo­[*d*]­imidazole derivatives **1a**–**h** (Scheme 2). The characterization data of **1a**–**h** starting materials along with the NMR spectra are included in the Supporting Information.

**Scheme 2:**
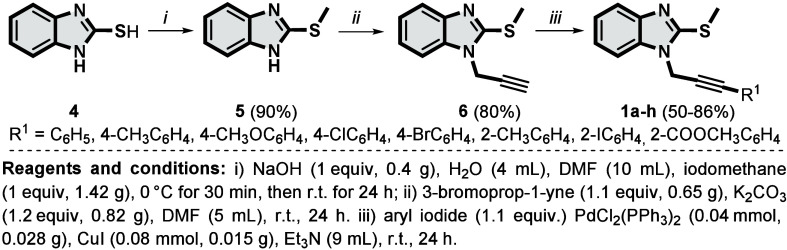
Synthesis of Starting Materials **1a–h**

Based on our previous cyclization protocol to access selenium-functionalized heterocyclic compounds,^15a^ we started our optimization study by stirring a mixture of 2-(methylthio)-1-(3-phenylprop-2-yn-1-yl)-1*H*-benzo­[*d*]­imidazole **1a** (0.15 mmol), diphenyl diselenide **2a** (0.075 mmol), and Oxone (0.15 mmol) in CH_3_CN (2 mL) at 80 °C. As shown in Table 1, the desired product **3a** was obtained in only 40% yield after 2 h. The low yield of product **3a** is associated with the formation of sulfoxide **7** (51% yield), obtained after oxidation of **1a** by Oxone. Then, a study was performed with different oxidants such as potassium persulfate (K_2_S_2_O_8_), ammonium persulfate [(NH_4_)_2_S_2_O_8_], *tert*-butyl hydroperoxide (TBHP), *N*-fluorobenzenesulfonimide (NFSI), and SelectFluor (Table 1, entries 2–6, respectively) in an attempt to increase the amount of product **3a**. As one can see in Table 1, only fluorine-based oxidants led to the formation of product **3a** in excellent yields. Furthermore, when compared to the use of Oxone, NFSI performed much better, providing **3a** in 95% yield after 1 h at 80 °C (Table 1, entry 5 vs entry 1). On the other hand, the use of SelectFluor also generated product **3a** in a good yield (85% after 1 h). Considering the lower cost and better performance, NFSI was defined as the best oxidant for this reaction.

**Table 1:**
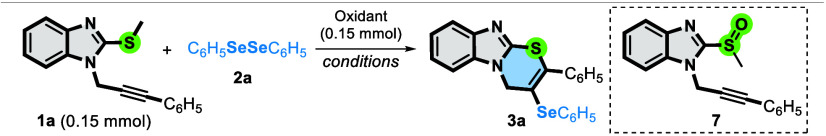
Optimization of the Reaction Conditions^a^

	oxidant	**2a** (mmol)	solvent	temp (°C)	time (h)	yield of **3a** (%)^b^
1	Oxone	0.075	CH_3_CN	80	2	40^c^
2	K_2_S_2_O_8_	0.075	CH_3_CN	80	24	NR^a^
3	(NH_4_)_2_S_2_O_8_	0.075	CH_3_CN	80	24	NR^a^
4	TBHP	0.075	CH_3_CN	80	24	NR^a^
5	NFSI	0.075	CH_3_CN	80	1	95
6	SelectFluor	0.075	CH_3_CN	80	1	85
7	NFSI	0.075	DMF	80	24	50
8	NFSI	0.075	CH_3_NO_2_	80	24	25
9	NFSI	0.075	EtOH	80	24	20
10	NFSI	0.075	MeOH	80	24	15
11	NFSI	0.075	PEG-400	80	24	23
12	NFSI	0.075	CH_3_CN	25	24	48
13	NFSI	0.075	CH_3_CN	50	6	71
14	NFSI	0.090	CH_3_CN	80	1	97
15^d^	NFSI	0.075	CH_3_CN	80	2	75
16^e^	NFSI	0.075	CH_3_CN	80	1	70

a For the reactions, a mixture of 2-(methylthio)-1-(3-phenylprop-2-yn-1-yl)-1*H*-benzo­[*d*]­imidazole **1a** (0.15 mmol), an oxidant (0.15 mmol), and diphenyl diselenide **2a** in a solvent (2.0 mL) was stirred at the temperature for the time indicated.

b Isolated yields.

c Sulfoxide **7** was formed in 51% yield.

d With 0.110 mmol of NFSI.

e With 0.180 mmol of NFSI.

f No reaction.

As a continuation of the optimization tests, a study of different polar protic and aprotic solvents was performed (Table 1, entries 7–11). The use of the polar aprotic solvents DMF and CH_3_NO_2_ led to the formation of product **3a** after reaction for 24 h in 50% and 25% yields, respectively (Table 1, entries 7 and 8, respectively). Similar results were observed when EtOH, MeOH, and PEG-400 were used, and **3a** was obtained in lower yields of 20%, 15%, and 23%, respectively (Table 1, entries 9–11, respectively). Thus, acetonitrile was defined as the best solvent for this cyclization.

Then, two tests were performed by decreasing the reaction temperature to 25 and 50 °C. In both cases, the reaction time increased while the reaction efficiency decreased, leading to the formation of **3a** in 48% and 71% yields after 24 and 6 h, respectively (Table 1, entries 12 and 13, respectively).

In the next step, the stoichiometry of the reaction was adjusted by using 0.090 mmol of diphenyl diselenide **2a**. Thus, after reaction for 1 h at 80 °C, product **3a** was obtained in 97% yield (Table 1, entry 14). Since the yield and reaction time remained practically the same as those observed when 0.075 mmol of **2a** was used, this was defined as the ideal amount.

Finally, the effect of the amount of NFSI was evaluated. It was observed that using 0.110 and 0.180 mmol of NFSI caused a decrease in the yields of product **3a**, 75% (after 2 h) and 70% (after 1 h), respectively (Table 1, entries 15 and 16, respectively). Given these tests, the best reaction condition for obtaining product **3a** is represented in entry 5 (Table 1). Additionally, the reaction could be scaled up to 3 mmol, producing product **3a** in 70% yield (882 mg) (Table 2).

**Table 2:**
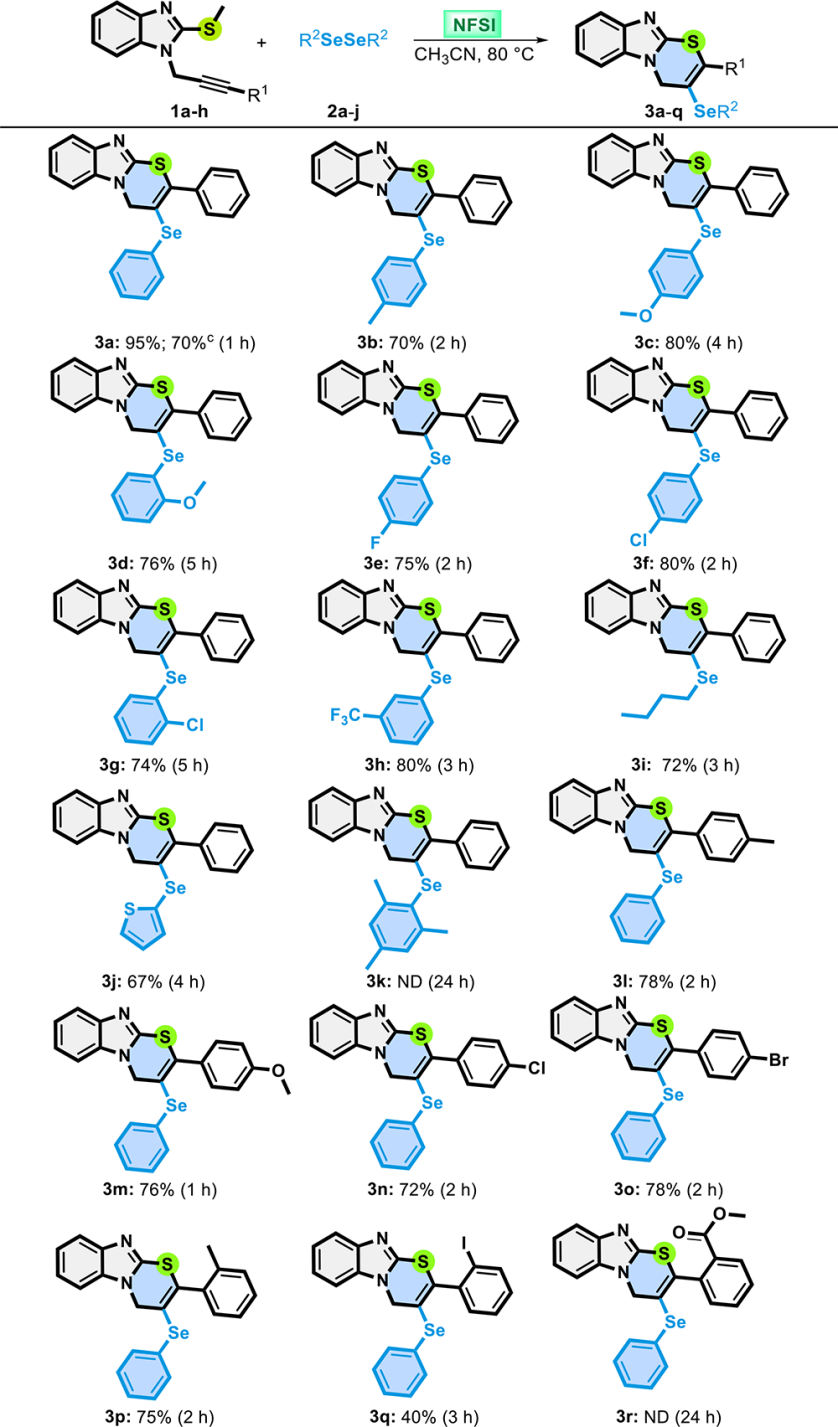
Substrate Scope of the Synthesis of Compounds **3a**–**q** ^a^,^b^

a For the reactions, a mixture of **1a**–**h** (0.15 mmol), NFSI (0.15 mmol), and diorganyl diselenides **2a**–**k** (0.075 mmol) in CH_3_CN as the solvent (2.0 mL) was stirred at 80 °C for the time indicated. ND = not detected.

b Isolated yields.

c On a 3 mmol scale.

With the best conditions in hand, a variety of alkynyl benzoimidazoles **1** and diorganyl diselenides **2** were employed to explore the generality of the protocol (Table 2). First, substrate **1a** reacted with different electron-rich and -poor diaryl diselenides, as well as dibutyl and dithienyl diselenides **2**. We found that the presence of electron-donating substituents -CH_3_ and -OCH_3_ at the *para* position of the aromatic ring of diselenides **2b** and **2c**, respectively, caused a slight decrease in the reaction yield, and products **3b** and **3c** were obtained in 70% and 80% yields after reaction for 2 and 4 h, respectively (Table 2). When *o*-methoxy-substituted diselenide **2d** was used, product **3d** was obtained in 76% yield after reaction for 5 h. This new approach tolerates halogen substituents (4-F, 4-Cl, and 3-Cl) in aryl diselenides **2e**–**g**, and products **3e**–**g**, respectively, were obtained in good yields, ranging from 74% to 80%. Satisfactorily, diselenide **2h**, containing the strong electron-withdrawing 3-CF_3_ group, afforded the desired product **3h** in 80% yield after reaction for 3 h (Table 2).

Additionally, substrate **1a** reacted with dibutyl diselenide **2i**, affording product **3i** in 72% yield after 3 h (Table 2). Heteroaromatic diselenide **2j** (R^2^ = 2-thienyl) was also a suitable substrate, giving after rection with **1a** for 4 h product **3j** in 67% yield. In addition to these results, the steric effect caused by dimesityl diselenide **2k** negatively affects the reaction performance, and product **3k** was not detected, even after reaction for 24 h (Table 2).

Furthermore, different alkynyl benzoimidazoles **1b**–**h** were evaluated to explore the versatility of this reaction protocol (Table 2). *para*-substituted alkynyl benzoimidazoles **1b** (R^1^ = 4-CH_3_C_6_H_4_), **1c** (R^1^ = 4-CH_3_OC_6_H_4_), **1d** (R^1^ = 4-ClC_6_H_4_), and **1e** (R^1^ = 4-BrC_6_H_4_) showed similar reactivities in the reaction with diphenyl diselenide **2a**, and products **3l**–**o** were obtained in 78%, 76%, 72%, and 78% yields, respectively (Table 2). In the sequence, *ortho*-substituted substrates **1f** (R^1^ = 2-CH_3_C_6_H_4_), **1g** (R^1^ = 2-IC_6_H_4_), and **1h** (R^1^ = 2-COOCH_3_C_6_H_4_) reacted with diphenyl diselenide **2a**. To our delight, products **3p** and **3q** could be obtained in satisfactory yields of 75% and 40%, respectively (Table 2). The synthesis of **3q** opens the possibility of further modifications in the molecule through cross-coupling reactions at the iodo-substituted ring. On the other hand, when *ortho*-substituted substrate **1h** (R^1^ = 2-COOCH_3_C_6_H_4_) was reacted with diphenyl diselenide **2a**, no product **3r** was formed, even after reaction for 24 h (Table 2).

Next, to gain a deeper understanding of the reaction mechanism, some control experiments were performed (Scheme 3). Initially, a reaction in the absence of NFSI was carried out (Scheme 3A). No reaction was observed, indicating that NFSI plays a vital role in the reaction by acting as an oxidant agent to generate the reactive species once substrates **1a** and **2a** are recovered at the end of the reaction. Next, substrate **1a** was reacted with NFSI alone, in the absence of **2a**, under the standard conditions (Scheme 3B). After 1 h, cyclic product **3a′** was not formed, indicating that the presence of diselenide is mandatory for the formation of the thiazine ring. When 3 equiv of radical inhibitor TEMPO or BHT was added under the standard conditions, the reaction was not significantly affected, and product **3a** was obtained in 87% or 85% yield, respectively (Scheme 3C). These results indicate that the reaction did not follow a radical pathway.

**Scheme 3:**
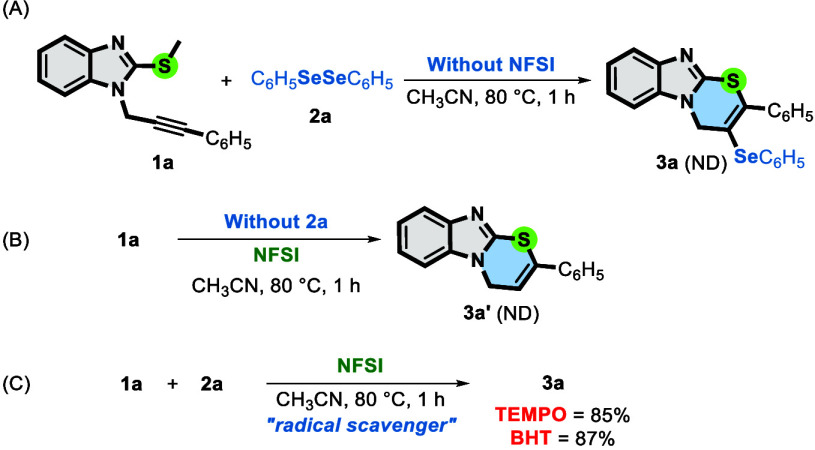
Control Experiments

To verify the formation of the electrophilic selenium species in the reaction medium, ^19^F and ^77^Se NMR analyses were carried out. First, to determine the chemical shift of the fluorine nucleus in NFSI, a ^19^F experiment was performed. Thus, 0.15 mmol of NFSI was solubilized in 1 mL of CD_3_CN and transferred to an NMR tube, and a ^19^F NMR experiment was performed. As shown in Figure 2A (green spectrum), a peak at δ = −39.8 ppm was observed.^14b^

**Figure 2:**
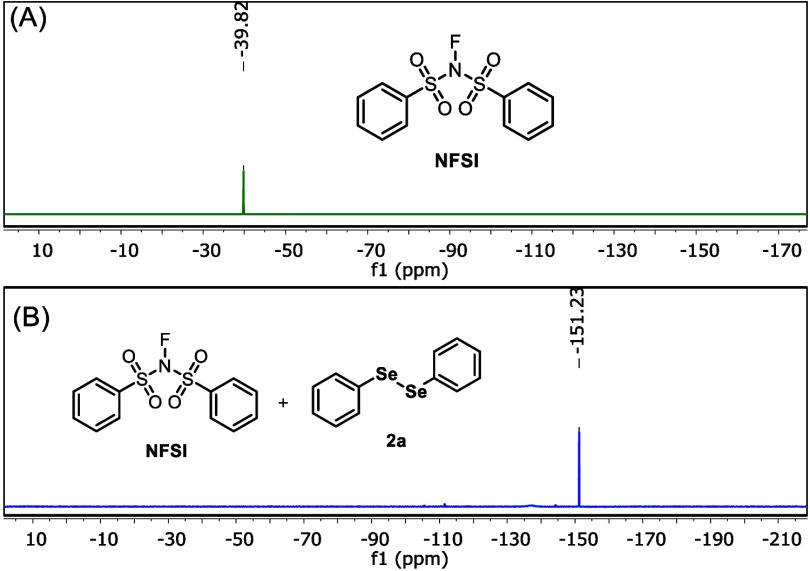
^19^F NMR spectra (376 MHz, CD_3_CN, 25 °C) of NFSI (green) and a mixture of NFSI and diphenyl diselenide **2a** (blue).

Next, an experiment was carried out by mixing 0.075 mmol of **2a** with 0.30 mmol of NFSI in 1 mL of CD_3_CN. The mixture was stirred for 10 min. Soon thereafter, 500 μL of the solution was transferred to an NMR tube, and a ^19^F NMR experiment was immediately performed. In this case, the disappearance of the NFSI signal and the appearance of a new peak at δ = −151.23 ppm were observed (Figure 2B, blue spectrum). This result suggests that a new fluorine species was formed. Additionally, a ^77^Se­{^1^H} NMR experiment was performed to verify the formation of the electrophilic selenium species. As shown in Figure 3, two signals were detected. The first signal at δ = 1383.00 ppm corresponds to PhSe-F^12e^ species **I**, formed by the reaction between NFSI and diphenyl diselenide **2a** (Figure 3). The signal at δ = 1071.31 ppm, instead, corresponds to PhSeN­(SO_2_Ph)_2_ species, formed from the other portion of NFSI (Figure 3). The ^77^Se NMR chemical shift of the Se bonded to N in **II** is consistent with the literature.^17^

**Figure 3:**
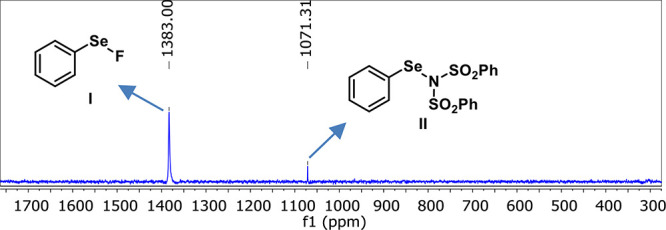
^77^Se­{^1^H} NMR spectrum (76.3 MHz, CD_3_CN, 25 °C) of a mixture of NFSI and **2a** (2:1 ratio).

Furthermore, a GC-MS analysis of the crude reaction mixture was performed in order to investigate other species formed in the reaction medium. In addition to the mass of product **3a**, the mass corresponding to *m*/*z* 311 g/mol (see Figure S74) was also observed. This mass corresponds to coproduct CH_3_N­(SO_2_Ph)_2_, formed by the reaction between anion [-N­(SO_2_Ph)_2_] and the methyl group bonded to the sulfur atom in the cyclization step. ^1^H NMR analysis of the reaction crude also confirmed the presence of a signal at δ = 3.18 ppm characteristic of the methyl group (see Figure S75). This signal is also in agreement with the literature data.^18^

Based on the experiments performed and according to the literature,^12e,13^ a plausible mechanism is proposed (Scheme 4). In the initial step, electrophilic selenium species **I** (^77^Se­{^1^H} δ = 1383.00 ppm;^12e^ ^19^F δ = −151.23 ppm) and **II** (^77^Se­{^1^H} δ = 1071.31 ppm) are formed from the reaction of diselenide **2** with NFSI. In the next step, intermediate **I** or **II** (R^2^Se-X) reacts with the triple bond of **1** to generate seleniranium **III**, which undergoes an intramolecular attack by the electron pair of the sulfur atom, generating intermediate **IV**. Finally, **IV** undergoes demethylation by a nucleophilic species [-N­(SO_2_Ph)_2_] present in the reaction medium, generating product **3**, together with *N*-methyl-*N*-(phenylsulfonyl)­benzenesulfonamide as a coproduct (Scheme 4).

**Scheme 4:**
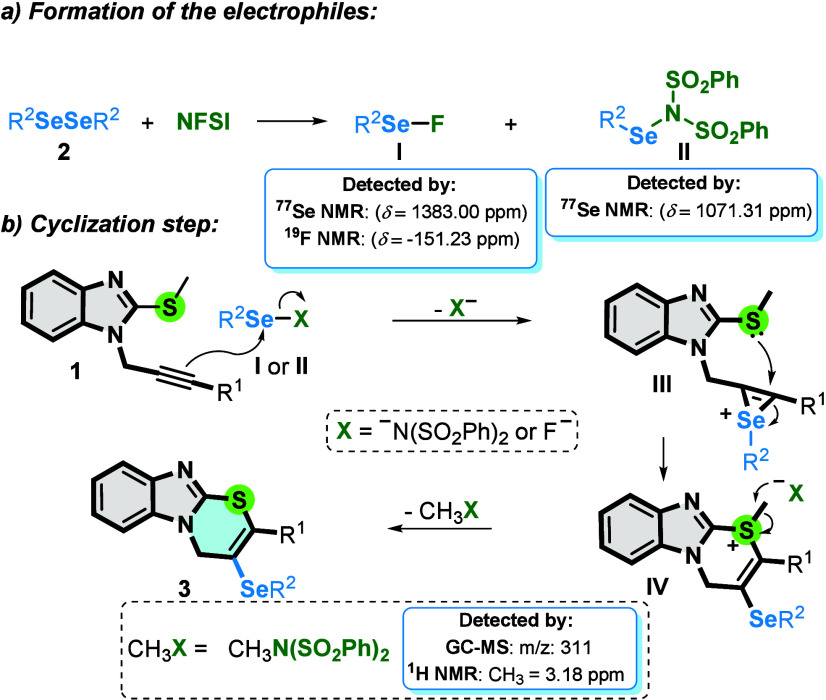
Plausible Reaction Mechanism

## Conclusions

In summary, we have developed an efficient strategy with good functional group tolerance for the synthesis of selenium-functionalized benzo­[4,5]­imidazo­[2,1-*b*]­[1,3]­thiazine derivatives via the electrophilic selenocyclization of 2-(methylthio)-1-(3-arylprop-2-yn-1-yl)-1*H*-benzo­[*d*]­imidazoles promoted by *N*-fluorobenzenesulfonimide (NFSI). Sixteen unprecedented compounds were prepared in good to excellent yields. Furthermore, control studies, including ^1^H, ^19^F, and ^77^Se NMR spectroscopy experiments, were conducted to identify intermediates, which contributed to the elucidation of the reaction mechanism.

## Experimental Section

### General Information

The reactions were monitored by TLC carried out on Merk silica gel (60 F_254_) using UV light as a visualization agent, and the mixture of 5% vanillin and 10% H_2_SO_4_ under heating conditions was used as a developing agent. Merck silica gel (particle size of 0.040–0.063 mm) was used for flash chromatography. Hydrogen nuclear magnetic resonance spectra (^1^H NMR) were obtained on a Bruker Avance III HD 400 MHz instrument employing a direct broadband probe at 400 MHz. The spectra were recorded in CDCl_3_ solutions. Chemical shifts are reported in parts per million, referenced to tetramethylsilane (TMS) as the internal reference. Coupling constants (*J*) are reported in hertz. Abbreviations to denote the multiplicity of a particular signal are s (singlet), d (doublet), dd (doublet of doublets), t (triplet), q (quartet), quint (quintet), sex (sextet), td (triplet of doublets), and m (multiplet). Carbon-13 nuclear magnetic resonance spectra (^13^C NMR) were obtained on a Bruker Avance III HD 400 MHz instrument employing a direct broadband probe at 100 MHz. The chemical shifts are reported in parts per million, referenced to the solvent peak of CDCl_3_. Selenium-77 nuclear magnetic resonance spectra (^77^Se NMR) were obtained on a Bruker Avance III HD 400 spectrometer at 76 MHz using CDCl_3_ as the deuterated solvent and the diphenyl diselenide (δ Se = 463 ppm) for the reference using the substitution method (IUPAC).^19^ Fluorine-19 nuclear magnetic resonance spectra (^19^F NMR) were obtained on a Bruker Avance III HD 400 MHz instrument employing a direct broadband probe. Spectra were recorded in CDCl_3_ as the deuterated solvent and α,α,α-trifluorotoluene (−63.72 ppm) for the reference using the substitution method (IUPAC).^19^ High-resolution mass spectra (HRMS) were performed in a HESI Quadrupole-Orbitrap (Q extractive focus, Thermo Scientific) spectrometer equipped with an APCI source operating in positive mode. The samples were solubilized in acetonitrile and analyzed by direct infusion at a constant flow rate. The acquisition parameters were as follows: scan type, full MS; resolution, 70 000; polarity, positive. Ionization conditions for HESI were as follows: sheath gas, 20; auxiliary gas, 10; spray voltage, 2.8 kV; capillary temperature, 300 °C. The mass-to-charge ratio (*m*/*z*) data were processed and analyzed using Bruker Daltonics software: Compass Data Analysis and the Isotope Pattern. For compounds **1c**, **1e**, **1h**, **3m**, and **3o**, high-resolution mass spectra (HRMS) were recorded in positive ion mode (APCI) using a Q-TOF spectrometer. Low-resolution mass spectra were obtained with a Shimadzu GC-MS-QP2010 mass spectrometer. Melting point values were measured in a Marte PFD III instrument.

### Procedure for the Synthesis of 2-(Methylthio)-1*H*-benzo­[*d*]­imidazole (**5**)

Compound **5** was prepared according to a published procedure with minor modifications.^16a^ In a 50 mL flask equipped with a magnetic stirring bar, 2-mercaptobenzimidazole **4** (10 mmol, 1 equiv, 1.5 g) and dimethylformamide (DMF, 10 mL, 1 M) were added. The mixture was stirred at 0 °C. Then, 4 mL of a 2.5 M aqueous solution of NaOH (1 equiv, 0.4 g) was added, and the reaction mixture was stirred at 0 °C for 30 min. Then, iodomethane (10 mmol, 1 equiv, 1.42 g) was added, and the reaction mixture was stirred for 24 h at room temperature. After the reaction was completed, 50 mL of cold water was added, generating a white precipitate. The precipitate was isolated by filtration, dried, and used in the next step without purification. Yield: 90%.

### Procedure for the Synthesis of 2-(Methylthio)-1-(prop-2-yn-1-yl)-1*H*-benzo­[*d*]­imidazole (**6**)

Compound **6** was prepared according to a published procedure, with minor modifications.^16b^ In a 50 mL single-neck round-bottom flask equipped with a magnetic stir bar, compound **5** (5.0 mmol, 1 equiv, 0.82 g), DMF (5 mL, 1 M), and K_2_CO_3_ (6 mmol, 1.2 equiv, 0.83 g) were added. The reaction mixture was stirred for 30 min at room temperature. Then, propargyl bromide (5.5 mmol, 1.1 equiv, 0.65 g) was added, and the reaction mixture was stirred at room temperature for 24 h. The progress of the reaction was monitored by TLC. Water was then added to the reaction mixture (10.0 mL), and the product was extracted with ethyl acetate (3 × 10.0 mL). The organic layer was separated, dried over MgSO_4_, filtered, and concentrated under vacuum. The crude residue was used in the next step without further purification.

### Procedure for the Synthesis of 2-(Methylthio)-1-(3-arylprop-2-yn-1-yl)-1*H*-benzo­[*d*]­imidazole Derivatives (**1a**–**h**)

In a 50.0 mL two-neck round-bottom flask under a nitrogen atmosphere, Pd­(PPh_3_)_2_Cl_2_ (0.04 mmol, 0.028 g) and 5 mL of triethylamine were added. Then, the appropriate amount of iodobenzene (2.2 mmol, 1.1 equiv) solubilized in triethylamine (2.0 mL, 1.1 M) was added. In the next step, a solution of compound **6** (2 mmol, 1 equiv, 0.40 g) in triethylamine (2.0 mL, 1 M) was added. The reaction mixture was stirred at room temperature (25 °C) for 5 min. Then, CuI (0.08 mmol, 0.015 g) was added, and stirring was continued for another 24 h. The resulting solution was taken up in a saturated aqueous NH_4_Cl solution (15.0 mL), and the product was extracted with ethyl acetate (3 × 10.0 mL). The organic layer was separated, dried over MgSO_4_, and concentrated in vacuo. The residue was purified by column chromatography using silica gel and a hexane/ethyl acetate eluent to furnish the desired products **1a**–**h** in 50–86% yields.

#### 2-(Methylthio)-1-(3-phenylprop-2-yn-1-yl)-1*H*-benzo­[*d*]­imidazole (**1a**)

Purified by column chromatography (85:15 hexane/ethyl acetate). Yield: 0.473 g (85%). Yellow solid. Mp: 75–78 °C. ^1^H NMR (CDCl_3_, 400 MHz): δ 7.72–7.68 (m, 1H), 7.46–7.42 (m, 1H), 7.40–7.37 (m, 2H), 7.31–7.21 (m, 5H), 5.05 (s, 2H), 2.81 (s, 3H). ^13^C­{^1^H} NMR (CDCl_3_, 100 MHz): δ 152.6, 143.7, 136.0, 132.0, 128.9, 128.5, 122.3, 122.1, 118.5, 109.2, 85.4, 81.8, 34.4, 15.2. MS (relative intensity (%)): *m*/*z* 278 (59.3), 263 (25.1), 163 (25.3), 115 (100.0), 77 (5.9). HRMS (APCI-QTOF): *m*/*z* [M + H]^+^ calcd for C_17_H_15_N_2_S, 279.0956; found, 279.0948.

#### 2-(Methylthio)-1-(3-(4-tolyl)­prop-2-yn-1-yl)-1*H*-benzo­[*d*]­imidazole (**1b**)

Purified by column chromatography (85:15 hexane/ethyl acetate). Yield: 0.467 g (80%). Orange oil. ^1^H NMR (CDCl_3_, 400 MHz): δ 7.72–7.68 (m, 1H), 7.47–7.42 (m, 1H), 7.28 (d, *J* = 8.1 Hz, 2H), 7.25–7.21 (m, 2H), 7.07 (d, *J* = 8.1 Hz, 2H), 5.05 (s, 2H), 2.81 (s, 3H), 2.31 (s, 3H). ^13^C­{^1^H} NMR (CDCl_3_, 100 MHz): δ 152.6, 143.7, 139.1, 136.1, 131.9, 129.2, 122.3, 119.0, 118.5, 109.3, 85.6, 81.1, 34.5, 21.6, 15.3. MS (relative intensity (%)): *m*/*z* 292 (46.1), 277 (23.0), 163 (4.6), 129 (100.0), 77 (10.1). HRMS (APCI-QTOF): *m*/*z* [M + H]^+^ calcd for C_18_H_17_N_2_S, 293.1112; found, 293.1104.

#### 1-(3-(4-Methoxyphenyl)­prop-2-yn-1-yl)-2-(methylthio)-1*H*-benzo­[*d*]­imidazole (**1c**)

Purified by column chromatography (85:15 hexane/ethyl acetate). Yield: 0.530 g (86%). Orange oil. ^1^H NMR (CDCl_3_, 400 MHz): δ 7.71–7.67 (m, 1H), 7.46–7.42 (m, 1H), 7.32 (d, *J* = 8.8 Hz, 2H), 7.25–7.21 (m, 2H), 6.78 (d, *J* = 8.8 Hz, 2H), 5.04 (s, 2H), 3.76 (s, 3H), 2.81 (s, 3H). ^13^C­{^1^H} NMR (CDCl_3_, 100 MHz): δ 160.1, 152.6, 143.7, 136.1, 133.4, 122.2, 118.4, 114.1, 114.09, 109.3, 85.4, 80.5, 55.4, 34.5, 15.2. MS (relative intensity (%)): *m*/*z* 308 (22.0), 293 (10.5), 163 (22), 145 (100.0), 77 (2.3). HRMS (APCI-QTOF): *m*/*z* [M + H]^+^ calcd for C_18_H_17_N_2_OS, 309.1062; found, 309.1063.

#### 1-(3-(4-Chlorophenyl)­prop-2-yn-1-yl)-2-(methylthio)-1*H*-benzo­[*d*]­imidazole (**1d**)

Purified by column chromatography (85:15 hexane/ethyl acetate). Yield: 0.518 g (83%). Orange solid. Mp: 63–65 °C. ^1^H NMR (CDCl_3_, 400 MHz): δ 7.72–7.68 (m, 1H), 7.42–7.39 (m, 1H), 7.30 (d, *J* = 8.6 Hz, 2H), 7.26–7.22 (m, 4H), 5.04 (s, 2H), 2.82 (s, 3H). ^13^C­{^1^H} NMR (CDCl_3_, 100 MHz): δ 152.5, 143.6, 135.9, 135.1, 133.2, 128.8, 122.4, 120.5, 118.5, 109.1, 84.3, 82.8, 34.3, 15.2. MS (relative intensity (%)): *m*/*z* 312 (56.9), 279 (14.4), 163 (27.7), 149 (100.0), 77 (7.7). HRMS (APCI-QTOF): *m*/*z* [M + H]^+^ calcd for C_17_H_14_ClN_2_S, 313.0566; found, 313.0556.

#### 1-(3-(4-Bromophenyl)­prop-2-yn-1-yl)-2-(methylthio)-1*H*-benzo­[*d*]­imidazole (**1e**)

Purified by column chromatography (85:15 hexane/ethyl acetate). Yield: 0.577 g (81%). Yellow solid. Mp: 87–90 °C. ^1^H NMR (CDCl_3_, 400 MHz): δ 7.72–7.69 (m, 1H), 7.43–7.39 (m, 3H), 7.25–7.22 (m, 4H), 5.04 (s, 2H), 2.82 (s, 3H). ^13^C­{^1^H} NMR (CDCl_3_, 100 MHz): δ 152.6, 143.7, 136.0, 133.4, 131.8, 123.3, 122.4, 121.0, 118.6, 109.1, 84.4, 83.0, 34.3, 15.2. MS (relative intensity (%)): *m*/*z* 356 (75.1), 277 (4.6), 163 (54.7), 114 (100.0), 77 (14.8). HRMS (APCI-QTOF): *m*/*z* [M + H]^+^ calcd for C_17_H_14_BrN_2_S, 357.0061; found, 357.0024.

#### 2-(Methylthio)-1-(3-(2-tolyl)­prop-2-yn-1-yl)-1*H*-benzo­[*d*]­imidazole (**1f**)

Purified by column chromatography (85:15 hexane/ethyl acetate). Yield: 0.444 g (76%). Yellow oil. ^1^H NMR (CDCl_3_, 400 MHz): δ 7.65–7.61 (m, 1H), 7.41–7.36 (m, 1H), 7.29–7.27 (m, 1H), 7.19–7.15 (m, 2H), 7.12 (dd, *J* = 7.3 and 1.3 Hz, 1H), 7.08–7.06 (m, 1H), 7.04–7.00 (m, 1H), 5.04 (s, 2H), 2.75 (s, 3H), 2.26 (s, 3H). ^13^C­{^1^H} NMR (CDCl_3_, 100 MHz): δ 152.6, 143.7, 140.7, 136.0, 132.3, 129.6, 129.0, 125.7, 122.35, 122.3, 121.9, 118.5, 109.3, 85.6, 84.4, 34.5, 20.8, 15.2. MS (relative intensity (%)): *m*/*z* 292 (52.5), 277 (23.8), 163 (8.2), 128 (100.0), 77 (13.9). HRMS (APCI-QTOF): *m*/*z* [M + H]^+^ calcd for C_18_H_17_N_2_S, 293.1112; found, 293.1103.

#### 1-(3-(2-Iodophenyl)­prop-2-yn-1-yl)-2-(methylthio)-1*H*-benzo­[*d*]­imidazole (**1g**)

Purified by column chromatography (85:15 hexane/ethyl acetate). Yield: 0.404 g (50%). Yellow oil. ^1^H NMR (CDCl_3_, 400 MHz): δ 7.72 (dd, *J* = 8.0 and 0.8 Hz, 1H), 7.63–7.61 (m, 1H), 7.45–7.43 (m, 1H), 7.30 (dd, *J* = 7.8 and 1.6 Hz, 1H), 7.19–7.15 (m, 3H), 6.91 (dd, *J* = 7.8 and 1.6 Hz, 1H), 5.06 (s, 2H), 2.75 (s, 3H). ^13^C­{^1^H} NMR (CDCl_3_, 100 MHz): δ 152.6, 143.8, 138.9, 133.2, 130.1, 128.7, 127.9, 122.42, 122.4, 118.5, 109.5, 87.2, 85.7, 34.5, 15.3. MS (relative intensity (%)): *m*/*z* 404 (90.2), 277 (52.5), 163 (17.9), 114 (100.0), 77 (12.5). HRMS (APCI-QTOF): *m*/*z* [M + H]^+^ calcd for C_17_H_14_IN_2_S, 404.9922; found, 404.9914.

#### Methyl 2-(3-(2-(Methylthio)-1*H*-benzo­[*d*]­imidazol-1-yl)­prop-1-yn-1-yl)­benzoate (**1h**)

Purified by column chromatography (85:15 hexane/ethyl acetate). Yield: 0.471 g (70%). White solid. Mp: 72–75 °C. ^1^H NMR (CDCl_3_, 400 MHz): δ 7.86 (d, *J* = 7.7 and 1.2 Hz, 1H), 7.72–7.70 (m, 1H), 7.47–7.43 (m, 2H), 7.36 (td, *J* = 7.5 and 1.4 Hz, 1H), 7.30 (td, *J* = 7.5 and 1.4 Hz, 1H), 7.23–7.21 (m, 2H), 5.09 (s, 2H), 3.65 (s, 3H), 2.80 (s, 3H). ^13^C­{^1^H} NMR (CDCl_3_, 100 MHz): δ 166.4, 152.3, 143.5, 135.8, 134.3, 132.1, 131.6, 130.4, 128.4, 122.2, 122.1, 118.2, 109.2, 86.6, 83.7, 51.9, 34.4, 15.0. MS (relative intensity (%)): *m*/*z* 336 (43.5), 321 (100.0), 277 (12.1), 163 (9.0), 143 (65.2), 77 (8.9). HRMS (APCI-QTOF): *m*/*z* [M + H]^+^ calcd for C_19_H_17_N_2_O_2_S, 337.1011; found, 337.1013.

### Procedure for the Synthesis of 2-Aryl-3-(organoselanyl)-4*H*-benzo­[4,5]­imidazo­[2,1-*b*]­[1,3]­thiazines (**3a**–**q**)

To a 10 mL tube equipped with a magnetic stirrer were added the appropriate 2-(methylthio)-1-(3-arylprop-2-yn-1-yl)-1*H*-benzo­[*d*]­imidazole **1a**–**h** (0.150 mmol, 1 equiv), diorganyl diselenide **2a**–**h** (0.075 mmol, 1 equiv), NFSI (0.150 mmol, 0.047 g, 1 equiv), and CH_3_CN (2.0 mL, 0.037 M). Next, the reaction mixture was stirred (magnetic stirring) and heated to 80 °C (oil bath) for the time indicated in Scheme 3. The reaction was monitored by TLC to evaluate the consumption of the starting materials. After completion of the reaction, the solvent was evaporated under reduced pressure, and the crude product was subsequently purified by column chromatography using a hexane/ethyl acetate eluent.

### Procedure for the Synthesis of **3a** for Scaling Up to 3 mmol

To a 50 mL flask equipped with a magnetic stirrer were added 2-(methylthio)-1-(3-phenylprop-2-yn-1-yl)-1*H*-benzo­[*d*]­imidazole **1a** (3 mmol, 0.834 g, 1 equiv), diphenyl diselenide **2a** (1.5 mmol, 0.471 g, 1 equiv), NFSI (3 mmol, 0.940 g, 1 equiv), and CH_3_CN (10.0 mL, 0.15 M). Next, the reaction mixture was stirred (magnetic stirring) and heated to 80 °C (oil bath) for 1 h. The reaction was monitored by TLC to evaluate the consumption of starting materials. After completion of the reaction, the solvent was evaporated under reduced pressure, and the crude product was subsequently purified by column chromatography using an 85:15 hexane/ethyl acetate eluent to afford 0.882 g of pure **3a** (70%).

#### 2-Phenyl-3-(phenylselanyl)-4*H*-benzo­[4,5]­imidazo­[2,1-*b*]­[1,3]­thiazine (**3a**)

Purified by column chromatography (85:15 hexane/ethyl acetate). Yield: 0.060 g (95%). White solid. Mp: 132–135 °C. ^1^H NMR (CDCl_3_, 400 MHz): δ 7.68 (d, *J* = 8.6 Hz, 1H), 7.45–7.42 (m, 7H), 7.31–7.23 (m, 4H), 7.19–7.15 (m, 1H), 6.97 (d, *J* = 8.0 Hz, 1H), 4.90 (s, 2H). ^13^C­{^1^H} NMR (CDCl_3_, 100 MHz): δ 145.6, 143.7, 137.4, 134.0, 133.04, 133.0, 129.9, 129.8, 129.4, 128.9, 128.86, 128.4, 123.0, 122.4, 118.8, 113.2, 108.6, 49.0. ^77^Se­{^1^H} NMR (CDCl_3_, 76 MHz): δ 411.1. MS (relative intensity (%)): *m*/*z* 420 (49.7), 339 (23.0), 263 (100.0), 115 (28.4), 77 (26.8). HRMS (APCI-QTOF): *m*/*z* [M + H]^+^ calcd for C_22_H_17_N_2_SSe, 421.0278; found, 421.0266.

#### 2-Phenyl-3-(4-tolylselanyl)-4*H*-benzo­[4,5]­imidazo­[2,1-*b*]­[1,3]­thiazine (**3b**)

Purified by column chromatography (85:15 hexane/ethyl acetate). Yield: 0.046 g (70%). Yellow solid. Mp: 159–161 °C. ^1^H NMR (CDCl_3_, 400 MHz): δ 7.67 (d, *J* = 7.9 Hz, 1H), 7.46–7.43 (m, 5H), 7.34 (d, *J* = 8.0 Hz, 2H), 7.26–7.22 (m, 1H), 7.19–7.15 (m, 1H), 7.09 (d, *J* = 8.0 Hz, 2H), 6.96 (d, *J* = 7.9 Hz, 1H), 4.86 (s, 2H), 2.33 (s, 3H). ^13^C­{^1^H} NMR (CDCl_3_, 100 MHz): δ 145.7, 143.7, 138.7, 137.4, 134.0, 133.7, 131.6, 130.7, 129.8, 129.5, 128.8, 124.8, 122.9, 122.3, 118.8, 113.6, 108.6, 48.9, 21.4. ^77^Se­{^1^H} NMR (CDCl_3_, 76 MHz): δ 406.7. MS (relative intensity (%)): *m*/*z* 434 (56.5), 339 (6.2), 263 (100.0), 115 (36.7), 77 (17.9). HRMS (APCI-QTOF): *m*/*z* [M + H]^+^ calcd for C_23_H_19_N_2_SSe, 435.0434; found, 435.0423.

#### 3-((4-Methoxyphenyl)­selanyl)-2-phenyl-4*H*-benzo­[4,5]­imidazo­[2,1-*b*]­[1,3]­thiazine (**3c**)

Purified by column chromatography (85:15 hexane/ethyl acetate). Yield: 0.054 g (80%). Yellow solid. Mp: 138–141 °C. ^1^H NMR (CDCl_3_, 400 MHz): δ 7.66 (d, *J* = 8.0 Hz, 1H), 7.47–7.45 (m, 4H), 7.42 (d, *J* = 8.6 Hz, 2H), 7.26–7.15 (m, 3H), 6.96 (d, *J* = 7.9 Hz, 1H), 6.83 (d, *J* = 8.6 Hz, 2H), 4.82 (s, 2H), 3.80 (s, 3H). ^13^C­{^1^H} NMR (CDCl_3_, 100 MHz): δ 160.5, 145.9, 143.7, 137.4, 136.4, 134.0, 130.0, 129.8, 129.5, 128.9, 123.0, 122.3, 118.8, 118.0, 115.5, 114.4, 108.6, 55.6, 48.6. ^77^Se­{^1^H} NMR (CDCl_3_, 76 MHz): δ 405.4. MS (relative intensity (%)): *m*/*z* 450 (56.5), 263 (100.0), 115 (74.2), 77 (25.8). HRMS (APCI-QTOF): *m*/*z* [M + H]^+^ calcd for C_23_H_19_N_2_OSSe, 451.0383; found, 451.0374.

#### 3-((2-Methoxyphenyl)­selanyl)-2-phenyl-4*H*-benzo­[4,5]­imidazo­[2,1-*b*]­[1,3]­thiazine (**3d**)

Purified by column chromatography (85:15 hexane/ethyl acetate). Yield: 0.051 g (76%). Yellow solid. Mp: 176–179 °C. ^1^H NMR (CDCl_3_, 400 MHz): δ 7.70 (d, *J* = 7.9 Hz, 1H), 7.45–7.39 (m, 4H), 7.28–7.17 (m, 5H), 7.05 (d, *J* = 7.9 Hz, 1H), 6.87–6.82 (m, 2H), 4.98 (s, 2H), 3.76 (s, 3H). ^13^C­{^1^H} NMR (CDCl_3_, 100 MHz): δ 157.7, 145.8, 143.7, 137.5, 135.0, 134.0, 133.99, 131.3, 129.7, 129.3, 129.1, 128.8, 123.0, 122.5, 122.2, 118.8, 112.1, 111.0, 108.7, 55.9, 49.3. ^77^Se­{^1^H} NMR (CDCl_3_, 76 MHz): δ 349.2. MS (relative intensity (%)): *m*/*z* 450 (20.2), 263 (100.0), 115 (13.6), 77 (12.6). HRMS (APCI-QTOF): *m*/*z* [M + H]^+^ calcd for C_23_H_19_N_2_OSSe, 451.0383; found, 451.0371.

#### 3-((4-Fluorophenyl)­selanyl)-2-phenyl-4*H*-benzo­[4,5]­imidazo­[2,1-*b*]­[1,3]­thiazine (**3e**)

Purified by column chromatography (85:15 hexane/ethyl acetate). Yield: 0.049 g (75%). Orange solid. Mp: 119–121 °C. ^1^H NMR (CDCl_3_, 400 MHz): δ 8.00 (dd, *J* = 8.1 and 0.8 Hz, 1H), 7.69–7.62 (m, 2H), 7.54 (t, *J* = 8.0 Hz, 1H), 7.46–7.40 (m, 5H), 7.27–7.23 (m, 1H), 7.20–7.16 (m, 1H), 7.00–6.96 (m, 2H), 4.86 (s, 2H). ^13^C­{^1^H} NMR (CDCl_3_, 100 MHz): δ 163.1 (d, *J* = 247.9 Hz), 145.4, 143.6, 139.3, 137.2, 135.7 (d, *J* = 8.1 Hz), 134.1, 133.9, 132.5, 129.9, 129.4, 129.3, 128.9, 128.1, 123.1 (d, *J* = 3.5 Hz), 123.0, 122.5, 118.8, 117.1 (d, *J* = 21.6 Hz), 113.2, 108.5, 48.9. ^77^Se­{^1^H} NMR (CDCl_3_, 76 MHz): δ 406.0. ^19^F NMR (376 MHz, CDCl_3_): δ −112.2. MS (relative intensity (%)): *m*/*z* 438 (49.7), 263 (100.0), 115 (32.7), 77 (15.5). HRMS (APCI-QTOF): *m*/*z* [M + H]^+^ calcd for C_22_H_16_FN_2_SSe, 439.0183; found, 439.0174.

#### 3-((4-Chlorophenyl)­selanyl)-2-phenyl-4*H*-benzo­[4,5]­imidazo­[2,1-*b*]­[1,3]­thiazine (**3f**)

Purified by column chromatography (85:15 hexane/ethyl acetate). Yield: 0.054 g (80%). Yellow solid. Mp: 200–203 °C. ^1^H NMR (CDCl_3_, 400 MHz): δ 7.69 (d, *J* = 7.8 Hz, 1H), 7.45–7.40 (m, 5H), 7.34 (d, *J* = 8.5 Hz, 2H), 7.28–7.18 (m, 4H), 7.03 (d, *J* = 7.9 Hz, 1H), 4.91 (s, 2H). ^13^C­{^1^H} NMR (CDCl_3_, 100 MHz): δ 145.3, 143.7, 137.3, 134.7, 134.2, 134.1, 133.9, 130.1, 129.9, 129.4, 128.9, 127.2, 123.1, 122.6, 118.9, 112.5, 108.6, 49.1. ^77^Se­{^1^H} NMR (CDCl_3_, 76 MHz): δ 405.7. MS (relative intensity (%)): *m*/*z* 454 (27.3), 263 (100.0), 115 (26.3), 77 (22.7). HRMS (APCI-QTOF): *m*/*z* [M + H]^+^ calcd for C_22_H_16_ClN_2_SSe, 454.9888; found, 454.9874.

#### 3-((2-Chlorophenyl)­selanyl)-2-phenyl-4*H*-benzo­[4,5]­imidazo­[2,1-*b*]­[1,3]­thiazine (**3g**)

Purified by column chromatography (85:15 hexane/ethyl acetate). Yield: 0.050 g (74%). Yellow solid. Mp: 137–140 °C. ^1^H NMR (CDCl_3_, 400 MHz): δ 7.72 (d, *J* = 7.9 Hz, 1H), 7.44–7.42 (m, 4H), 7.37 (dd, *J* = 7.9 and 1.4 Hz, 1H), 7.31–7.24 (m, 3H), 7.23–7.18 (m, 2H), 7.15–7.11 (m, 2H), 5.03 (s, 2H). ^13^C­{^1^H} NMR (CDCl_3_, 100 MHz): δ 143.7, 137.6, 137.3, 134.0, 130.9, 130.3, 130.0, 129.5, 129.1, 128.9, 128.7, 128.6, 128.2, 123.2, 122.7, 119.0, 111.4, 108.9, 49.6. ^77^Se­{^1^H} NMR (CDCl_3_, 76 MHz): δ 393.2. MS (relative intensity (%)): *m*/*z* 454 (17.8), 263 (100.0), 115 (10.1), 77 (10.5). HRMS (APCI-QTOF): *m*/*z* [M + H]^+^ calcd for C_22_H_16_ClN_2_SSe, 454.9888; found, 454.9875.

#### 2-Phenyl-3-((3-(trifluoromethyl)­phenyl)­selanyl)-4*H*-benzo­[4,5]­imidazo­[2,1-*b*]­[1,3]­thiazine (**3h**)

Purified by column chromatography (85:15 hexane/ethyl acetate). Yield: 0.059 g (80%). White solid. Mp: 120–123 °C. ^1^H NMR (CDCl_3_, 400 MHz): δ 7.70 (d, *J* = 8.0 Hz, 1H), 7.66 (s, 1H), 7.56 (d, *J* = 7.7 Hz, 2H), 7.46–7.40 (m, 5H), 7.38–7.36 (m, 1H), 7.30–7.26 (m, 1H), 7.24–7.20 (m, 1H), 7.06 (d, *J* = 7.8 Hz, 1H), 4.98 (s, 2H). ^13^C­{^1^H} NMR (CDCl_3_, 100 MHz): δ 145.2, 143.7, 137.2, 135.9, 135.4, 133.9, 130.6, 130.3, 130.1, 129.3, 129.2 (q, *J* = 3.7 Hz), 129.0, 125.0 (q, *J* = 3.7 Hz), 123.2, 122.7, 119.0, 111.8, 108.6, 49.5. ^77^Se­{^1^H} NMR (CDCl_3_, 76 MHz): δ 413.3. ^19^F NMR (376 MHz, CDCl_3_): δ −62.8. MS (relative intensity (%)): *m*/*z* 488 (39.8), 263 (100.0), 115 (23.5), 77 (10.8). HRMS (APCI-QTOF): *m*/*z* [M + H]^+^ calcd for C_23_H_16_F_3_N_2_SSe, 489.0152; found, 489.0142.

#### 3-(Butylselanyl)-2-phenyl-4*H*-benzo­[4,5]­imidazo­[2,1-*b*]­[1,3]­thiazine (**3i**)

Purified by column chromatography (85:15 hexane/ethyl acetate). Yield: 0.043 g (72%). Orange oil. ^1^H NMR (CDCl_3_, 400 MHz): δ 7.71–7.68 (m, 1H), 7.43–7.37 (m, 5H), 7.34–7.31 (m, 1H), 7.30–7.26 (m, 2H), 5.11 (s, 2H), 2.67 (t, *J* = 7.4 Hz, 2H), 1.52 (quint, *J* = 7.4 Hz, 2H), 1.32–1.23 (m, 2H), 0.83 (t, *J* = 7.4 Hz, 3H). ^13^C­{^1^H} NMR (CDCl_3_, 100 MHz): δ 145.6, 143.6, 137.8, 134.1, 132.1, 129.5, 129.46, 128.7, 111.4, 108.6, 49.7, 32.3, 27.1, 22.8, 13.6. ^77^Se­{^1^H} NMR (CDCl_3_, 76 MHz): δ 287.2. MS (relative intensity (%)): *m*/*z* 400 (31.6), 343 (5.3), 263 (100.0), 115 (20.2), 77 (6.5). HRMS (APCI-QTOF): *m*/*z* [M + H]^+^ calcd for C_20_H_21_N_2_SSe, 401.0591; found, 401.0582.

#### 2-Phenyl-3-(thiophen-2-ylselanyl)-4*H*-benzo­[4,5]­imidazo­[2,1-*b*]­[1,3]­thiazine (**3j**)

Purified by column chromatography (85:15 hexane/ethyl acetate). Yield: 0.043 g (67%). Orange solid. Mp: 135–138 °C. ^1^H NMR (CDCl_3_, 400 MHz): δ 7.67 (d, *J* = 7.7 Hz, 1H), 7.52–7.51 (m, 1H), 7.49–7.46 (m, 4H), 7.29–7.28 (m, 1H), 7.26–7.18 (m, 3H), 7.06 (dd, *J* = 5.3 Hz, 1H), 7.01 (d, *J* = 8.0 Hz, 1H), 4.86 (s, 2H). ^13^C­{^1^H} NMR (CDCl_3_, 100 MHz): δ 143.7, 138.3, 137.0, 134.1, 133.2, 130.0, 129.5, 129.4, 129.1, 128.8, 123.0, 122.4, 121.1, 118.9, 114.2, 108.5, 50.0. ^77^Se­{^1^H} NMR (CDCl_3_, 76 MHz): δ 321.3. MS (relative intensity (%)): *m*/*z* 426 (42.5), 345 (41.2), 263 (42.2), 207 (100.0), 115 (68.0), 77 (20.4). HRMS (APCI-QTOF): *m*/*z* [M + H]^+^ calcd for C_20_H_15_N_2_S_2_Se, 426.9842; found, 426.9831.

#### 3-(Phenylselanyl)-2-(4-tolyl)-4*H*-benzo­[4,5]­imidazo­[2,1-*b*]­[1,3]­thiazine (**3l**)

Purified by column chromatography (85:15 hexane/ethyl acetate). Yield: 0.051 g (78%). Yellow solid. Mp: 106–109 °C. ^1^H NMR (CDCl_3_, 400 MHz): δ 7.68 (d, *J* = 7.9 Hz, 1H), 7.43 (dd, *J* = 7.6 and 1.4 Hz, 2H), 7.35 (d, *J* = 8.1 Hz, 2H), 7.31–7.22 (m, 6H), 7.16 (td, *J* = 8.1 and 1.1 Hz, 1H), 6.96 (d, *J* = 7.9 Hz, 1H), 4.89 (s, 2H), 2.40 (s, 3H). ^13^C­{^1^H} NMR (CDCl_3_, 100 MHz): δ 145.9, 143.7, 140.0, 134.5, 133.9, 133.2, 133.0, 129.9, 129.6, 129.3, 129.0, 128.3, 123.0, 122.4, 118.8, 112.8, 108.6, 49.0, 21.6. ^77^Se­{^1^H} NMR (CDCl_3_, 76 MHz): δ 412.5. MS (relative intensity (%)): *m*/*z* 434 (49.8), 277 (100.0), 115 (20.6), 77 (28.9). HRMS (APCI-QTOF): *m*/*z* [M + H]^+^ calcd for C_23_H_19_N_2_SSe, 435.0434; found, 435.0424.

#### 2-(4-Methoxyphenyl)-3-(phenylselanyl)-4*H*-benzo­[4,5]­imidazo­[2,1-*b*]­[1,3]­thiazine (**3m**)

Purified by column chromatography (85:15 hexane/ethyl acetate). Yield: 0.051 g (76%). Yellow oil. ^1^H NMR (CDCl_3_, 400 MHz): δ 7.68 (d, *J* = 8.0 Hz, 1H), 7.44–7.38 (m, 4H), 7.31–7.23 (m, 4H), 7.19–7.15 (m, 1H), 6.97–6.95 (m, 3H), 4.89 (s, 2H), 3.85 (s, 3H). ^13^C­{^1^H} NMR (CDCl_3_, 100 MHz): δ 160.8, 146.1, 143.8, 134.0, 133.0, 131.0, 129.9, 129.6, 129.1, 128.3, 123.0, 122.4, 118.8, 114.2, 112.8, 108.6, 55.6, 49.0. ^77^Se­{^1^H} NMR (CDCl_3_, 76 MHz): δ 409.9. MS (relative intensity (%)): *m*/*z* 450 (5.7), 269 (6.3), 281 (29.4), 207 (100.0), 77 (2.1). HRMS (APCI-QTOF): *m*/*z* [M + H]^+^ calcd for C_23_H_19_N_2_OSSe, 451.0383; found, 451.0387.

#### 2-(4-Chlorophenyl)-3-(phenylselanyl)-4*H*-benzo­[4,5]­imidazo­[2,1-*b*]­[1,3]­thiazine (**3n**)

Purified by column chromatography (85:15 hexane/ethyl acetate). Yield: 0.049 g (72%). Yellow solid. Mp: 159–162 °C. ^1^H NMR (CDCl_3_, 400 MHz): δ 7.68 (d, *J* = 7.9 Hz, 1H), 7.43–7.37 (m, 6H), 7.33–7.23 (m, 4H), 7.18 (td, *J* = 8.1 and 1.1 Hz, 1H), 6.97 (d, *J* = 7.9 Hz, 1H), 4.89 (s, 2H). ^13^C­{^1^H} NMR (CDCl_3_, 100 MHz): δ 143.7, 135.9, 135.7, 133.1, 131.9, 130.8, 130.0, 129.3, 129.2, 128.6, 128.5, 128.2, 123.1, 122.6, 118.9, 114.0, 108.6, 49.0. ^77^Se­{^1^H} NMR (CDCl_3_, 76 MHz): δ 411.8. MS (relative intensity (%)): *m*/*z* 454 (70.6), 297 (100.0), 262 (78.9), 115 (23.2), 77 (34.8). HRMS (APCI-QTOF): *m*/*z* [M + H]^+^ calcd for C_22_H_16_ClN_2_SSe, 454.9888; found, 454.9874.

#### 2-(4-Bromophenyl)-3-(phenylselanyl)-4*H*-benzo­[4,5]­imidazo­[2,1-*b*]­[1,3]­thiazine (**3o**)

Purified by column chromatography (85:15 hexane/ethyl acetate). Yield: 0.058 g (78%). White solid. Mp: 174–177 °C. ^1^H NMR (CDCl_3_, 400 MHz): δ 7.68 (d, *J* = 8.0 Hz, 1H), 7.58 (d, *J* = 8.5 Hz, 2H), 7.44–7.41 (m, 2H), 7.33–7.23 (m, 6H), 7.20–7.16 (m, 1H), 6.98 (d, *J* = 7.9 Hz, 1H), 4.90 (s, 2H). ^13^C­{^1^H} NMR (CDCl_3_, 100 MHz): δ 145.2, 143.7, 136.2, 133.9, 133.1, 132.2, 132.0, 131.1, 130.0, 128.6, 128.55, 124.2, 123.1, 122.6, 118.9, 114.0, 108.6, 49.1. ^77^Se­{^1^H} NMR (CDCl_3_, 76 MHz): δ 412.3. MS (relative intensity (%)): *m*/*z* 498 (6.8), 341 (7.3), 281 (30.0), 207 (100.0), 77 (4.0). HRMS (APCI-QTOF): *m*/*z* [M + H]^+^ calcd for C_22_H_16_BrN_2_SSe, 498.9383; found, 498.9368.

#### 3-(Phenylselanyl)-2-(2-tolyl)-4*H*-benzo­[4,5]­imidazo­[2,1-*b*]­[1,3]­thiazine (**3p**)

Purified by column chromatography (85:15 hexane/ethyl acetate). Yield: 0.048 g (75%). White solid. Mp: 137–139 °C. ^1^H NMR (CDCl_3_, 400 MHz): δ 7.60 (d, *J* = 7.9 Hz, 1H), 7.40–7.37 (m, 2H), 7.28–7.15 (m, 8H), 7.12–7.08 (m, 1H), 7.93 (d, *J* = 7.9 Hz, 1H), 4.80 (dd, *J* = 27.6 and 16.5 Hz, 2H), 2.32 (s, 3H). ^13^C­{^1^H} NMR (CDCl_3_, 100 MHz): δ 145.4, 143.6, 136.7, 136.6, 134.1, 133.7, 130.85, 130.8, 129.9, 129.8, 129.7, 128.6, 128.0, 126.5, 123.0, 122.4, 118.7, 113.6, 108.6, 48.6, 19.5. ^77^Se­{^1^H} NMR (CDCl_3_, 76 MHz): δ 415.6. MS (relative intensity (%)): *m*/*z* 434 (59.3), 277 (100.0), 115 (62.6), 77 (30.2). HRMS (APCI-QTOF): *m*/*z* [M + H]^+^ calcd for C_23_H_19_N_2_SSe, 435.0434; found, 435.0424.

#### 2-(2-Iodophenyl)-3-(phenylselanyl)-4*H*-benzo­[4,5]­imidazo­[2,1-*b*]­[1,3]­thiazine (**3q**)

Purified by column chromatography (85:15 hexane/ethyl acetate). Yield: 0.033 g (40%). White oil. ^1^H NMR (CDCl_3_, 400 MHz): δ 7.88 (d, *J* = 8.0 Hz, 1H), 7.62 (d, *J* = 7.9 Hz, 1H), 7.49–7.47 (m, 2H), 7.39–7.35 (m, 1H), 7.31–7.17 (m, 5H), 7.14–7.10 (m, 1H), 7.06 (td, *J* = 8.0 and 2.0 Hz, 1H), 6.96 (d, *J* = 7.9 Hz, 1H), 4.84 (dd, *J* = 31.9 and 16.6 Hz, 2H). ^13^C­{^1^H} NMR (CDCl_3_, 100 MHz): δ 145.1, 143.6, 141.8, 139.9, 134.1, 133.9, 131.0, 130.5, 129.9, 128.9, 128.7, 128.1, 123.1, 122.5, 118.9, 115.8, 108.6, 99.7, 48.7. ^77^Se­{^1^H} NMR (CDCl_3_, 76 MHz): δ 419.6. MS (relative intensity (%)): *m*/*z* 546 (28.3), 419 (13.9), 277 (100.0), 115 (57.6), 77 (45.2). HRMS (APCI-QTOF): *m*/*z* [M + H]^+^ calcd for C_22_H_16_IN_2_SSe, 546.9244; found, 546.9233.

#### 2-(Methylsulfinyl)-1-(3-phenylprop-2-yn-1-yl)-1*H*-benzo­[*d*]­imidazole (**7**)

Purified by column chromatography (85:15 hexane/ethyl acetate). Yield: 0.022 g (51%). Yellow oil. ^1^H NMR (CDCl_3_, 400 MHz): δ 7.77 (d, *J* = 8.0 Hz, 1H), 7.59 (d, *J* = 8.0 Hz, 1H), 7.38 (td, *J* = 7.2 and 1.1 Hz, 1H), 7.33–7.29 (m, 3H), 7.25–7.18 (m, 3H), 5.71 (d, *J* = 18.2 Hz, 1H), 5.49 (d, *J* = 18.2 Hz, 1H), 3.21 (s, 3H). ^13^C­{^1^H} NMR (CDCl_3_, 100 MHz): δ 151.1, 142.1, 136.1, 132.0, 129.2, 125.3, 124.0, 121.9, 121.3, 110.8, 86.1, 82.0, 40.3, 34.8. MS (relative intensity (%)): *m*/*z* 294 (2.8), 278 (40.7), 163 (10.6), 115 (100.0), 77 (8.4). HRMS (APCI-QTOF): *m*/*z* [M + H]^+^ calcd for C_17_H_15_N_2_OS, 295.0905; found, 295.0897.

## Supplementary Material





## Data Availability

The data underlying this study are available in the published article and its .

## Notes

FAIR data, including the primary NMR FID files, for compounds **1a**–**h**, **3a**–**q**, and **7** (ZIP)
Copies of ^1^H, ^13^C­{^1^H}, ^19^F and ^77^Se­{^1^H} spectra of all the synthesized compounds (PDF)
