# Supplementary material for: Synthesis of 2‑Aryl-3-(organoselanyl)‑4H‑benzo[4,5]imidazo[2,1‑b][1,3]thiazines Promoted by N‑Fluorobenzenesulfonimide (NFSI)
Source: J Org Chem. 2025 Sep 29;90(40):14132–40. doi: 10.1021/acs.joc.5c01598 (PMC12519491; doi:10.1021/acs.joc.5c01598)
Supplement: Supplementary file 2 [file jo5c01598_si_002.pdf]

Synthesis of 2-Aryl-3-(organoselanyl)-4*H*-benzo[4,5]imidazo[2,1-*b*][1,3]thiazines Promoted by *N*-Fluorobenzenesulfonimide (NFSI)

Ricardo H. Bartz,[a] Pedro S. Souza,[a] Leonardo Rocha,[a] Raquel G. Jacob,[a] Eder J. Lenardão,[a] Márcio S. Silva,*[a] and Gelson Perin*[a]

Contents

Copies of (^{1}H, ^{13}C and ^{77}Se) NMR Spectra………………………..…………………..........S2

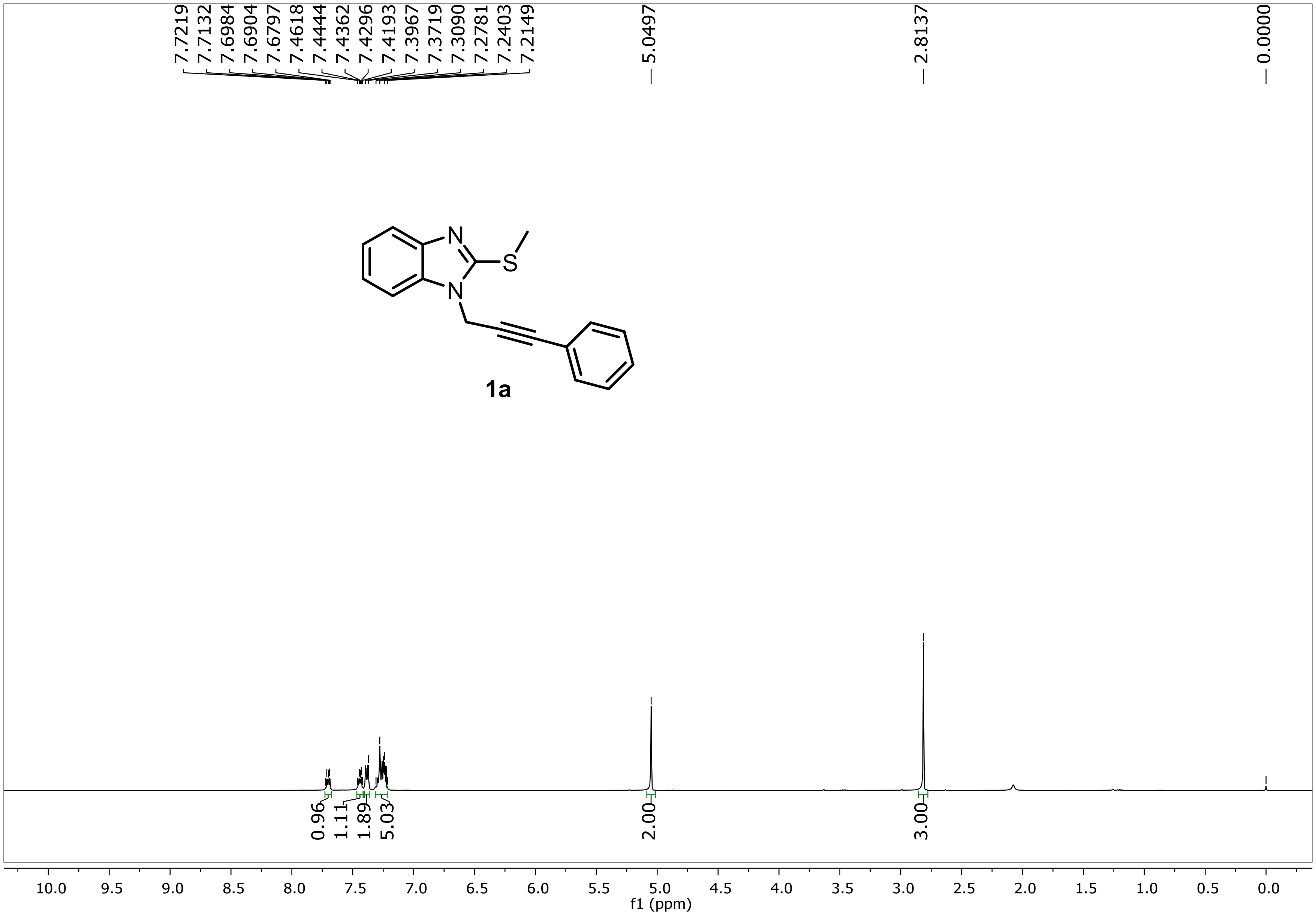

Figure S1: ^{1}H NMR (400 MHz, $CDCl_3$) spectrum of compound **1a**.

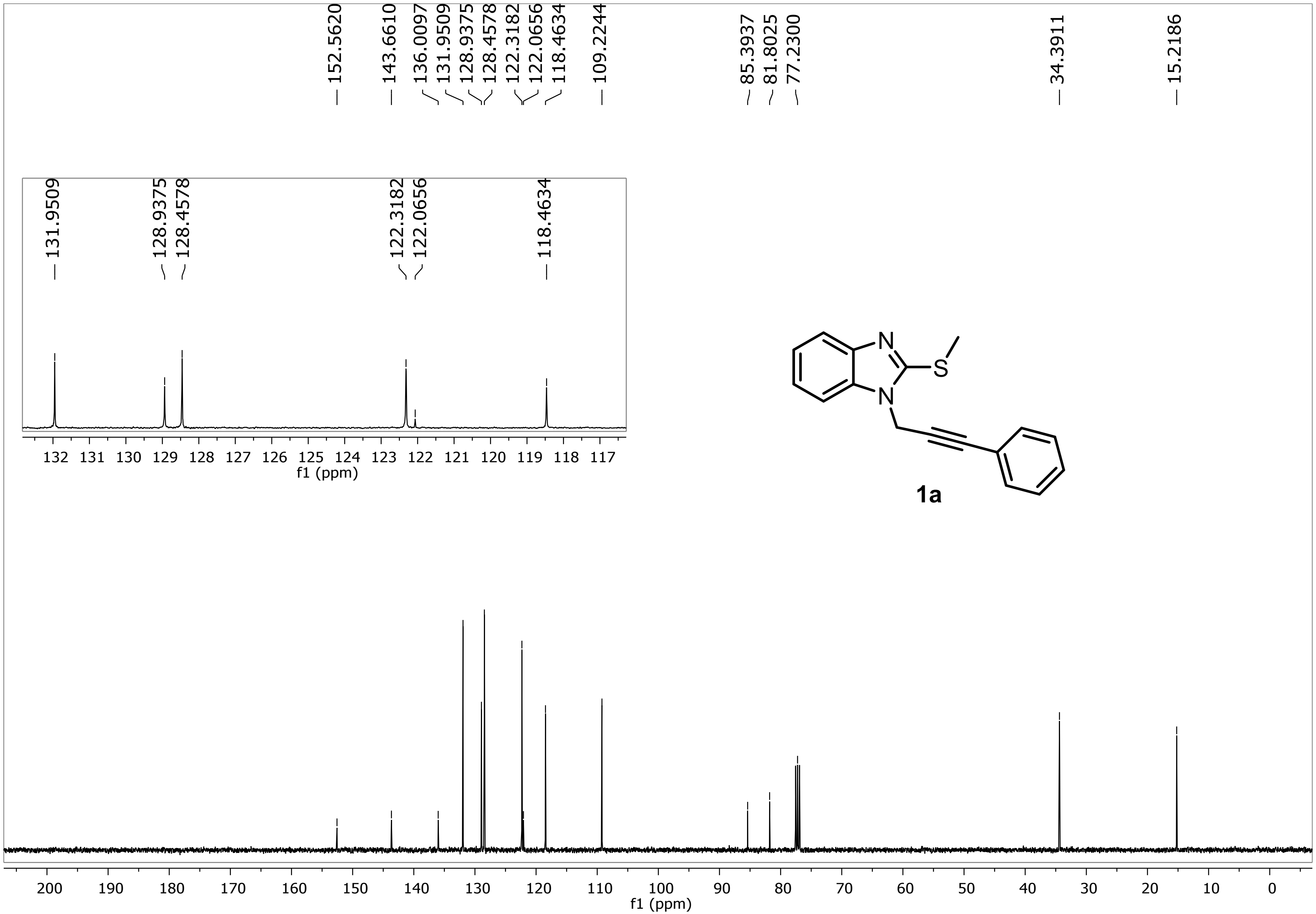

Figure S2: $^{13}C\{^{1}H\}$ NMR (100 MHz, $CDCl_3$) spectrum of compound **1b**.

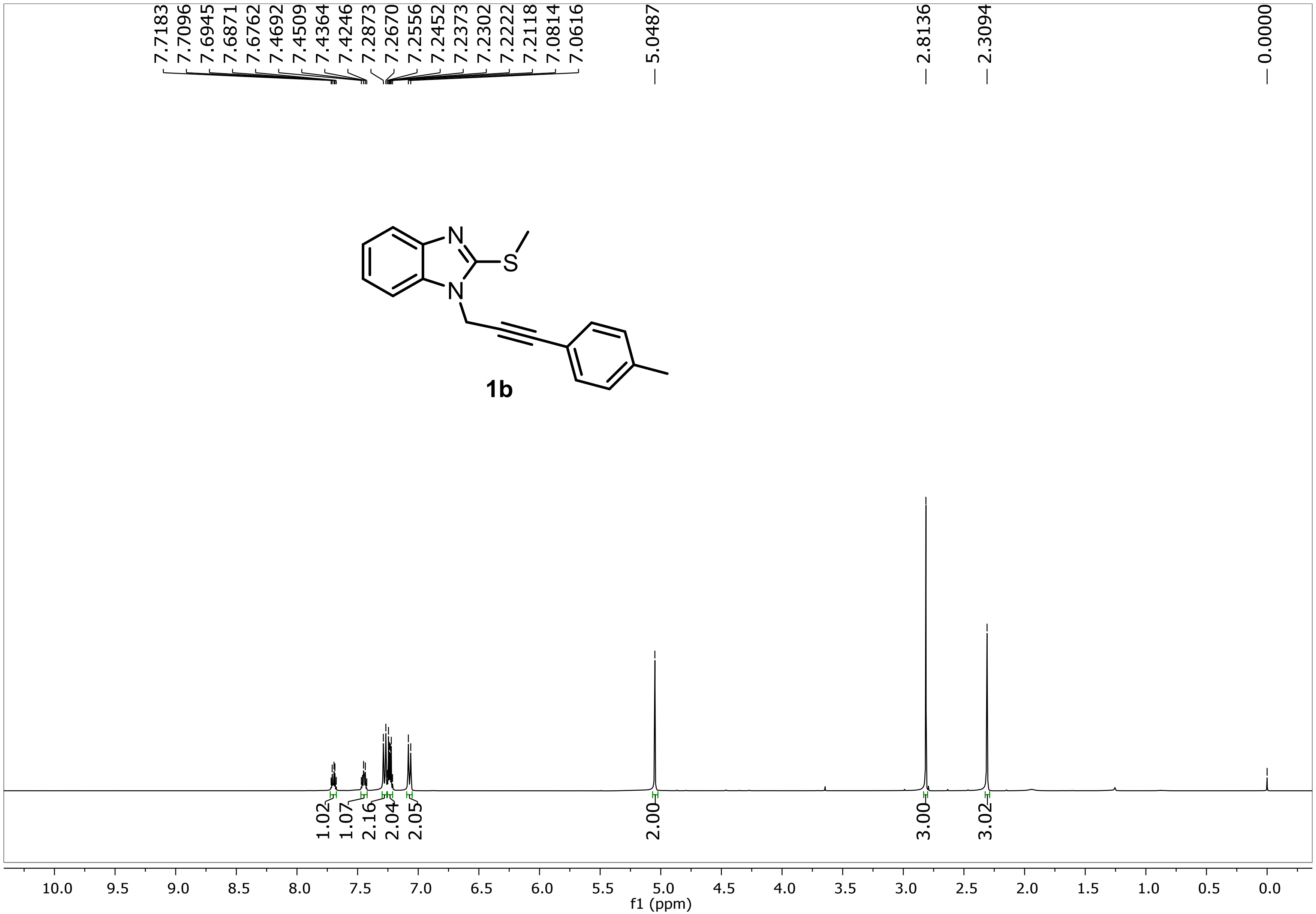

Figure S3: ^{1}H NMR (400 MHz, $CDCl_3$) spectrum of compound **1b**.

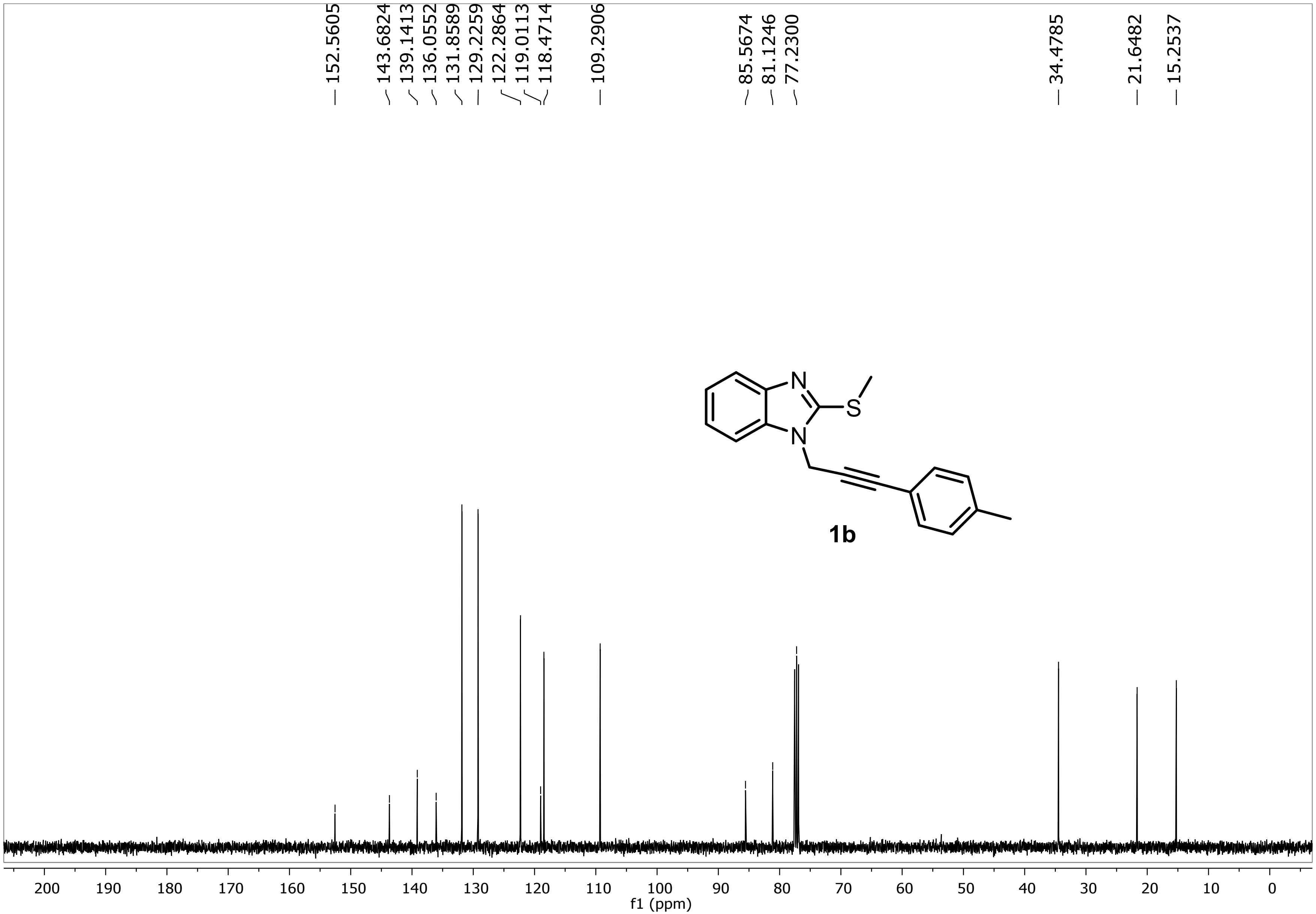

Figure S4: $^{13}C\{^{1}H\}$ NMR (100 MHz, $CDCl_3$) spectrum of compound **1b**.

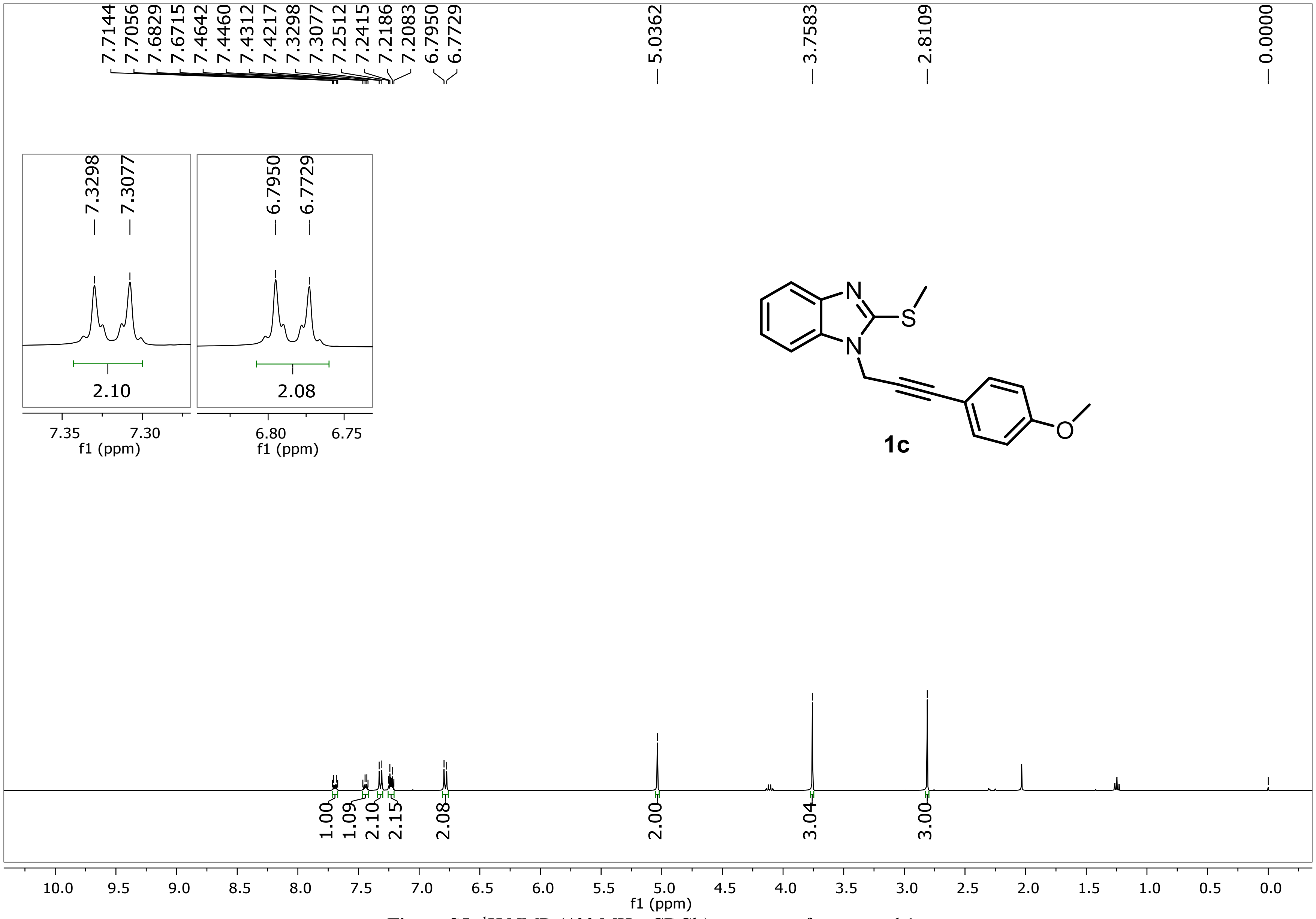

Figure S5: ^{1}H NMR (400 MHz, $CDCl_3$) spectrum of compound **1c**.

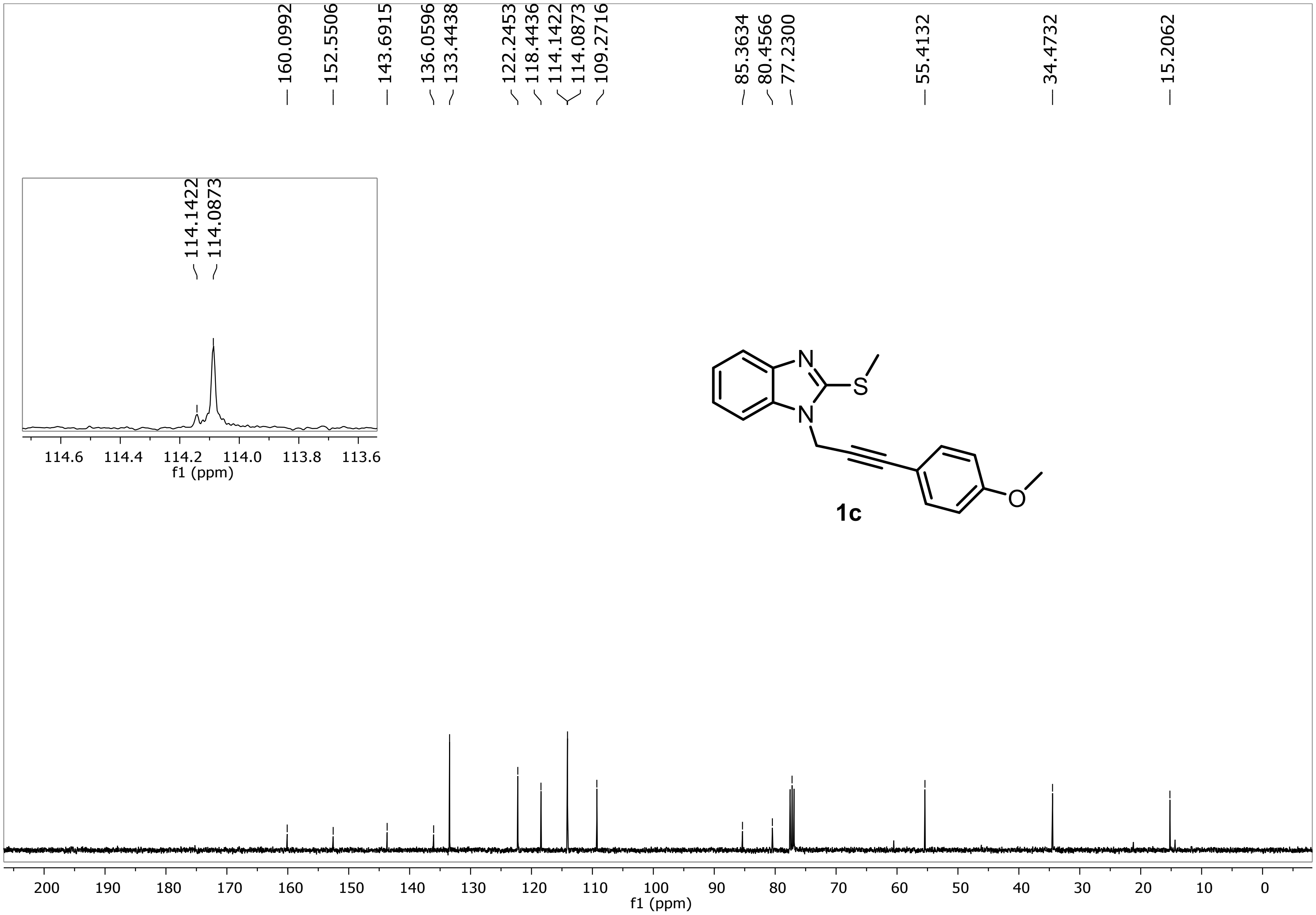

Figure S6: $^{13}C\{^{1}H\}$ NMR (100 MHz, $CDCl_3$) spectrum of compound **1c**.

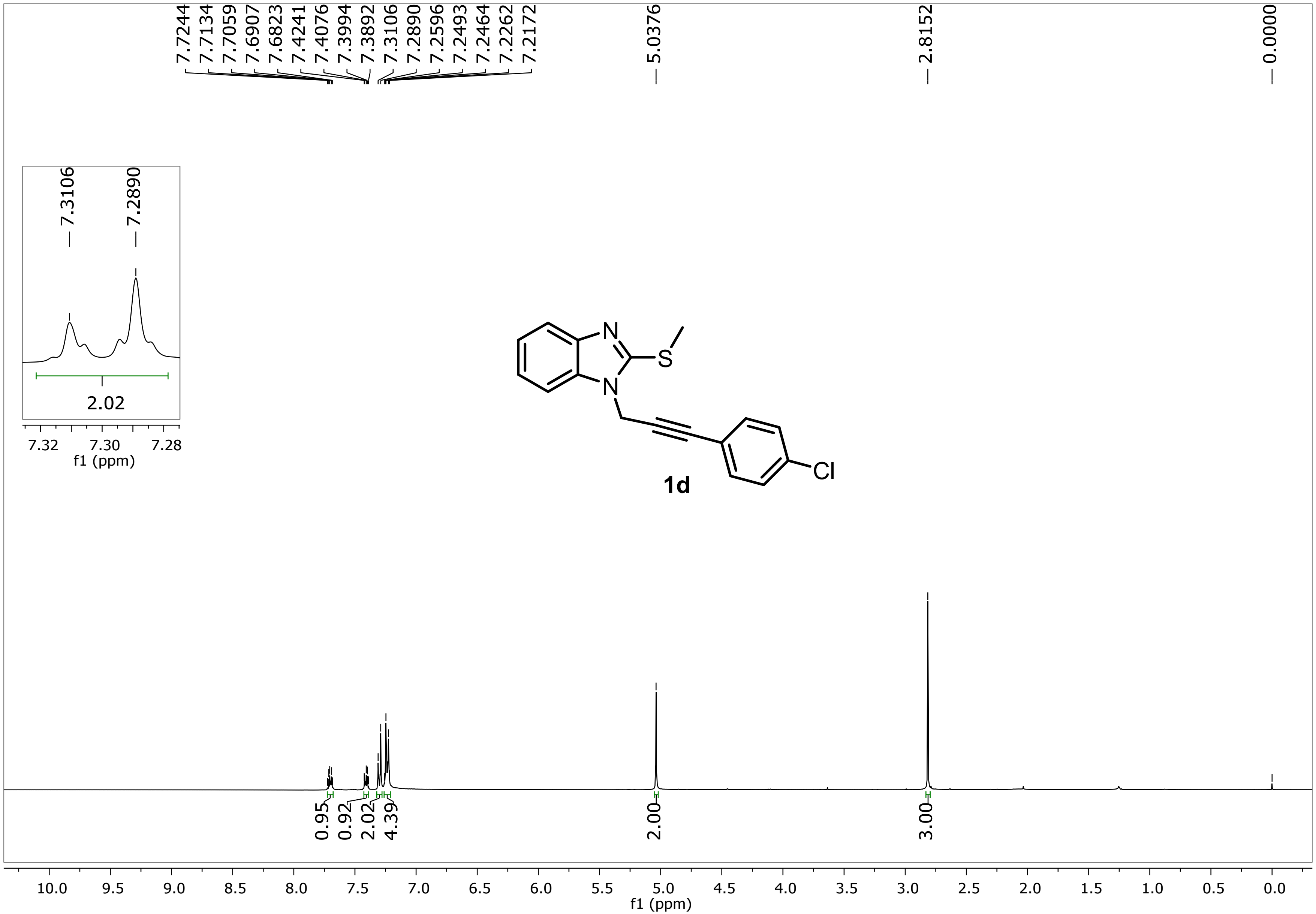

Figure S7: ^{1}H NMR (400 MHz, $CDCl_3$) spectrum of compound **1d**.

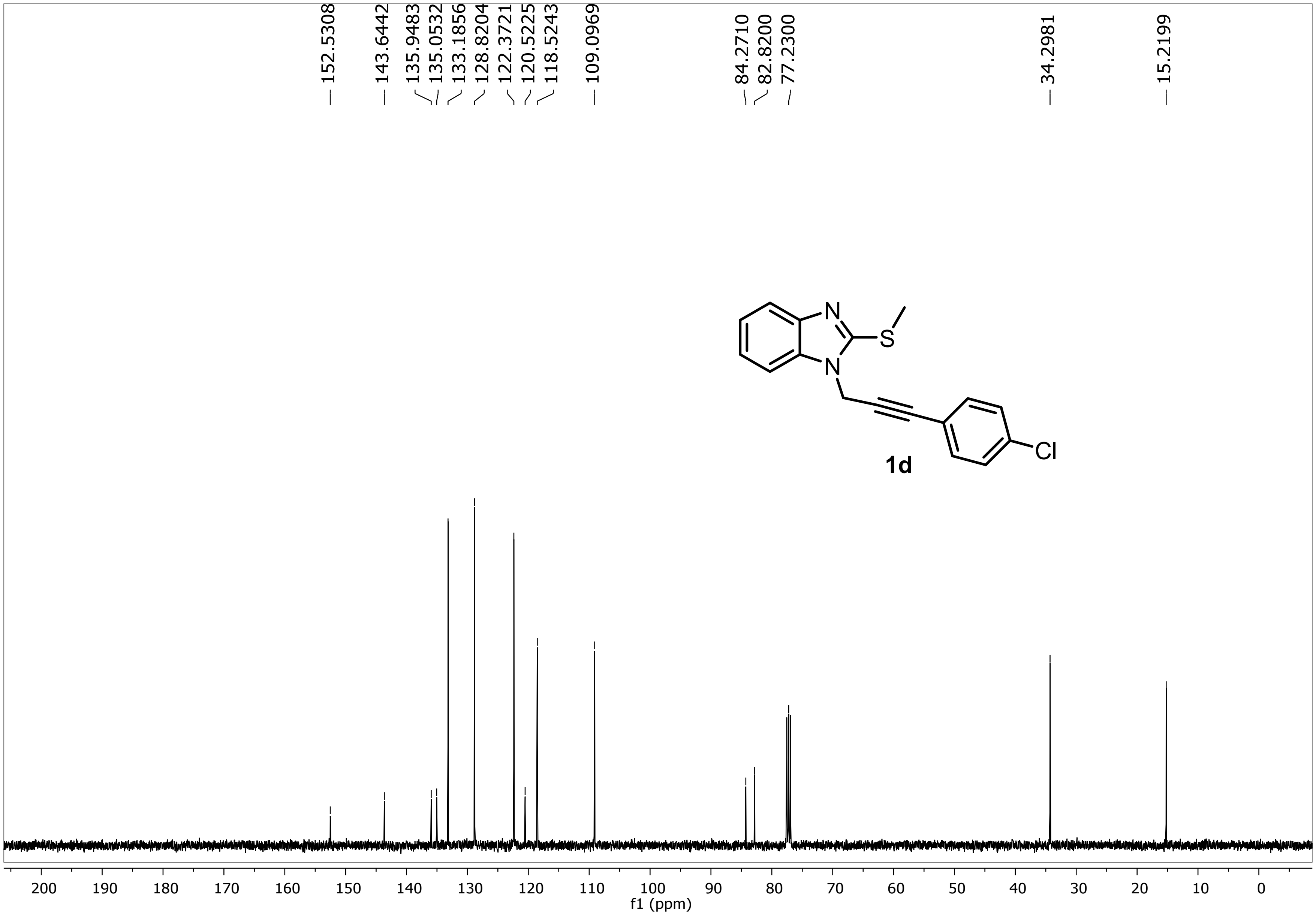

Figure S8: $^{13}C\{^{1}H\}$ NMR (100 MHz, $CDCl_3$) spectrum of compound **1d**.

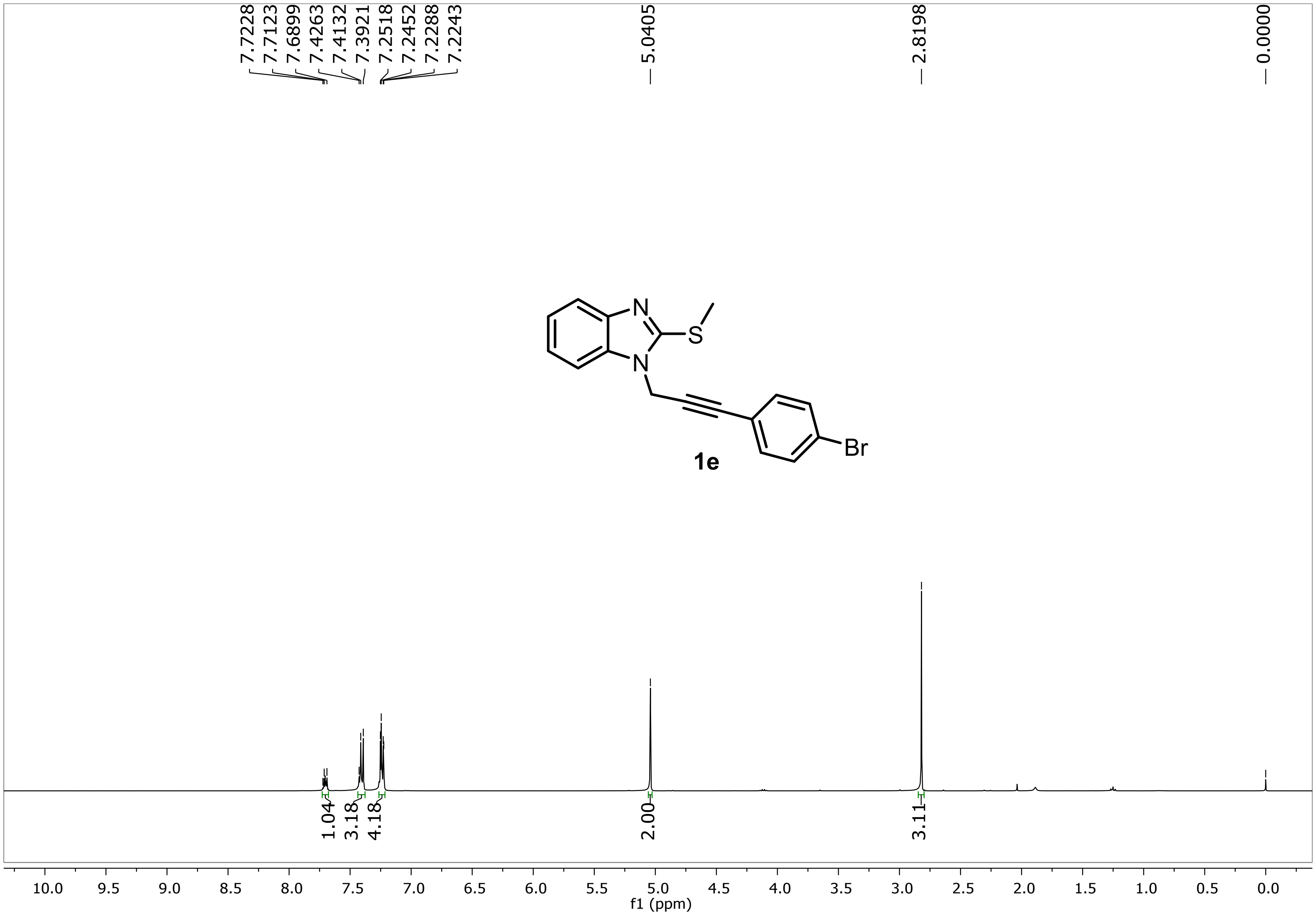

Figure S9: ^{1}H NMR (400 MHz, $CDCl_3$) spectrum of compound **1e**.

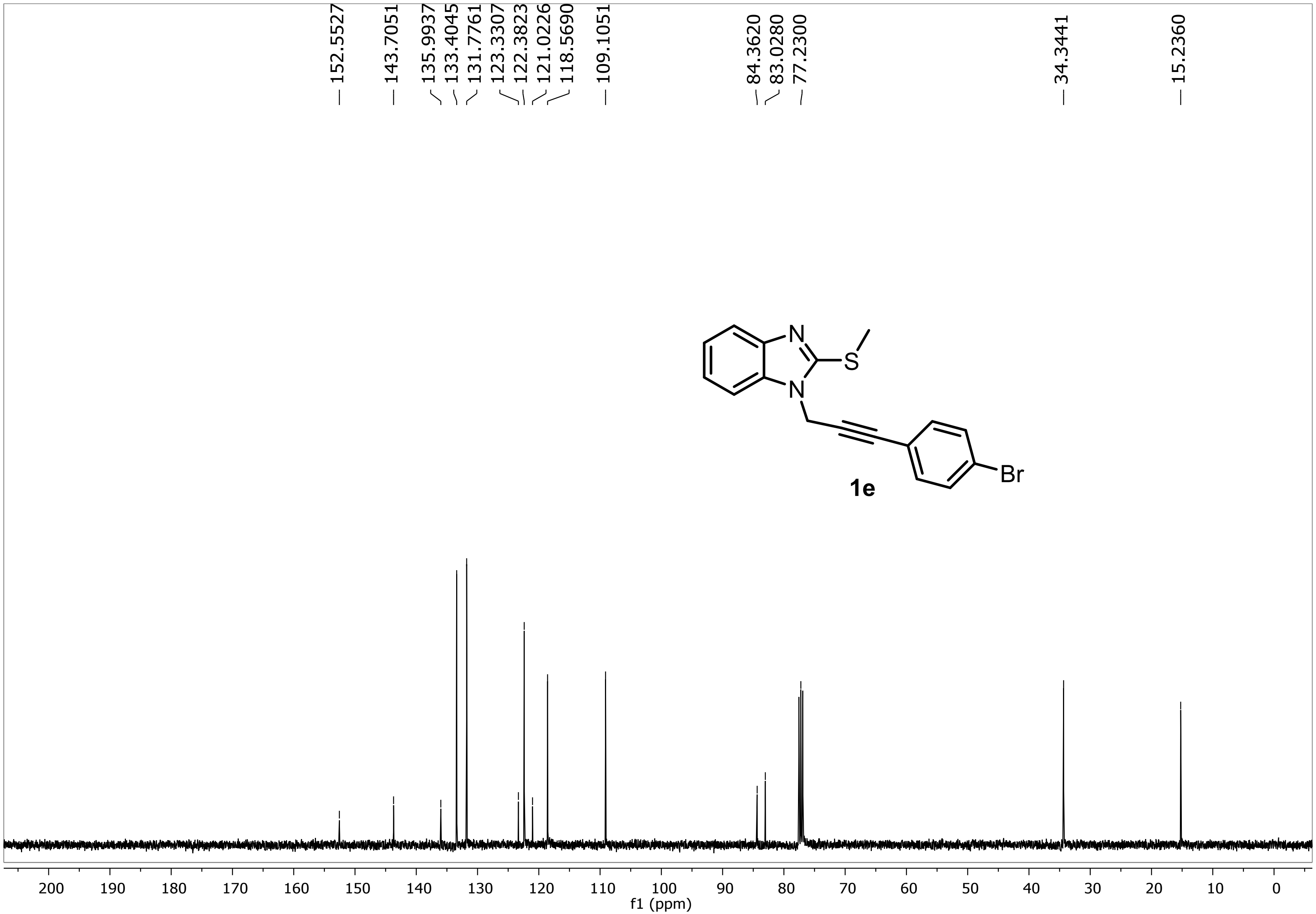

Figure S10: $^{13}C\{^1H\}$ NMR (100 MHz, $CDCl_3$) spectrum of compound **1e**.

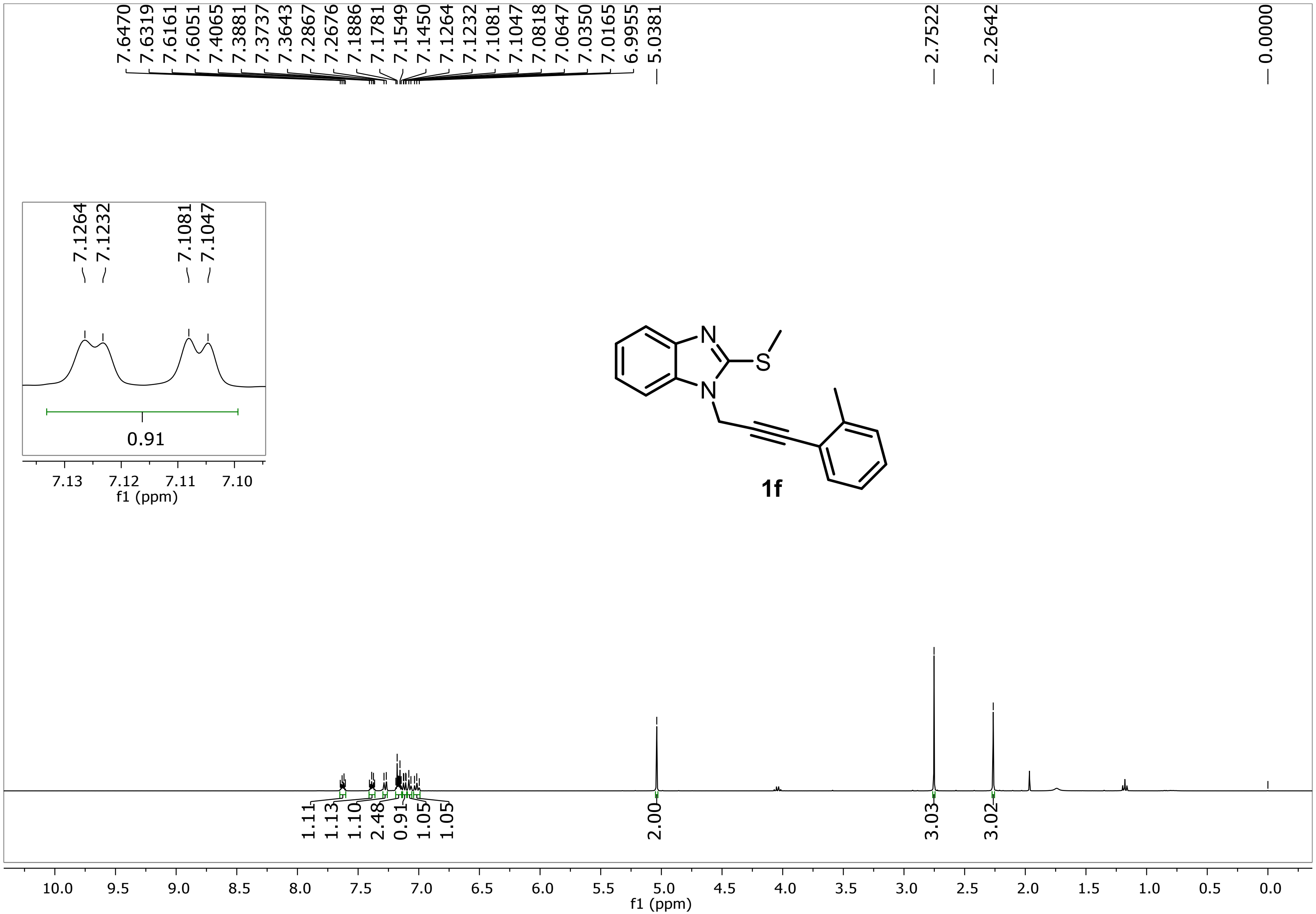

1f

Figure S11: ^{1}H NMR (400 MHz, $CDCl_3$) spectrum of compound **1f**.

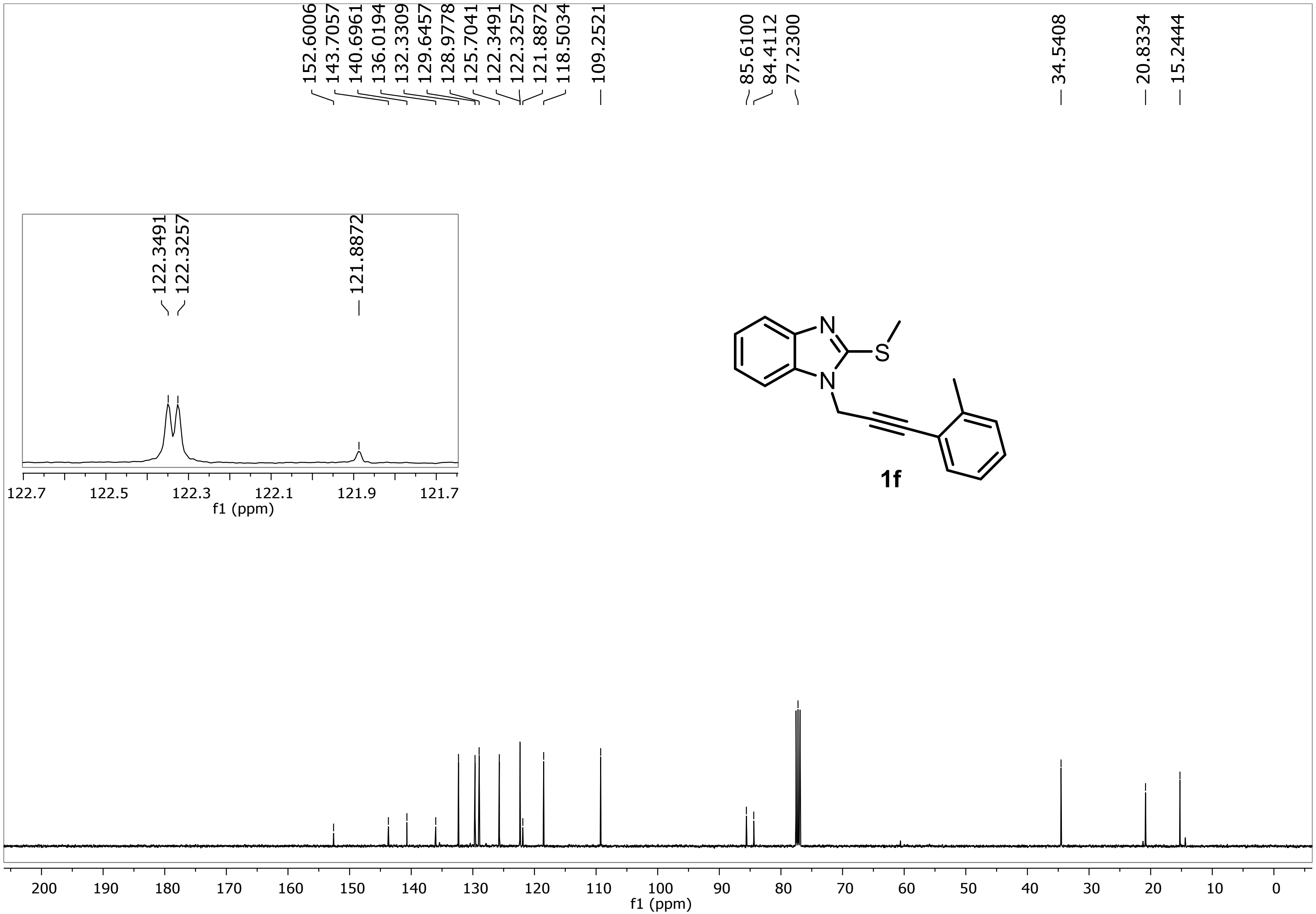

1f

Figure S12: $^{13}C\{^1H\}$ NMR (100 MHz, $CDCl_3$) spectrum of compound **1f**.

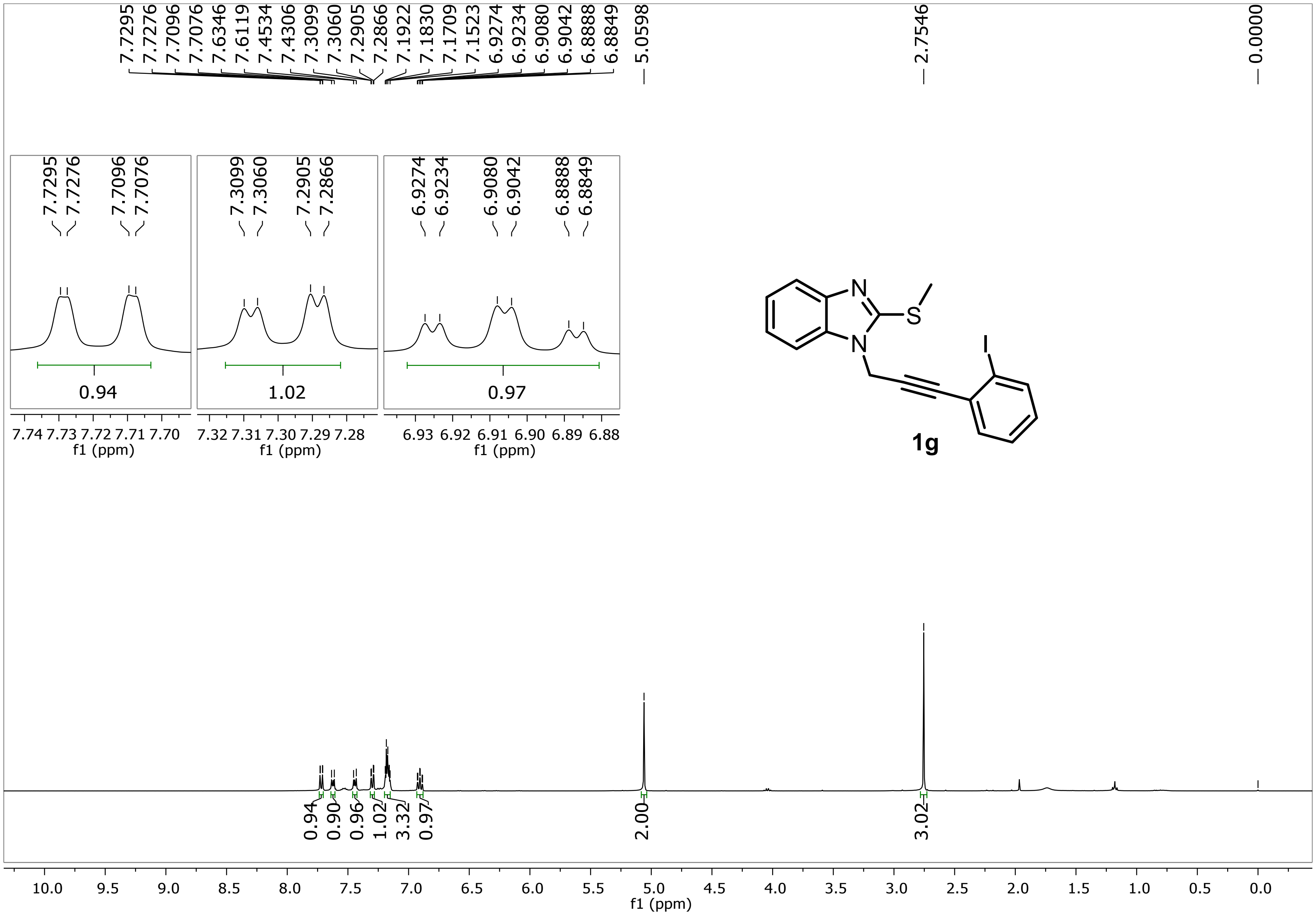

Figure S13: ^{1}H NMR (400 MHz, $CDCl_3$) spectrum of compound **1g**.

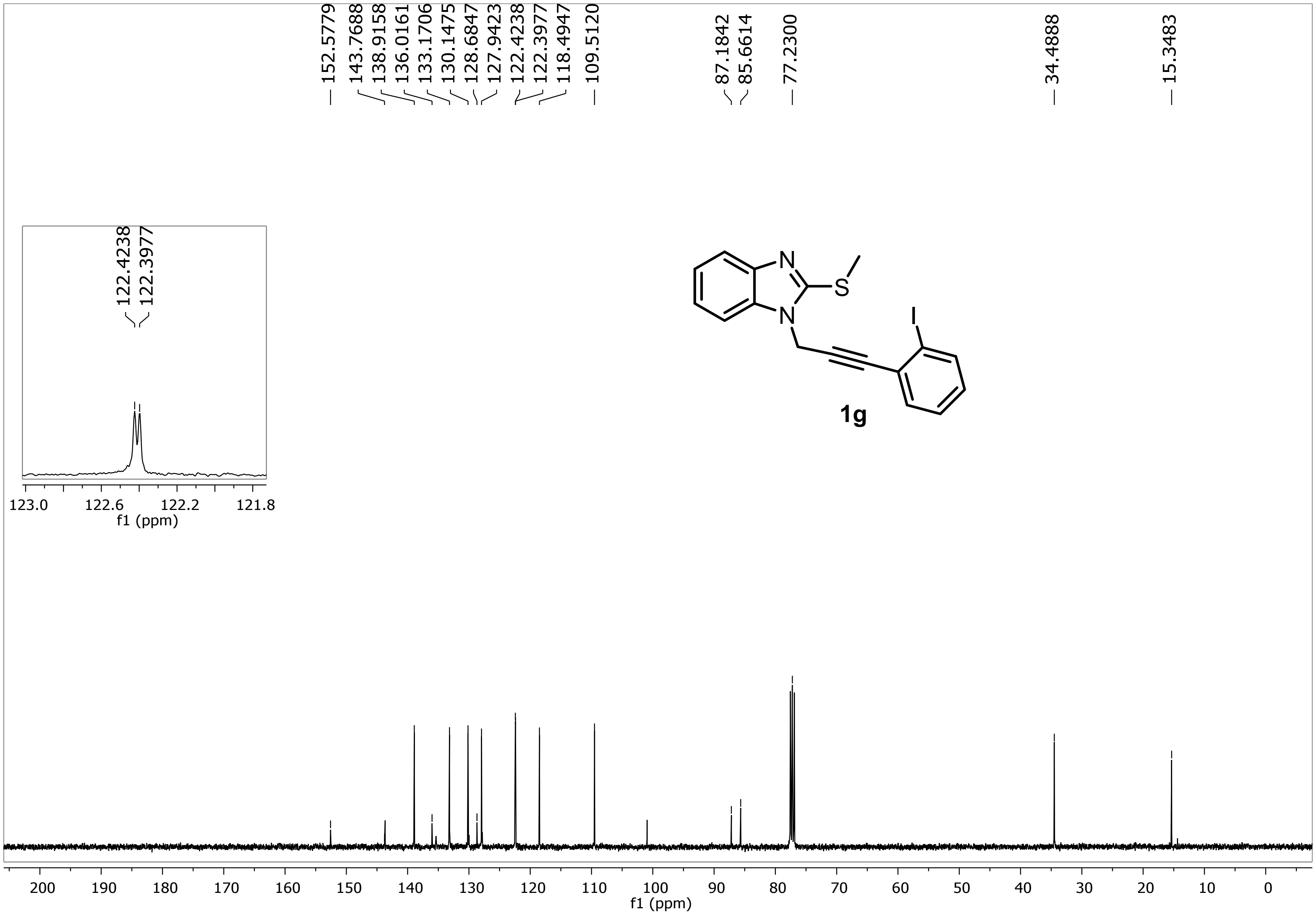

Figure S14: $^{13}C\{^{1}H\}$ NMR (100 MHz, $CDCl_3$) spectrum of compound **1g**.

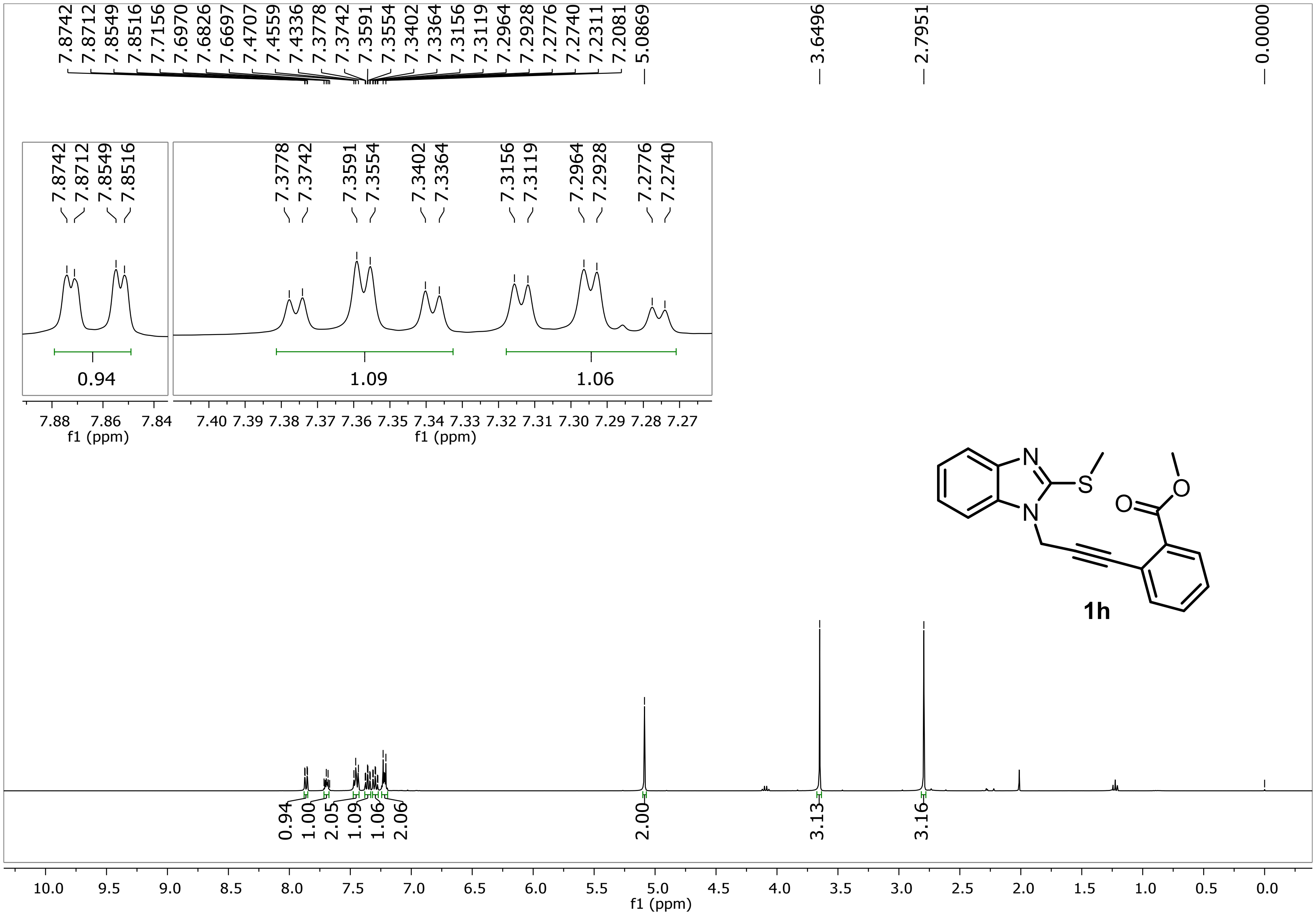

Figure S15: ^{1}H NMR (400 MHz, $CDCl_3$) spectrum of compound **1h**.

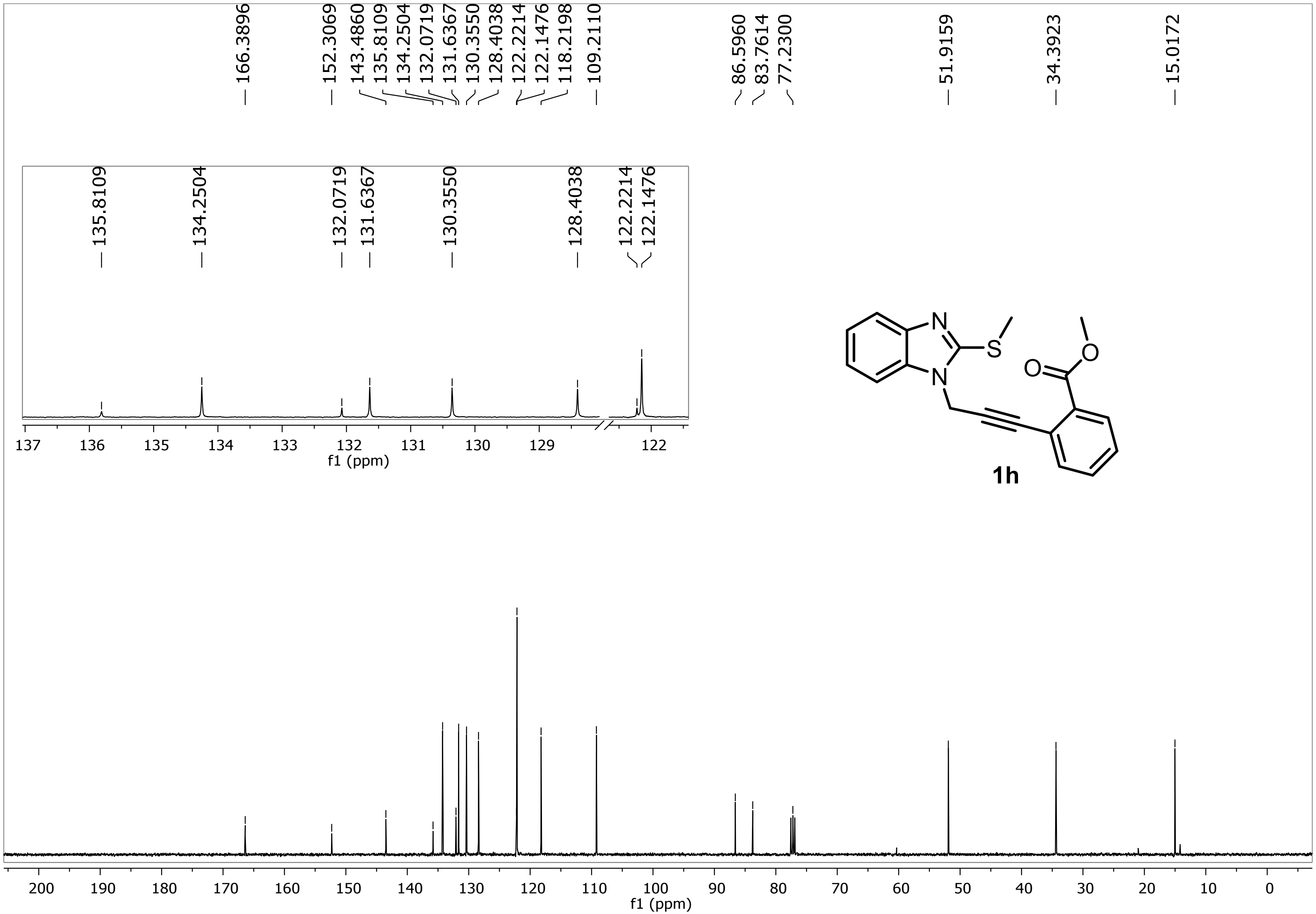

Figure S16: $^{13}C\{^{1}H\}$ NMR (100 MHz, $CDCl_3$) spectrum of compound **1h**.

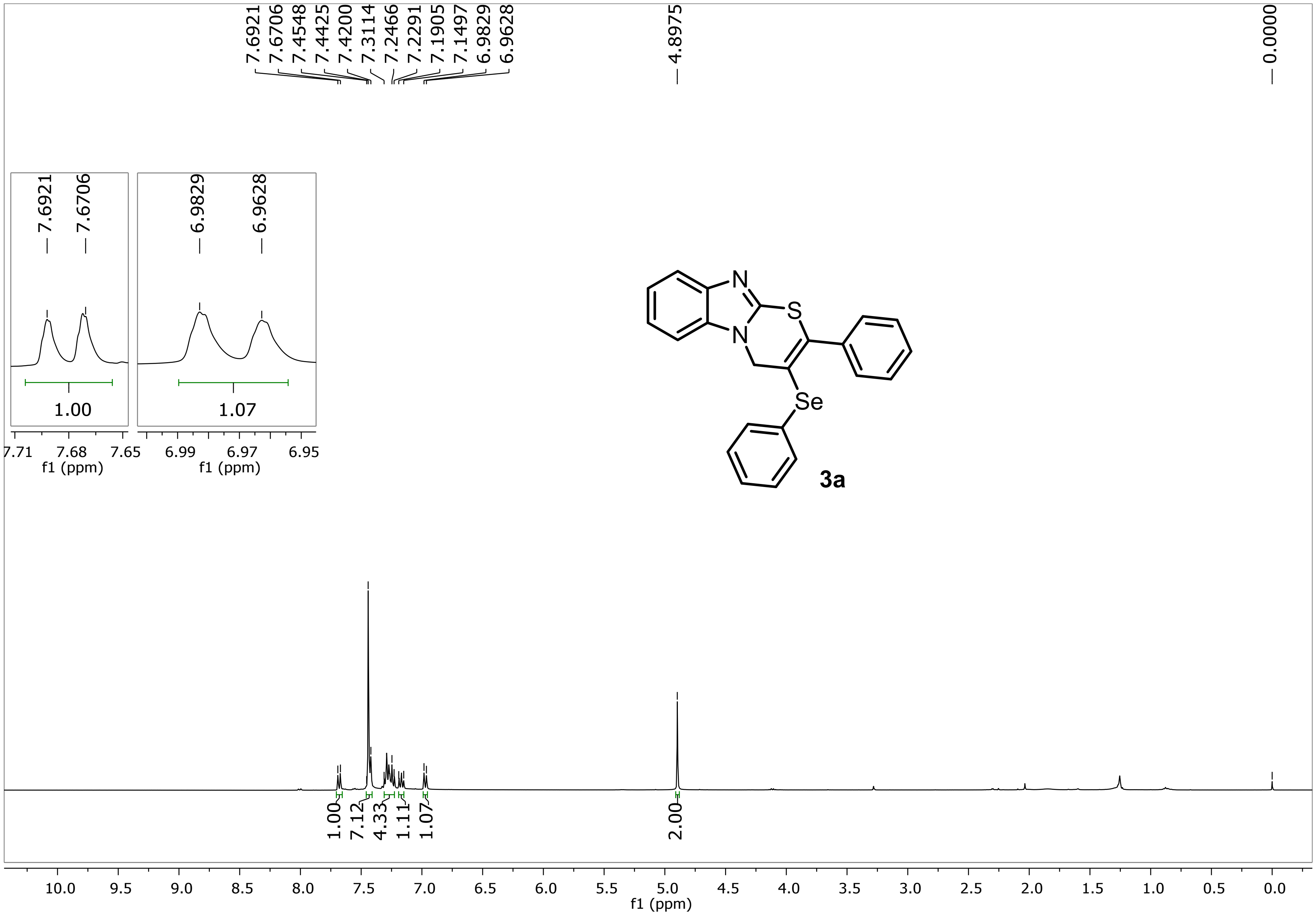

Figure S17: ^{1}H NMR (400 MHz, $CDCl_3$) spectrum of compound **3a**.

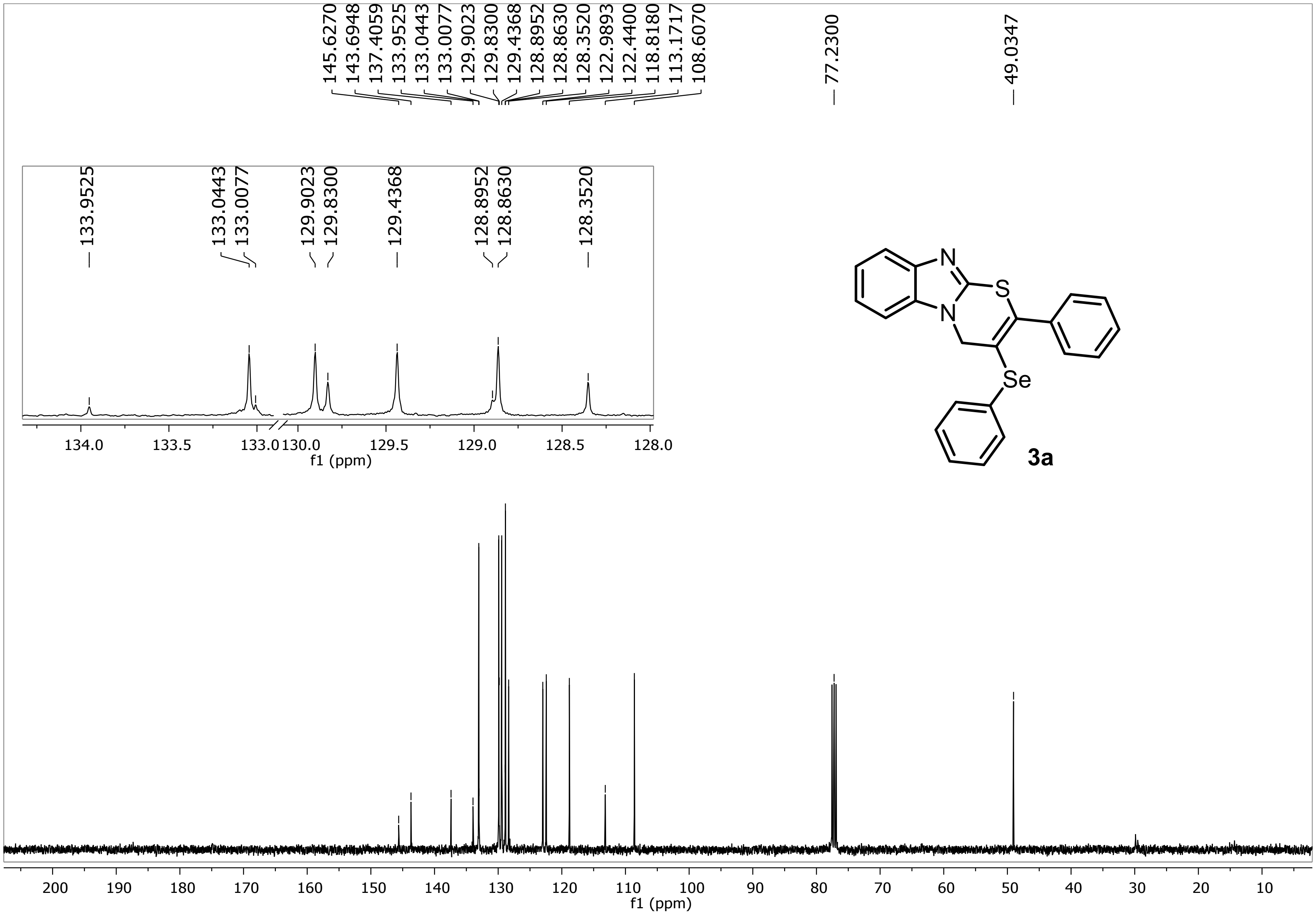

Figure S18: $^{13}C\{^1H\}$ NMR (100 MHz, $CDCl_3$) spectrum of compound **3a**.

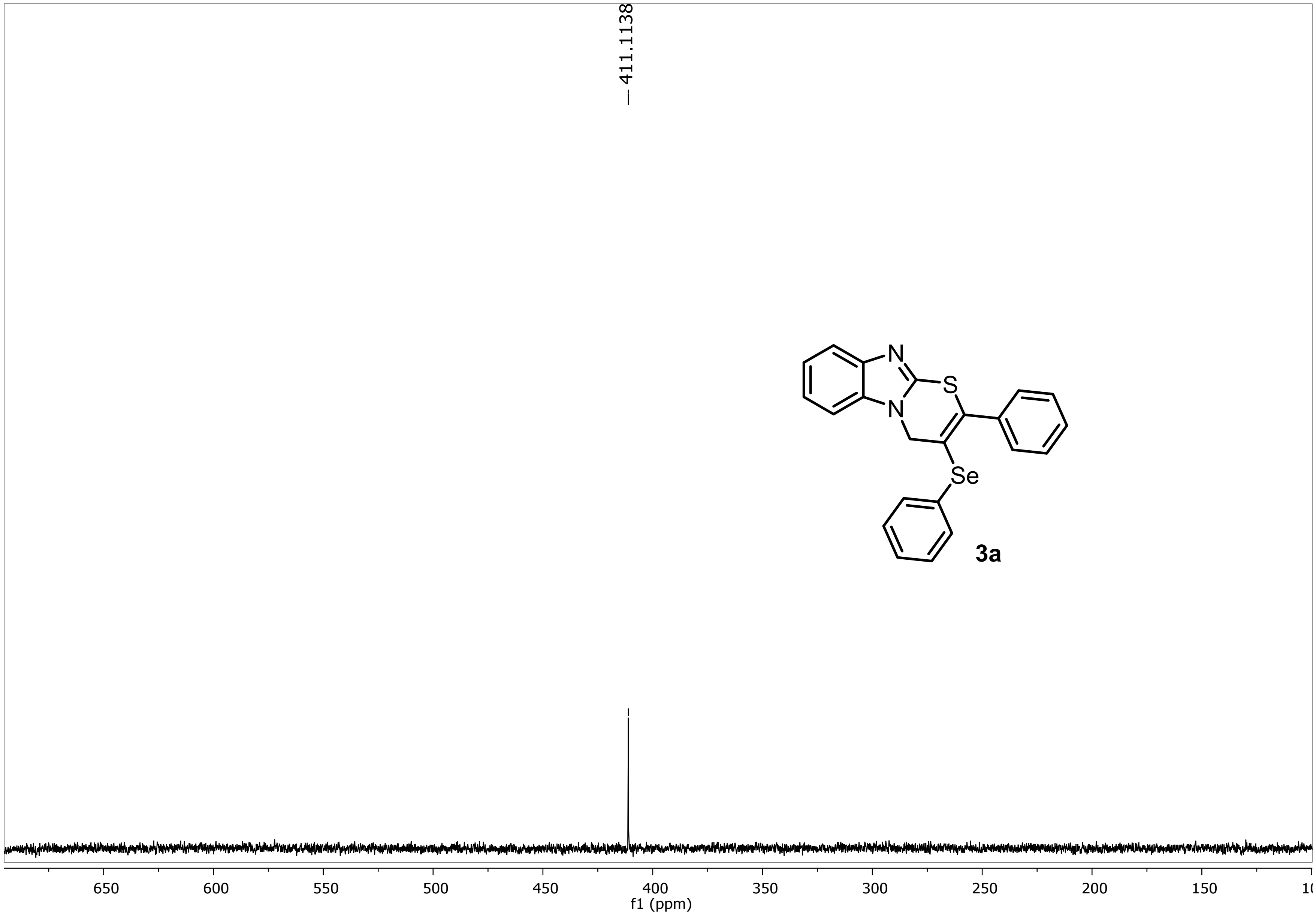

Figure S19: $^{77}Se\{^{1}H\}$ NMR (76 MHz, $CDCl_3$) spectrum of compound **3a**.

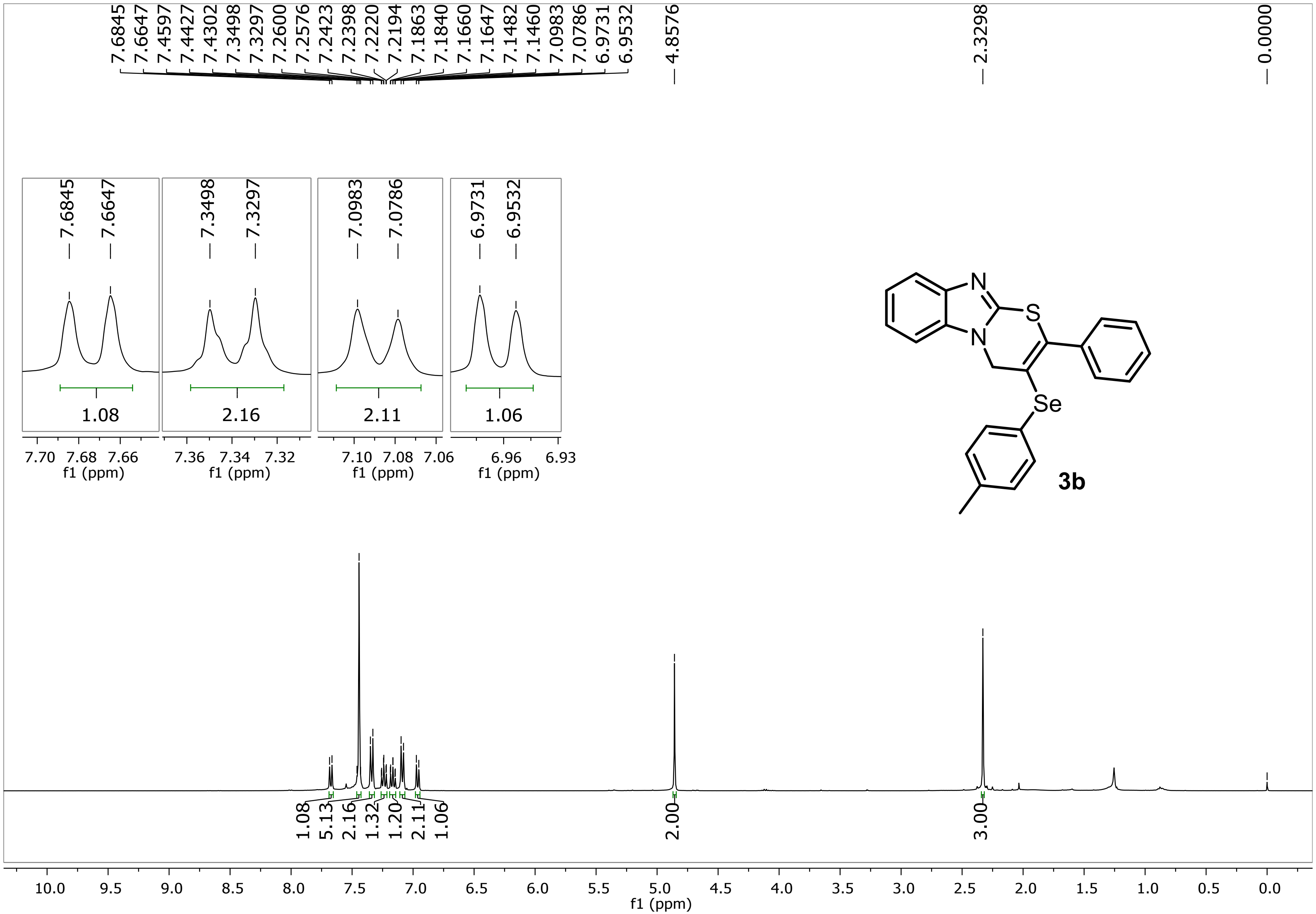

Figure S20: ^{1}H NMR (400 MHz, $CDCl_3$) spectrum of compound **3b**.

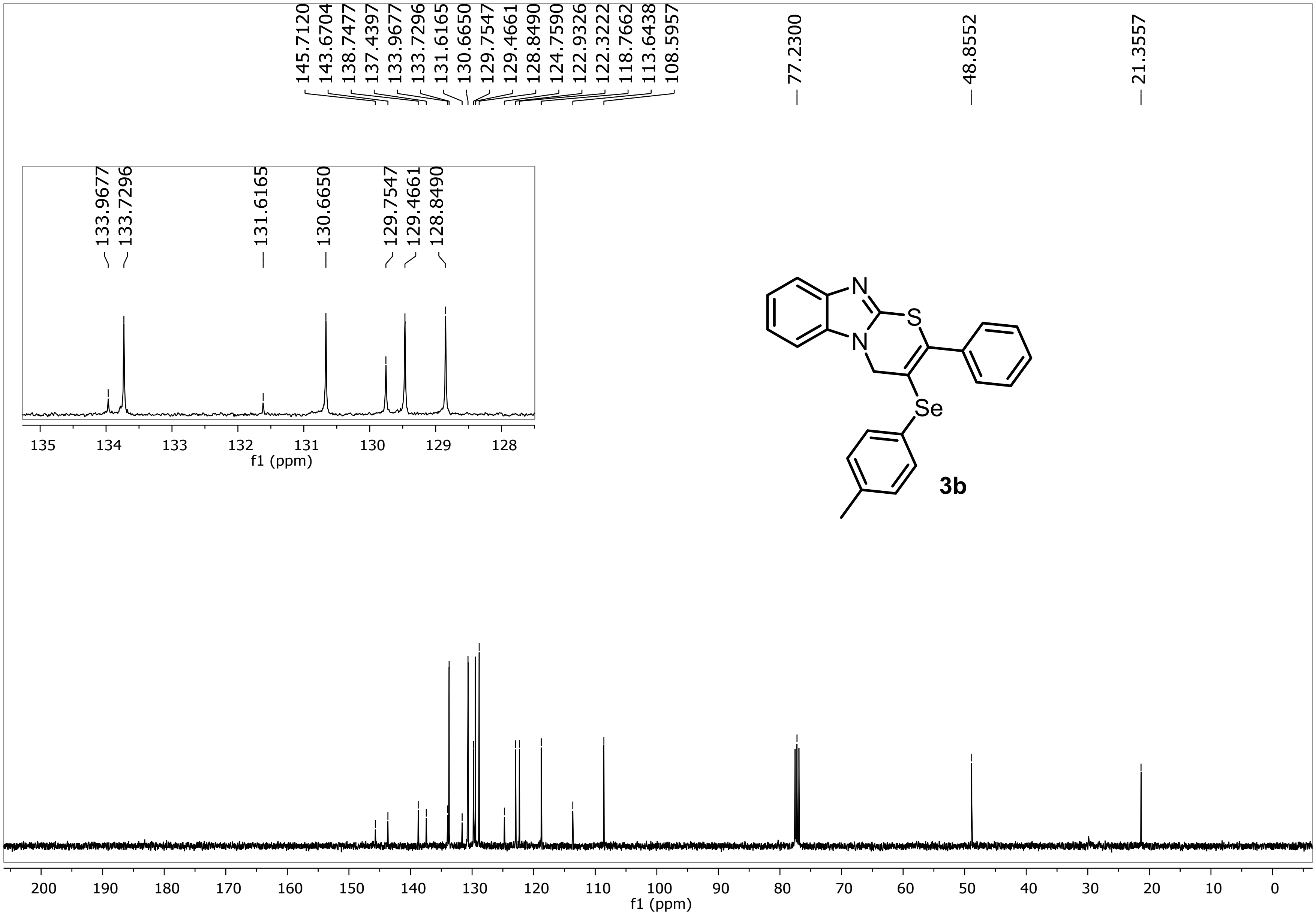

3b

Figure S21: $^{13}C\{^1H\}$ NMR (100 MHz, $CDCl_3$) spectrum of compound **3b**.

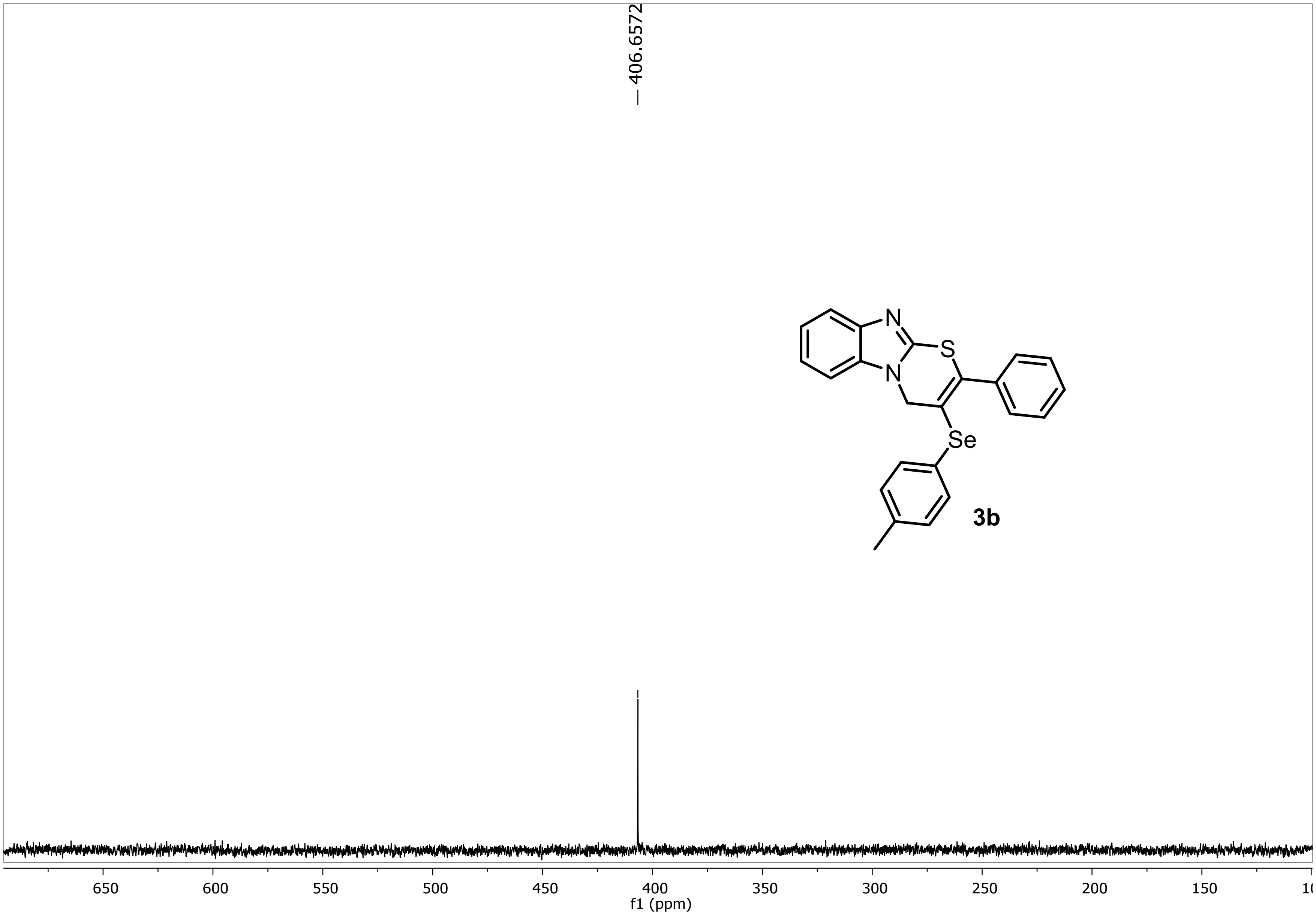

Figure S22: ^{77}Se{^{1}H} NMR (76 MHz, $CDCl_3$) spectrum of compound **3b**.

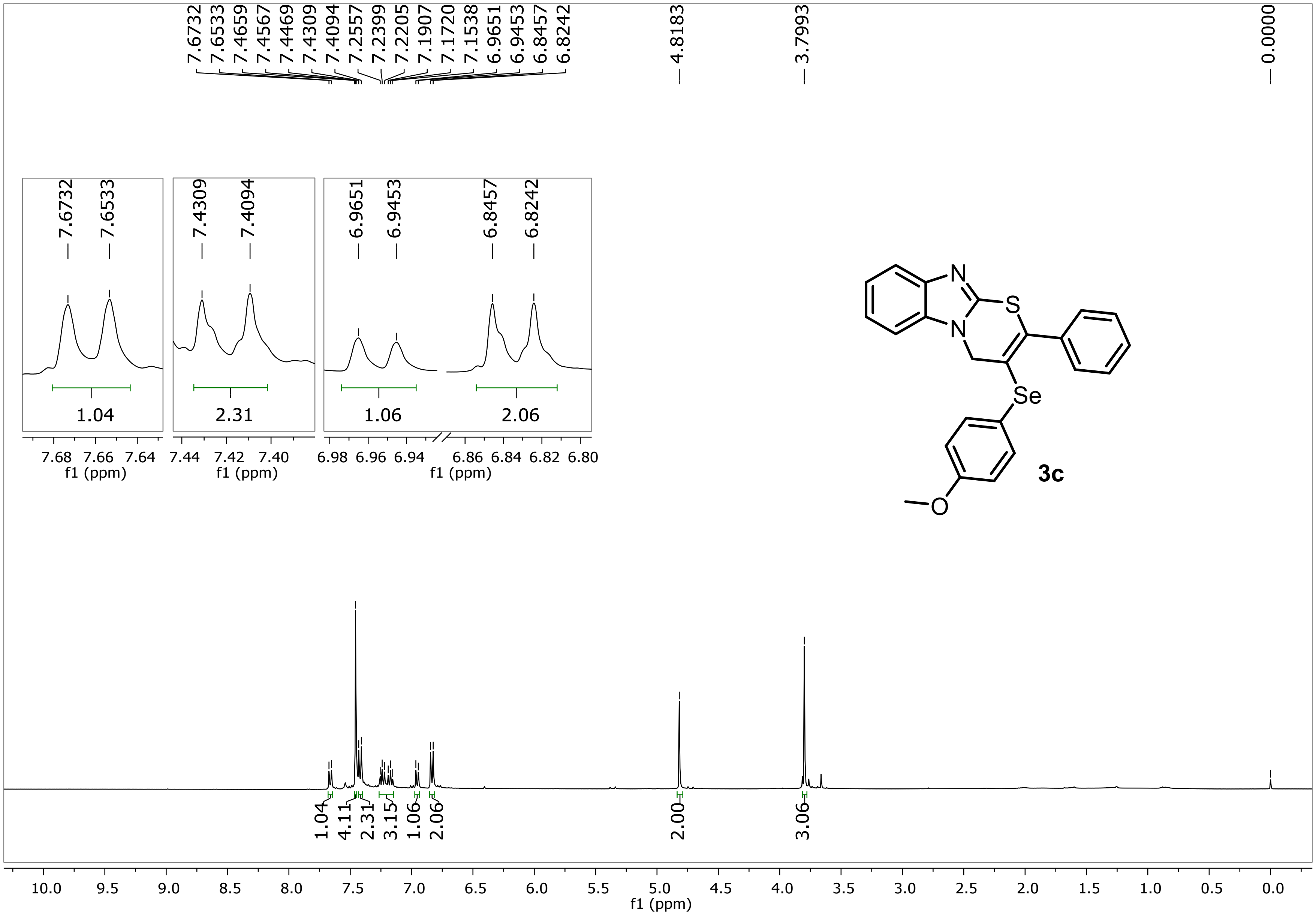

Figure S23: ^{1}H NMR (400 MHz, $CDCl_3$) spectrum of compound **3c**.

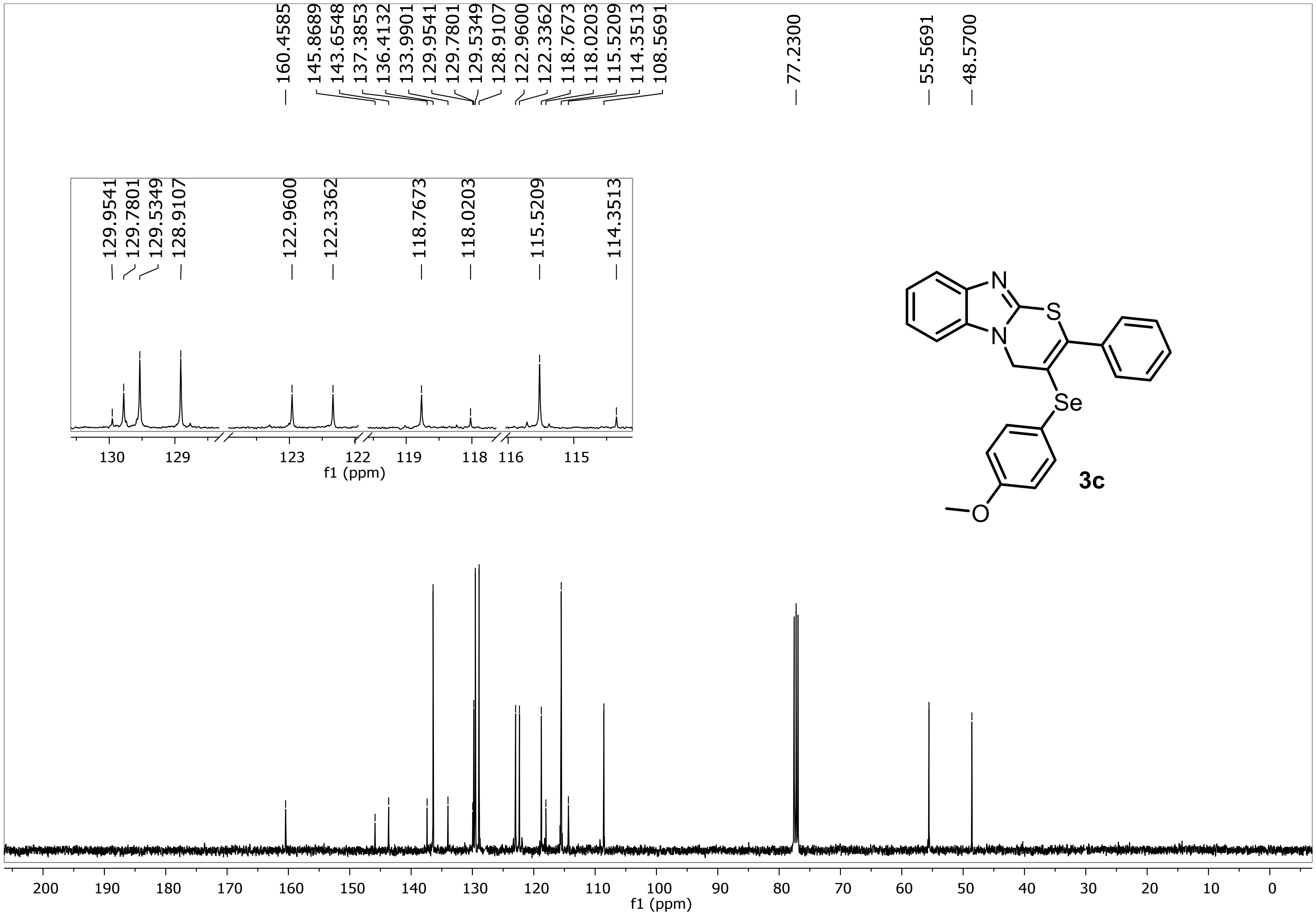

Figure S24: $^{13}C\{^{1}H\}$ NMR (100 MHz, $CDCl_3$) spectrum of compound **3c**.

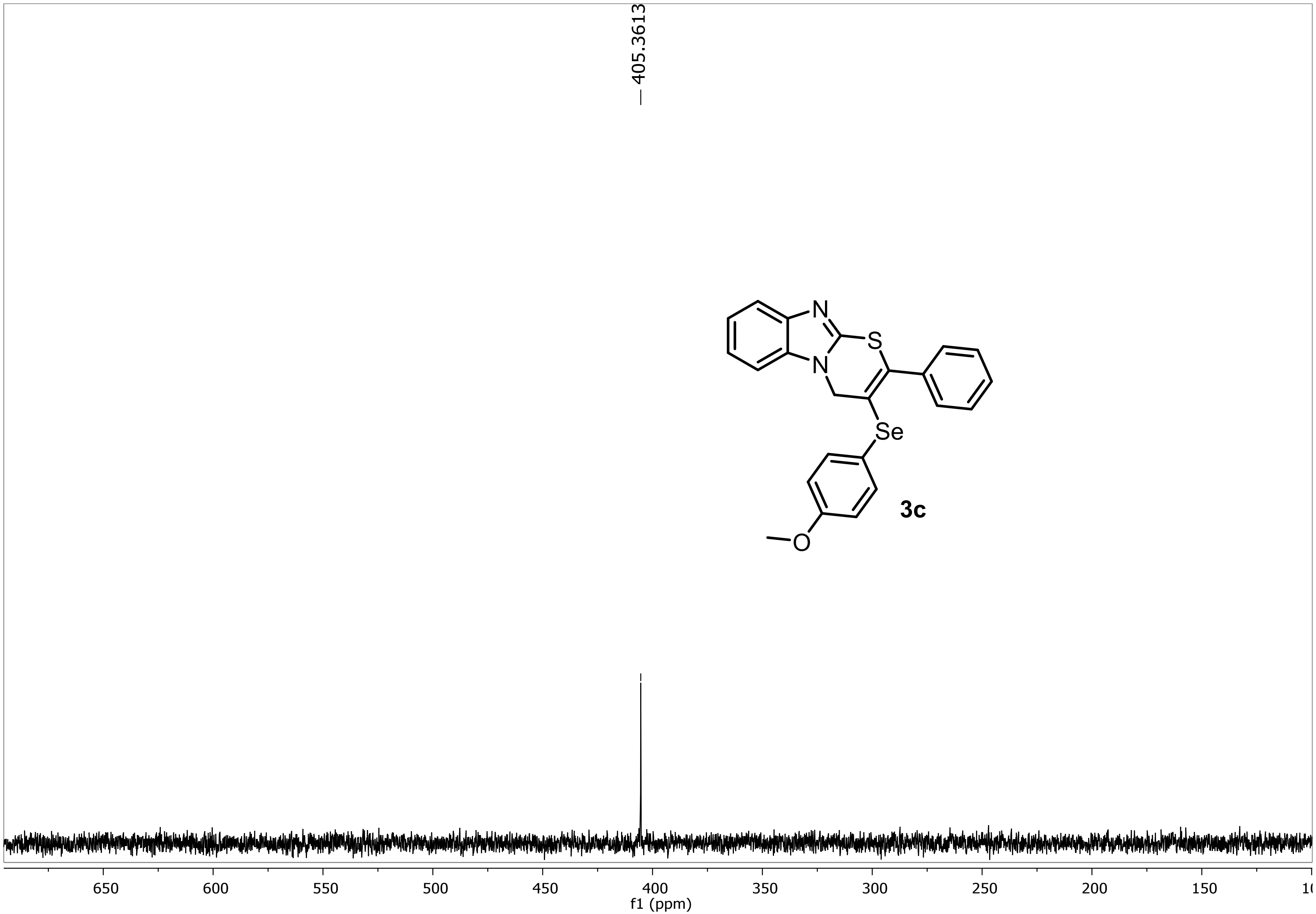

Figure S25: $^{77}Se\{^1H\}$ NMR (76 MHz, $CDCl_3$) spectrum of compound **3c**.

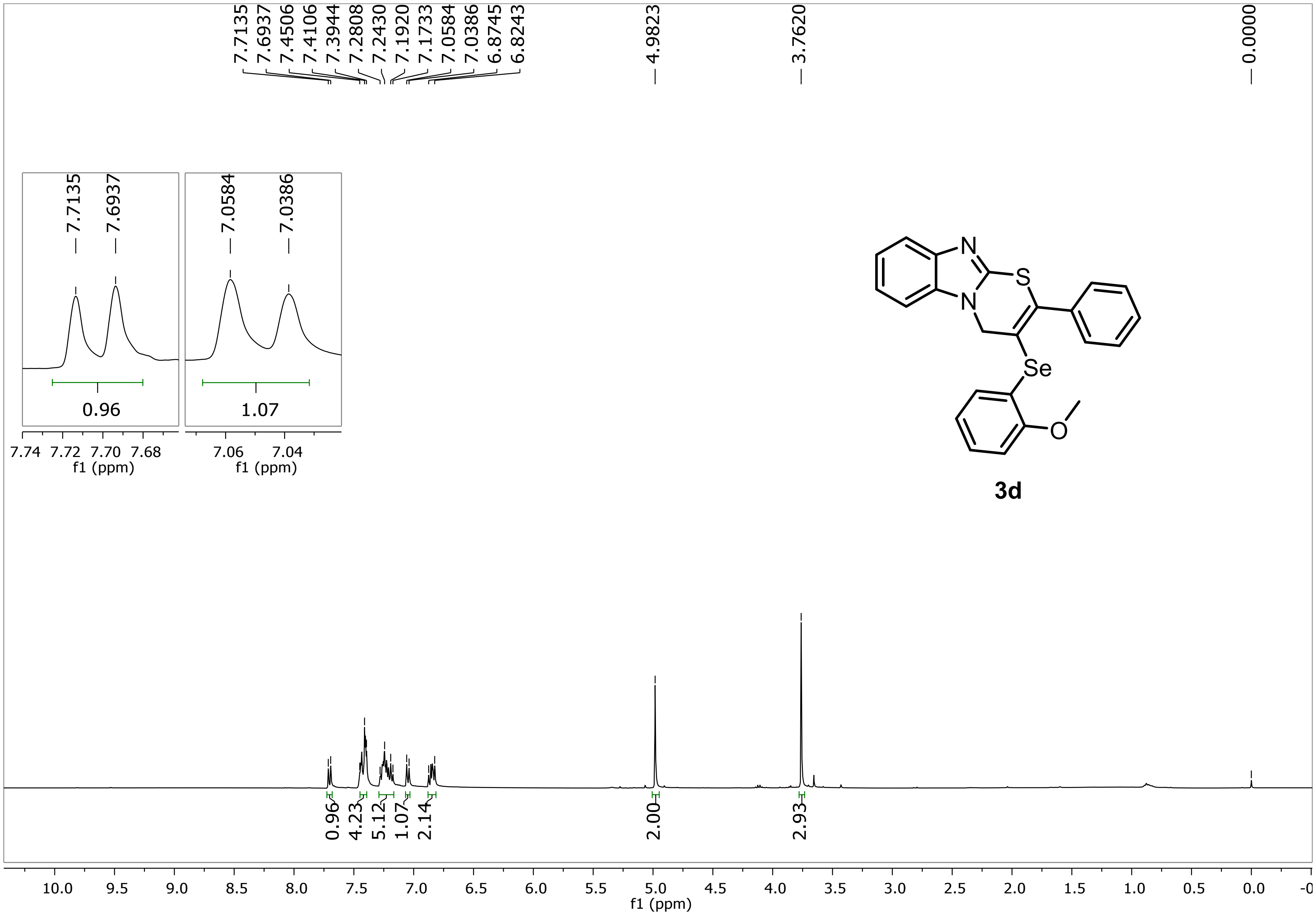

3d

Figure S26: ^{1}H NMR (400 MHz, $CDCl_3$) spectrum of compound **3d**.

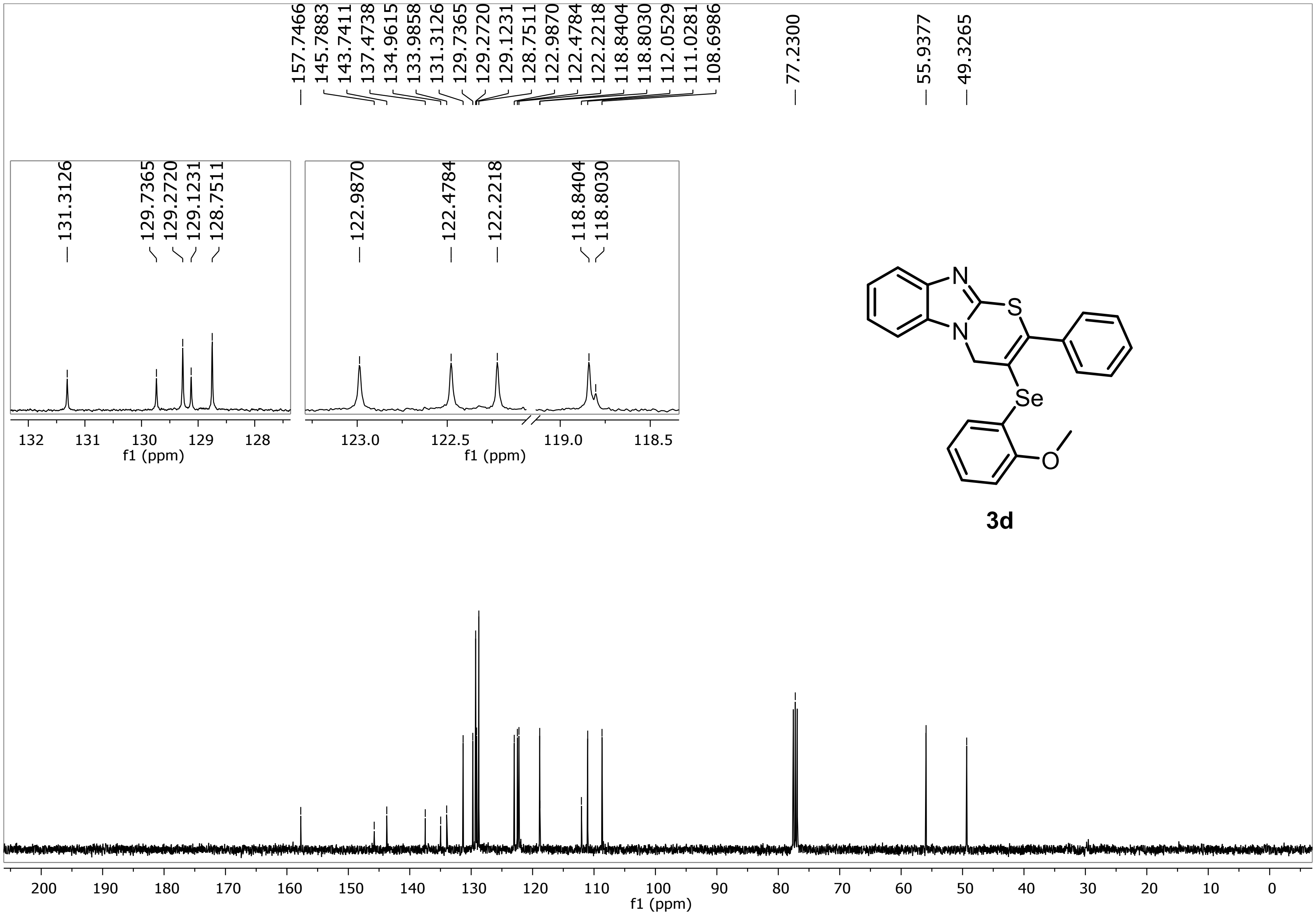

Figure S27: $^{13}C\{^{1}H\}$ NMR (100 MHz, $CDCl_3$) spectrum of compound **3d**.

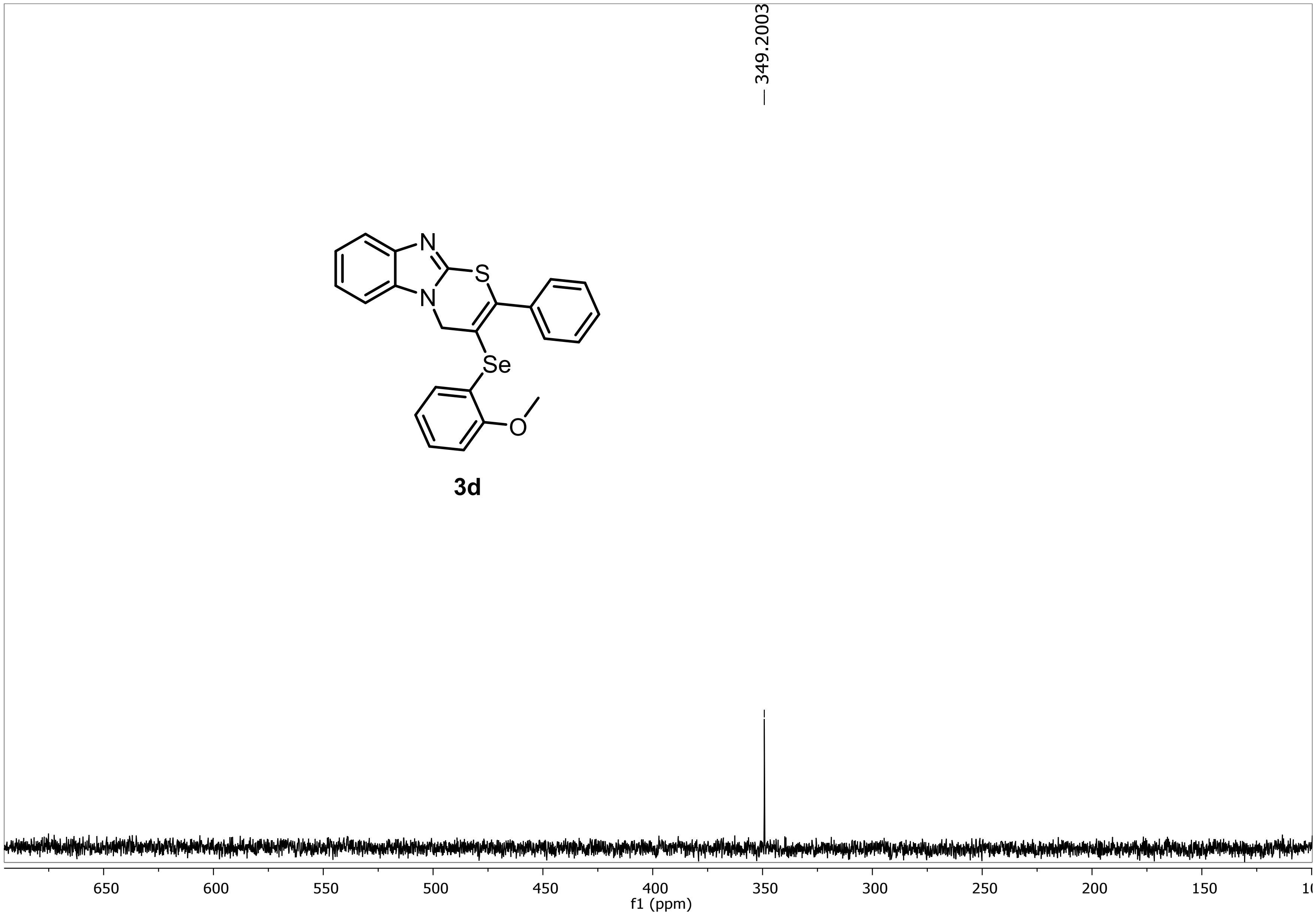

Figure S28: ^{77}Se{^{1}H} NMR (76 MHz, $CDCl_3$) spectrum of compound **3d**.

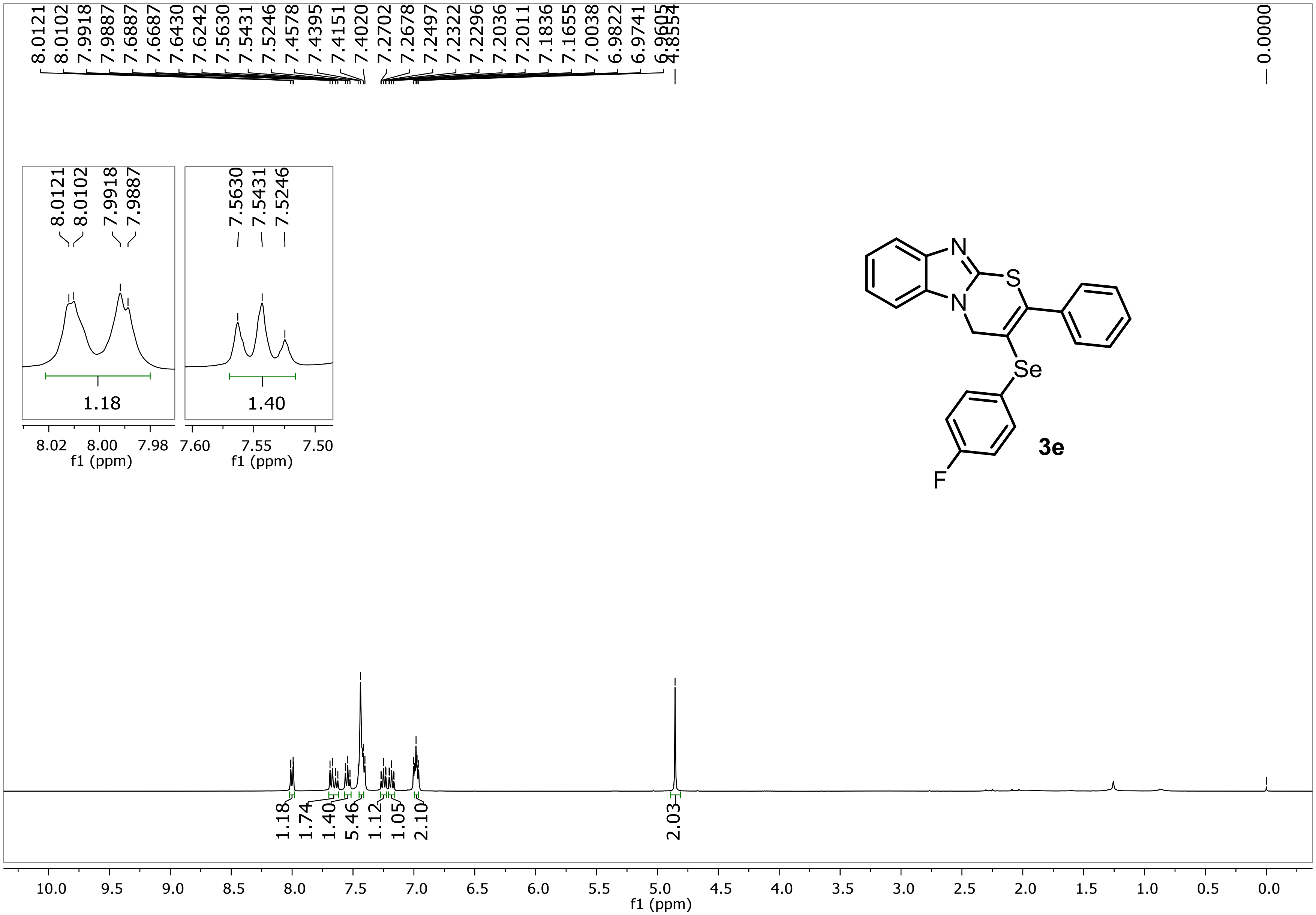

Figure S29: ^{1}H NMR (400 MHz, $CDCl_3$) spectrum of compound **3e**.

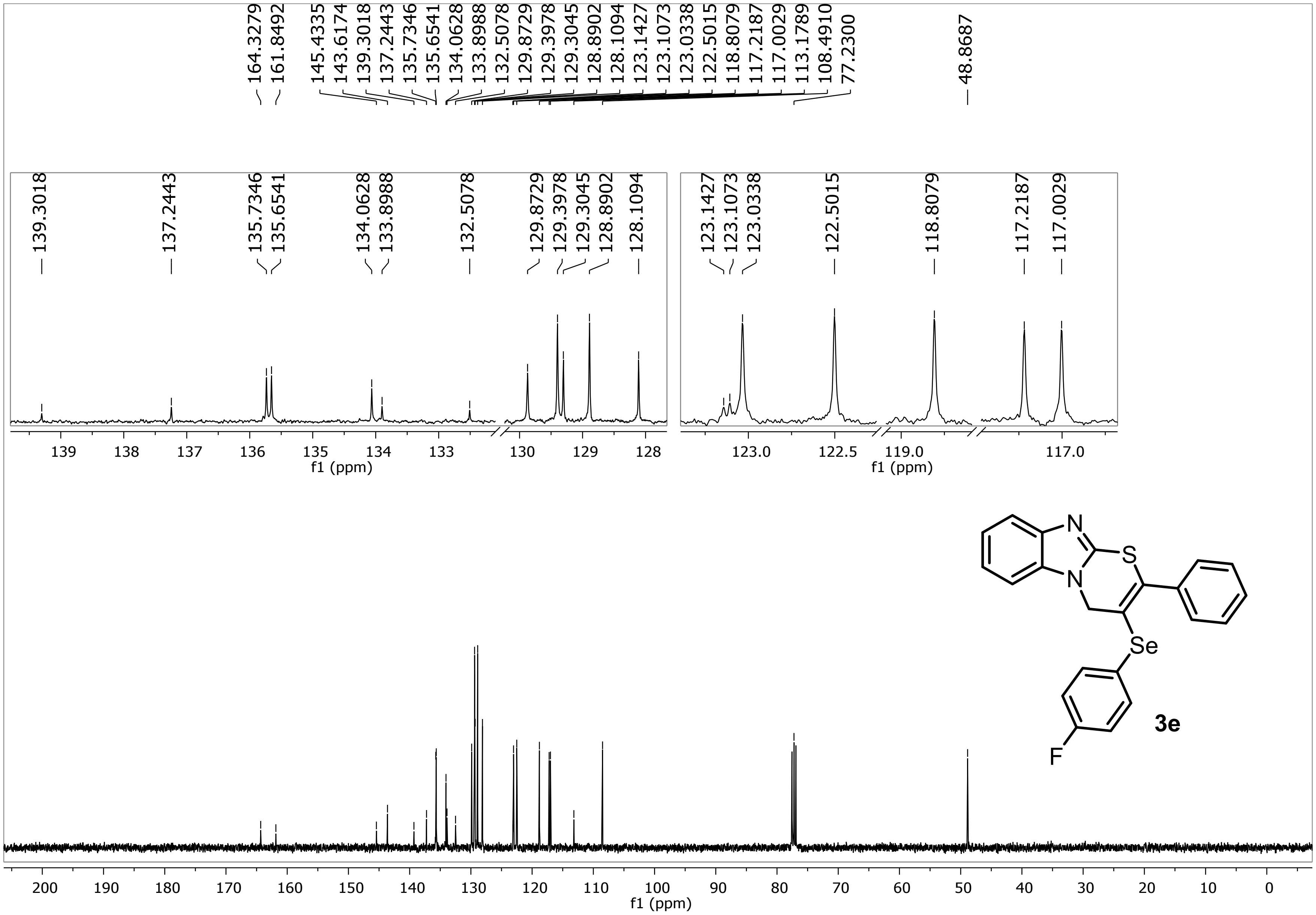

Figure S30: $^{13}C\{^{1}H\}$ NMR (100 MHz, $CDCl_3$) spectrum of compound **3e**.

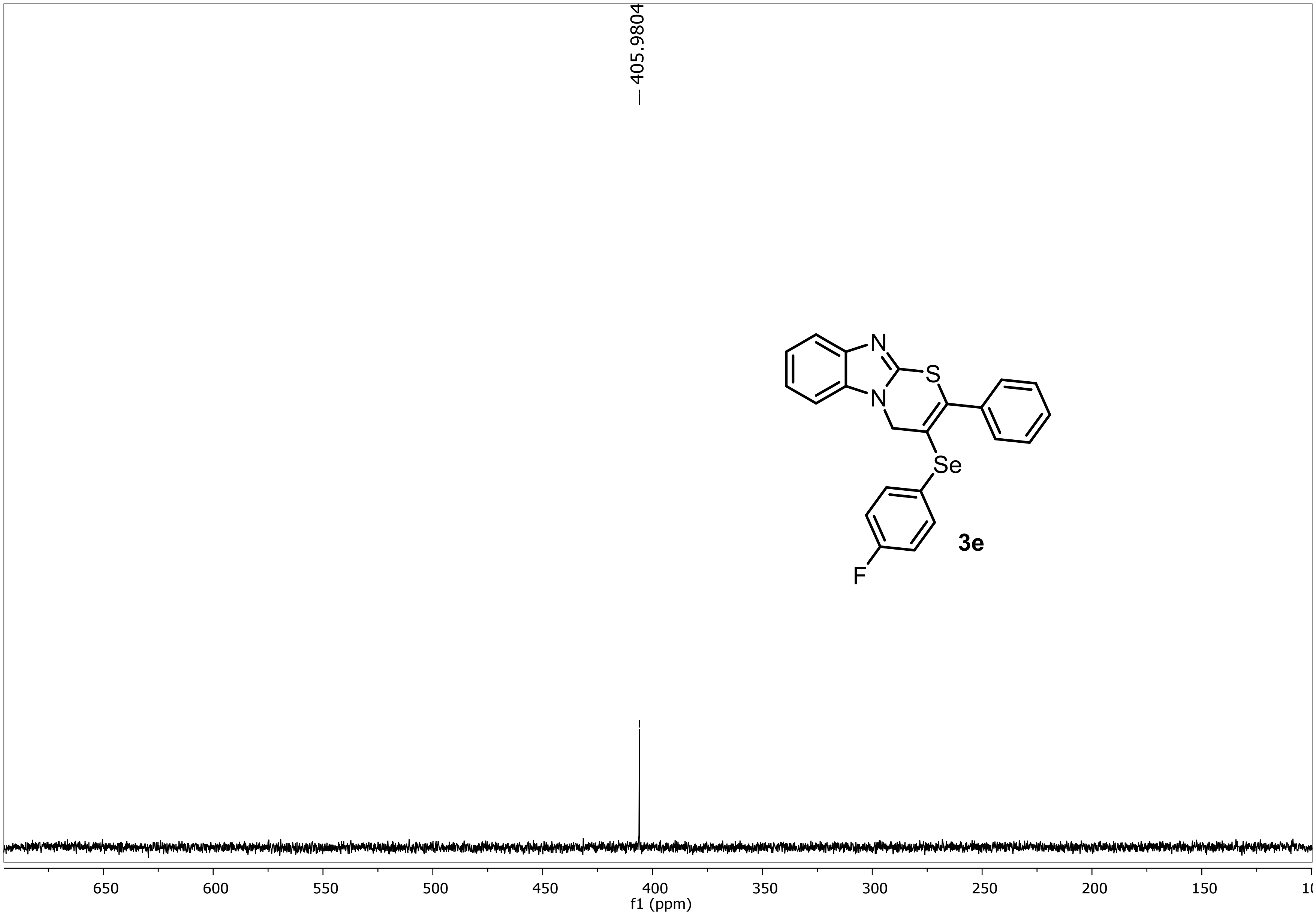

Figure S31: $^{77}Se\{^{1}H\}$ NMR (76 MHz, $CDCl_3$) spectrum of compound **3e**.

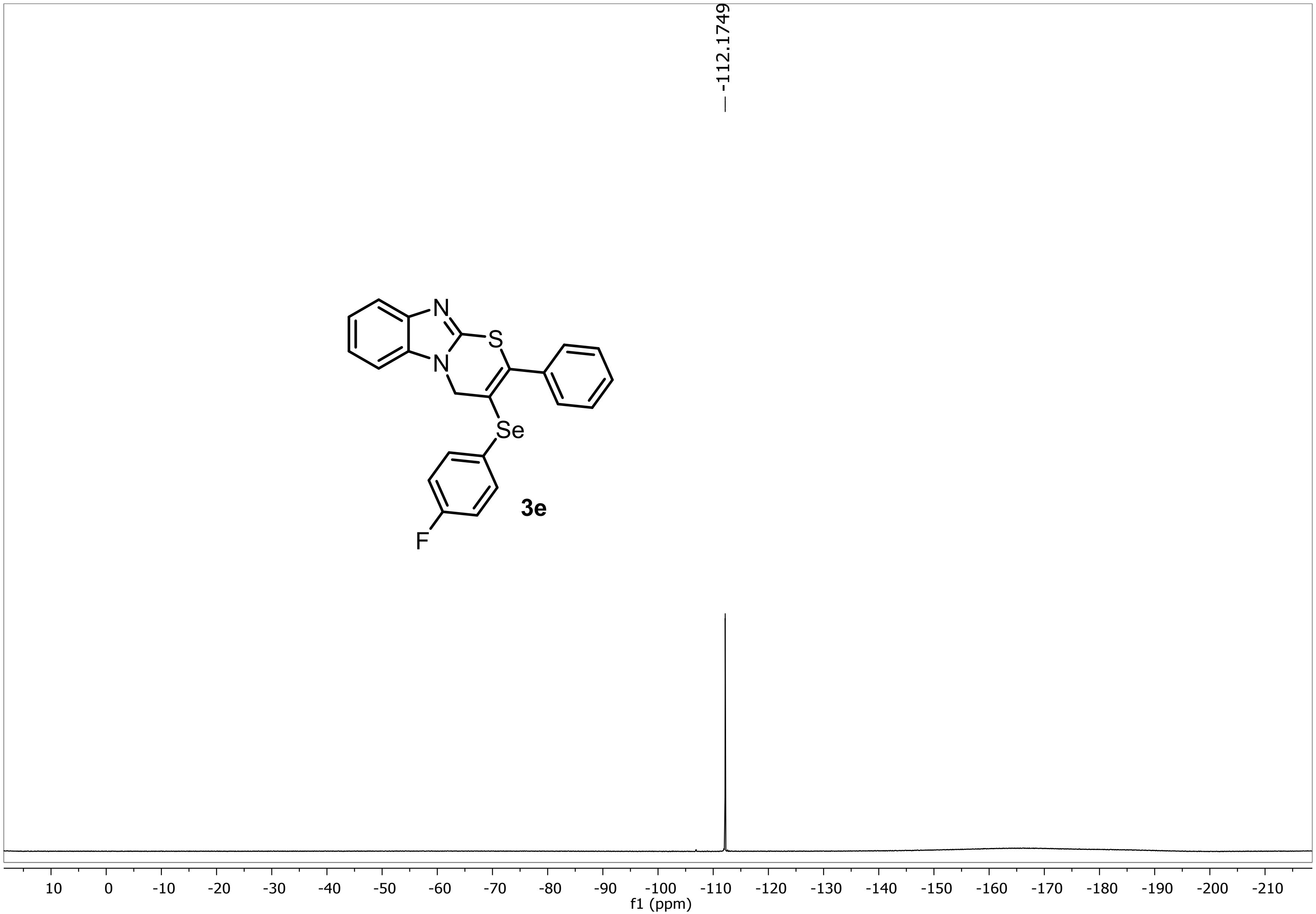

Figure S32: $^{19}F\{^{1}H\}$ NMR (376 MHz, $CDCl_3$) spectrum of compound **3e**.

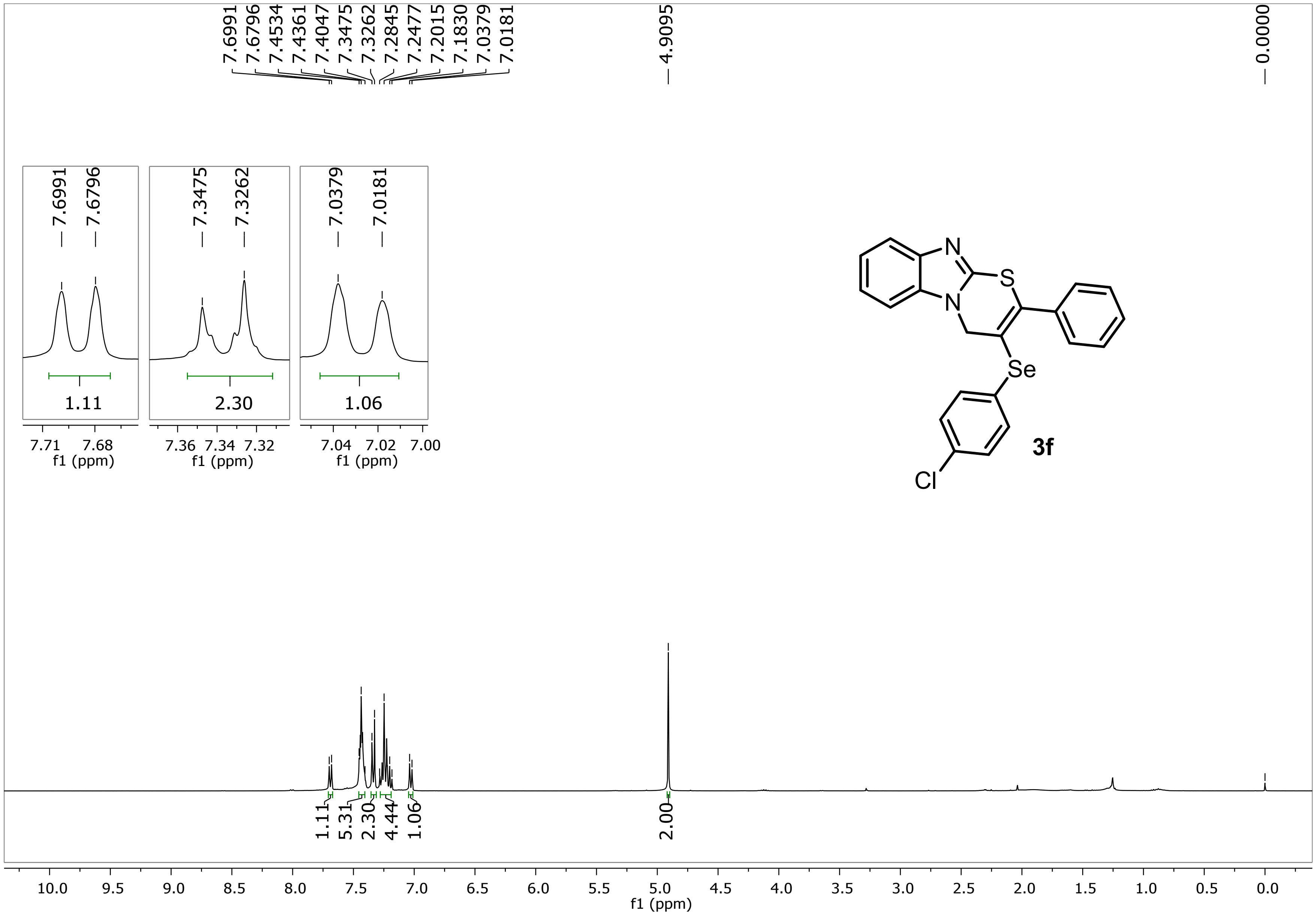

Figure S33: ^{1}H NMR (400 MHz, $CDCl_3$) spectrum of compound **3f**.

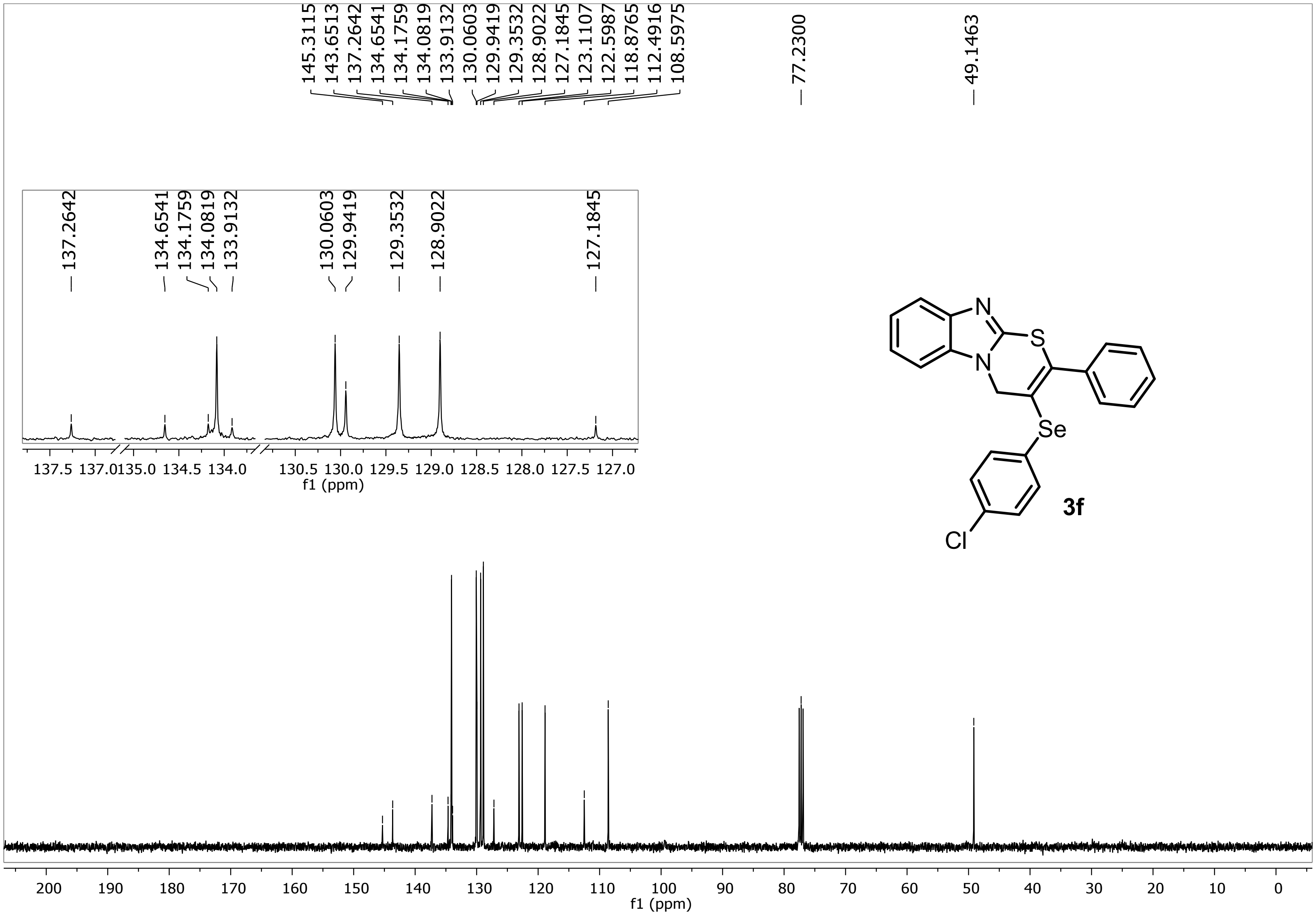

Figure S34: $^{13}C\{^{1}H\}$ NMR (100 MHz, $CDCl_3$) spectrum of compound **3f**.

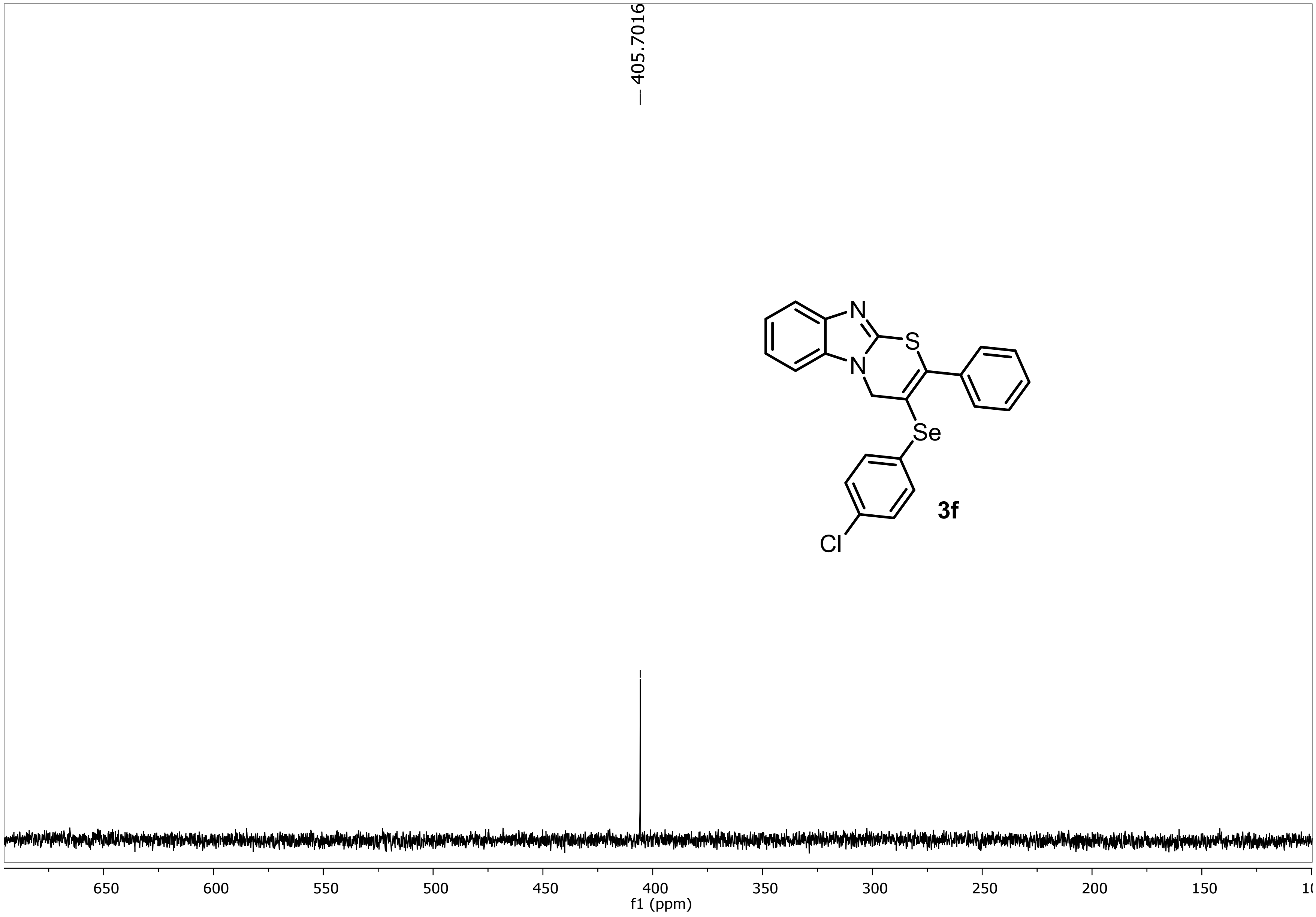

Figure S35: $^{77}Se\{^{1}H\}$ NMR (76 MHz, $CDCl_3$) spectrum of compound **3f**.

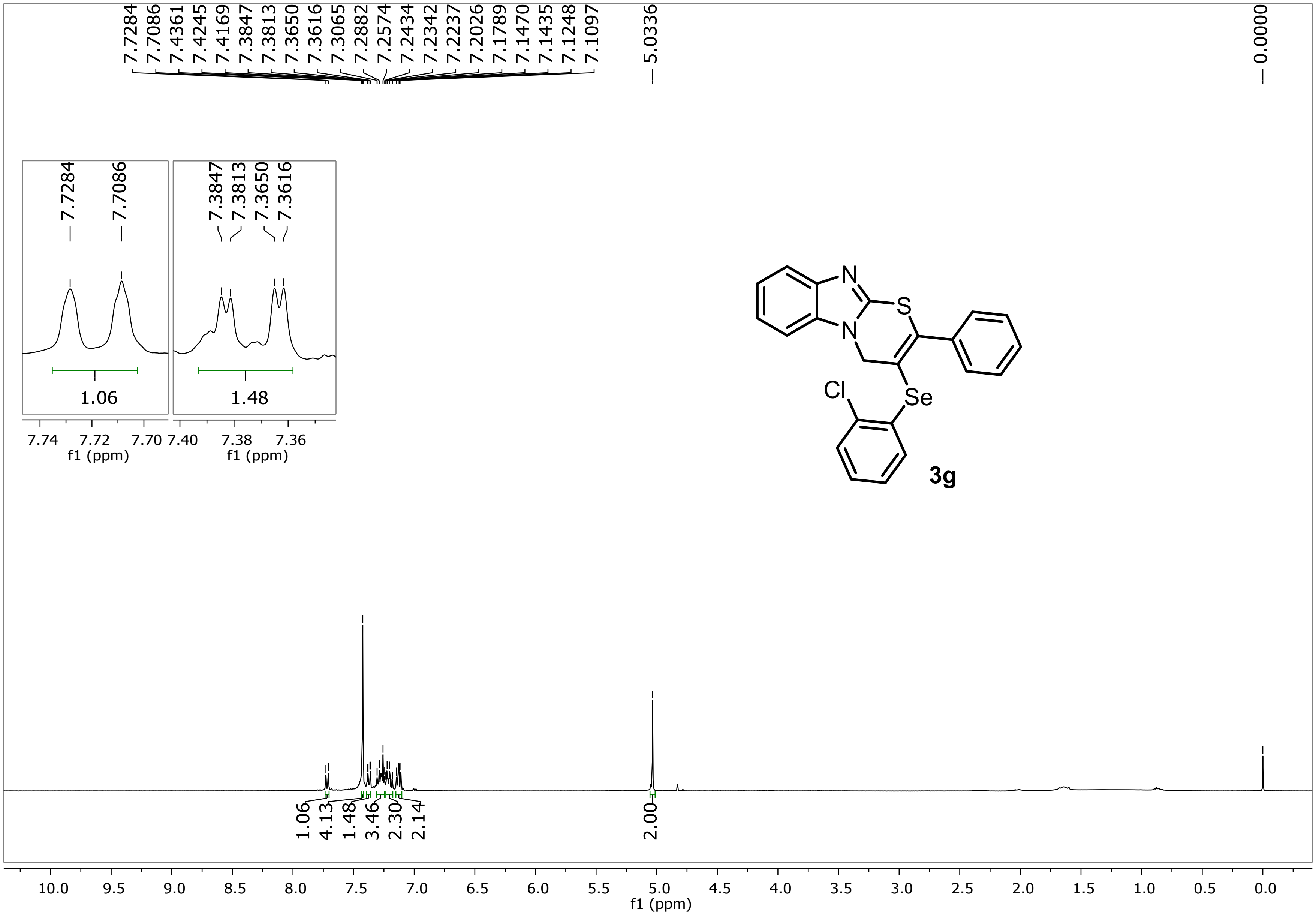

Figure S36: ^{1}H NMR (400 MHz, $CDCl_3$) spectrum of compound **3g**.

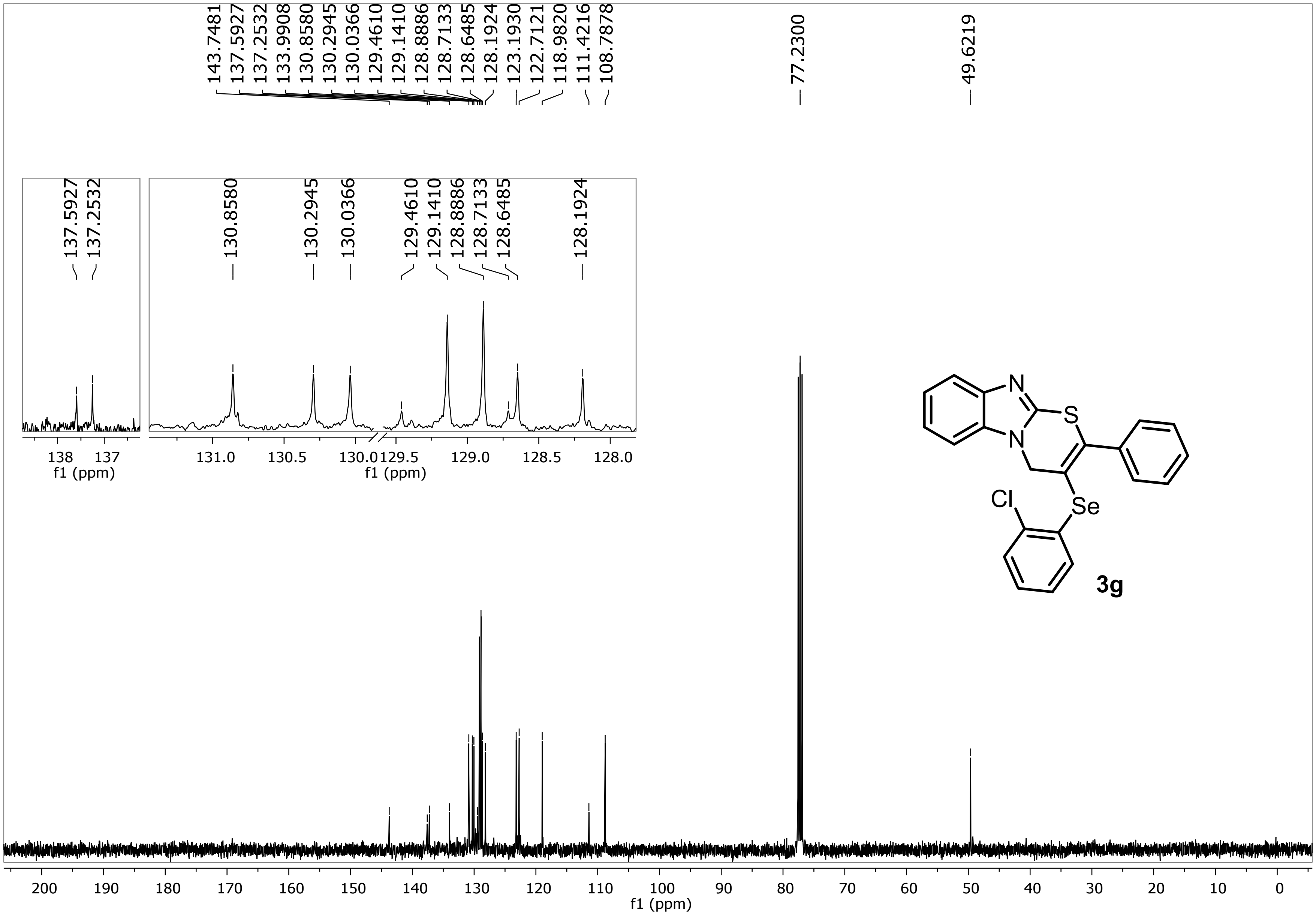

Figure S37: $^{13}C\{^{1}H\}$ NMR (100 MHz, $CDCl_3$) spectrum of compound **3g**.

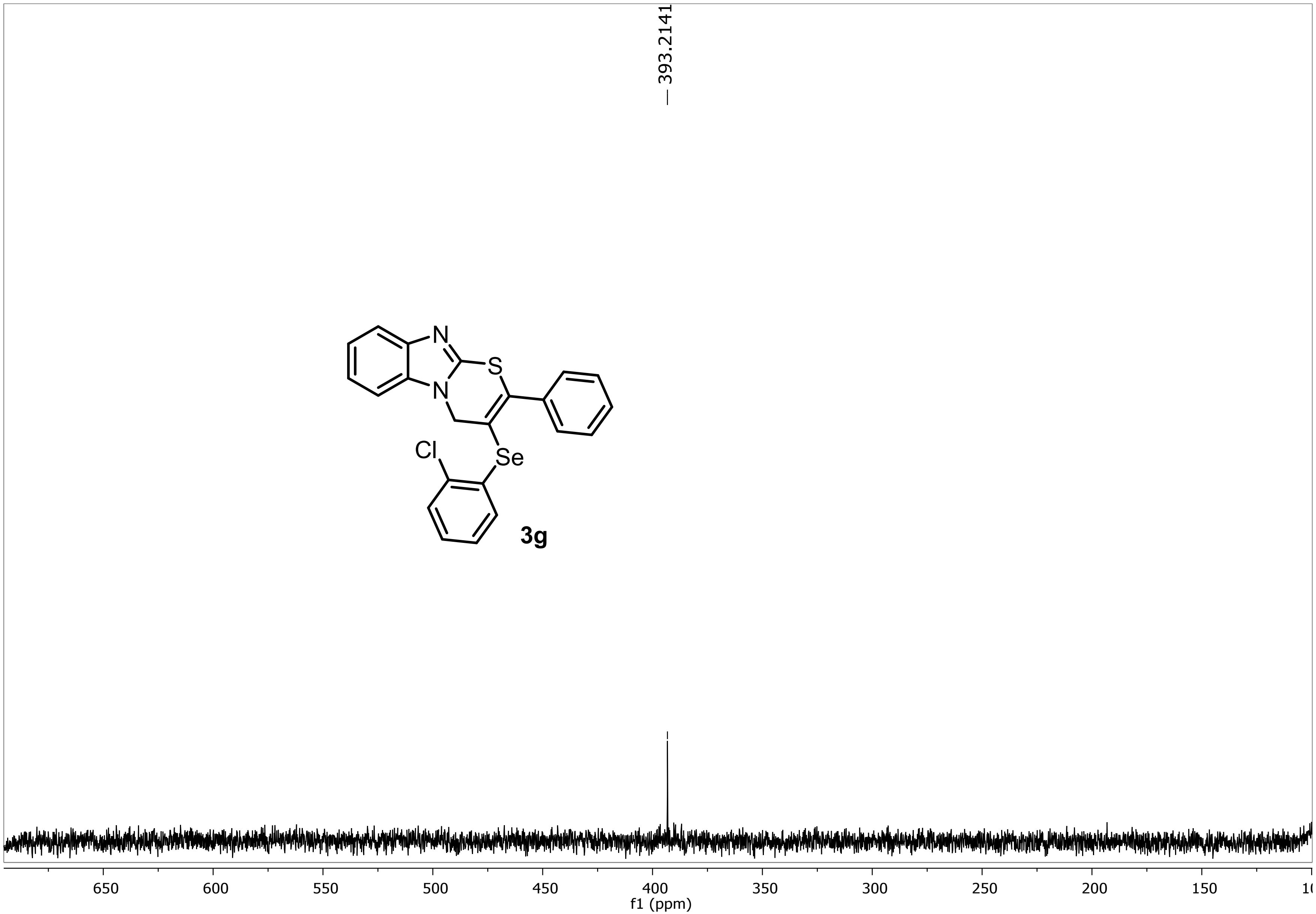

Figure S38: $^{77}Se\{^{1}H\}$ NMR (76 MHz, $CDCl_3$) spectrum of compound **3g**.

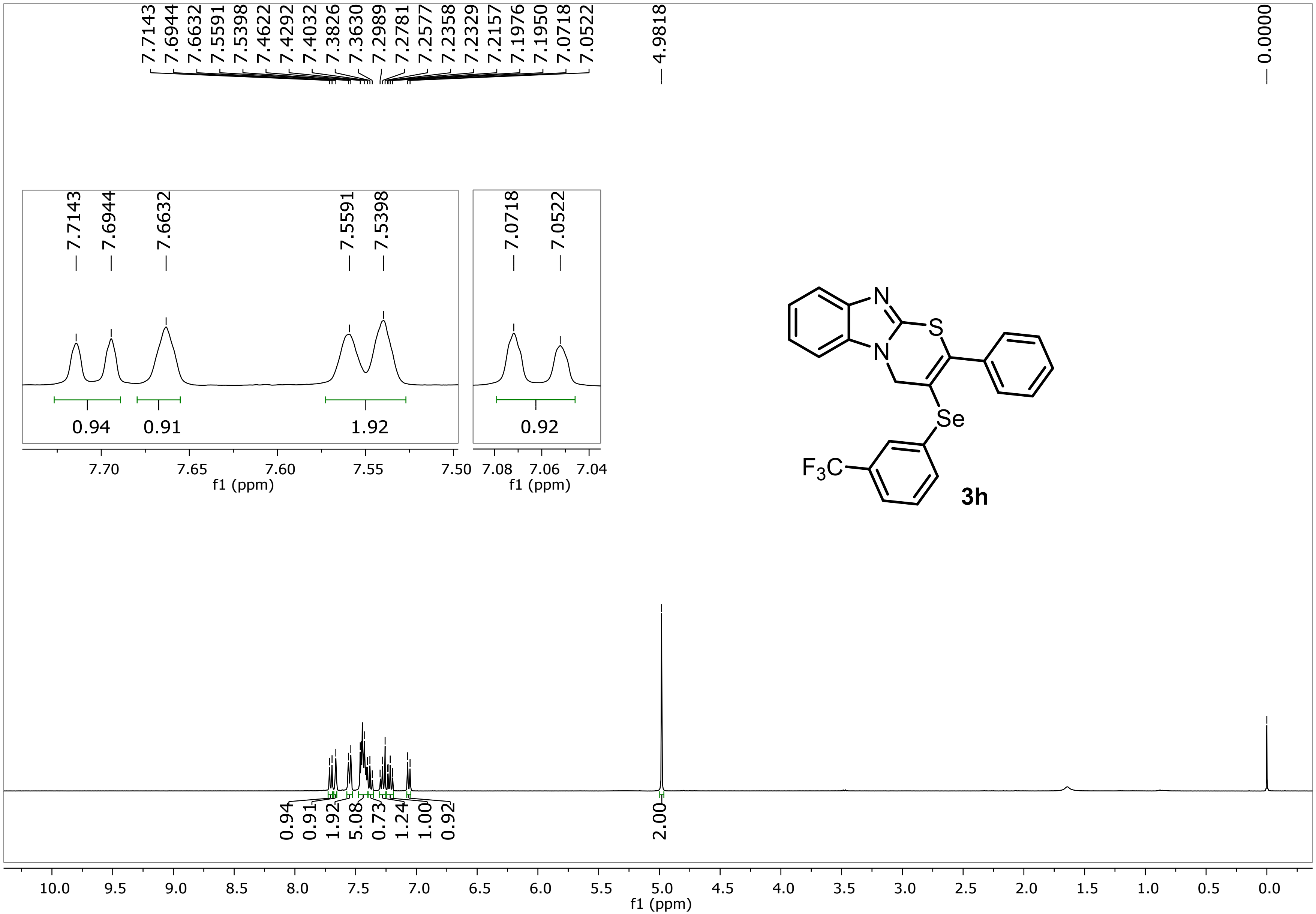

Figure S39: ^{1}H NMR (400 MHz, $CDCl_3$) spectrum of compound **3h**.

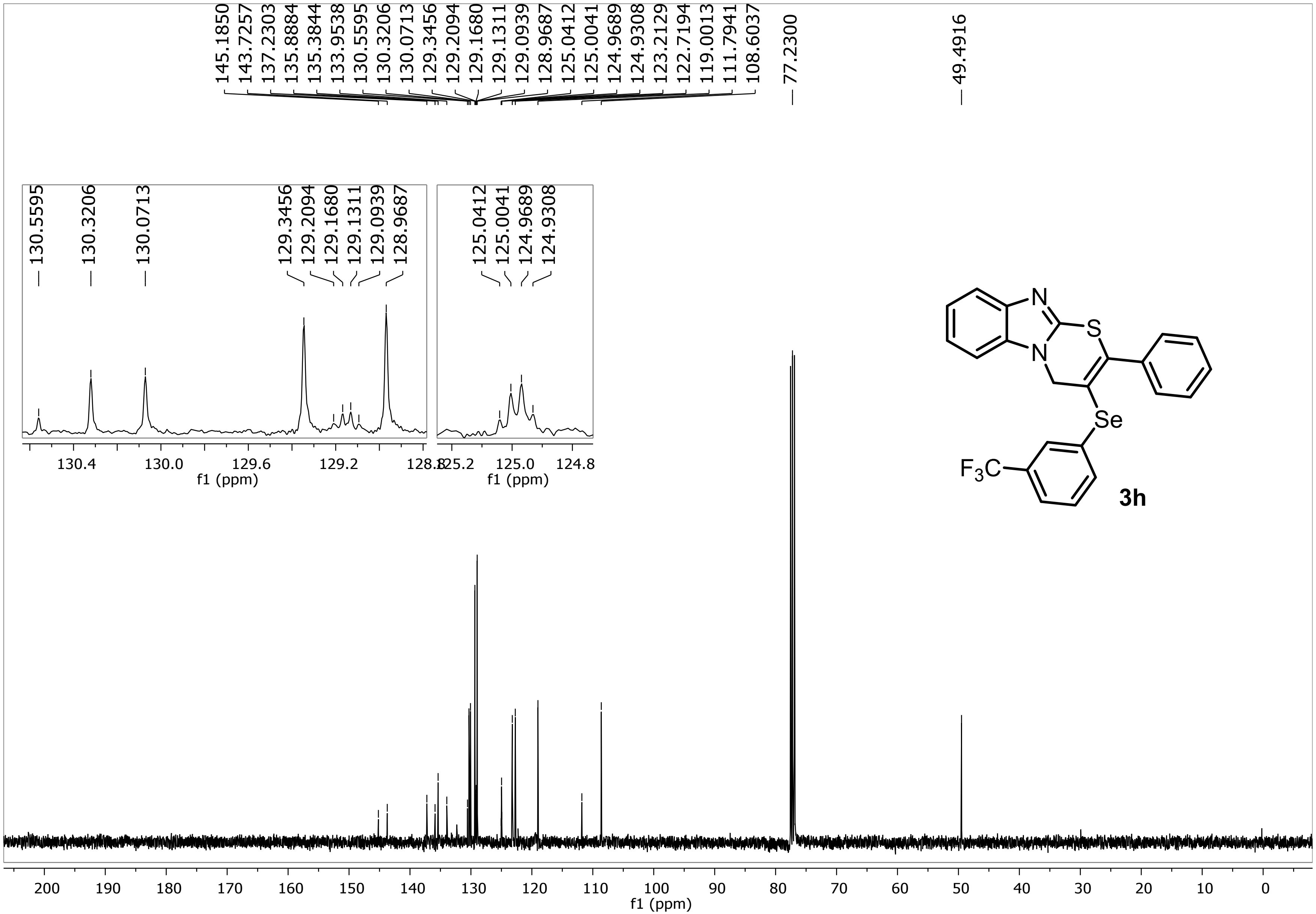

Figure S40: $^{13}C\{^1H\}$ NMR (100 MHz, $CDCl_3$) spectrum of compound **3h**.

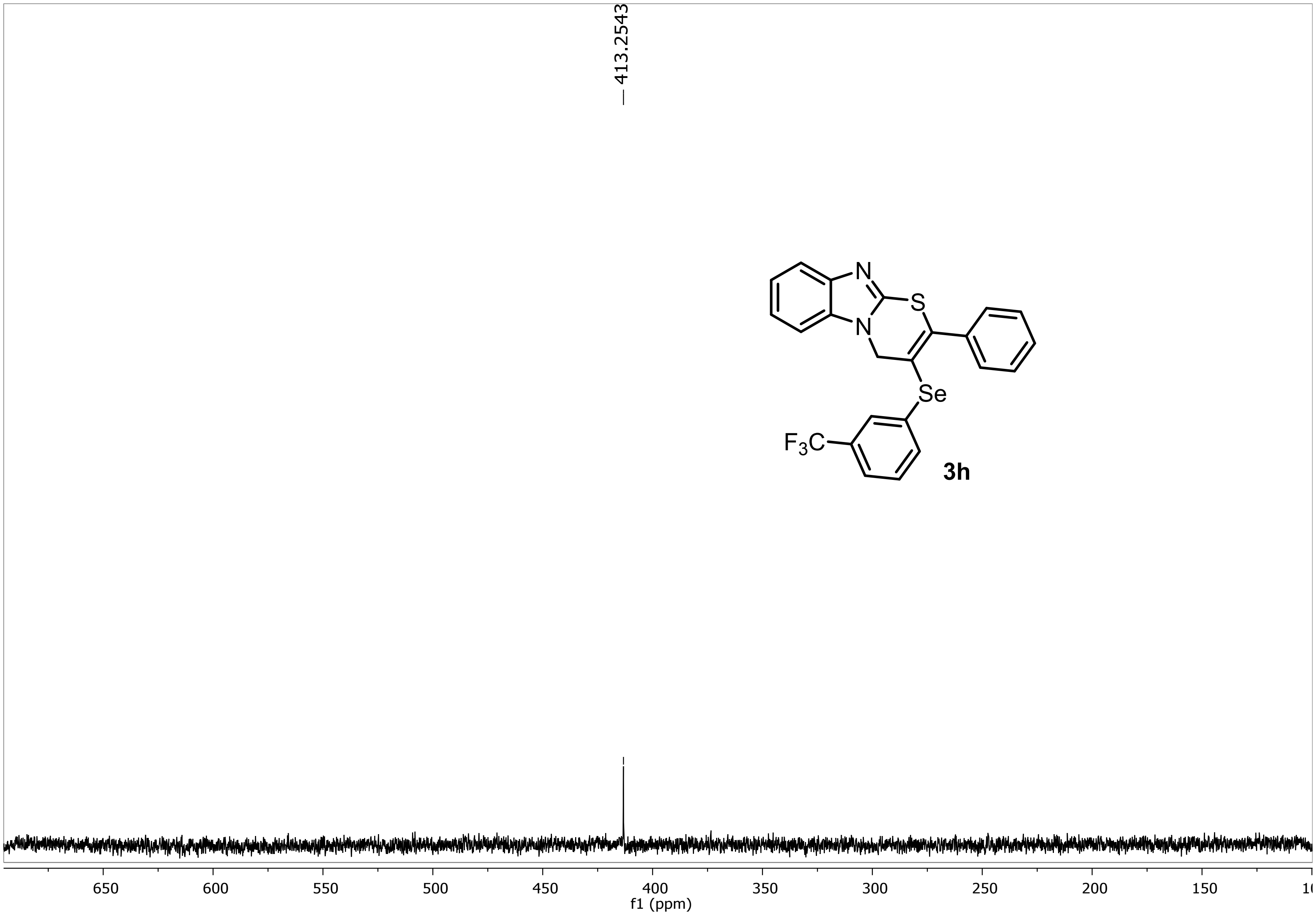

Figure S41: $^{77}Se\{^{1}H\}$ NMR (76 MHz, $CDCl_3$) spectrum of compound **3h**.

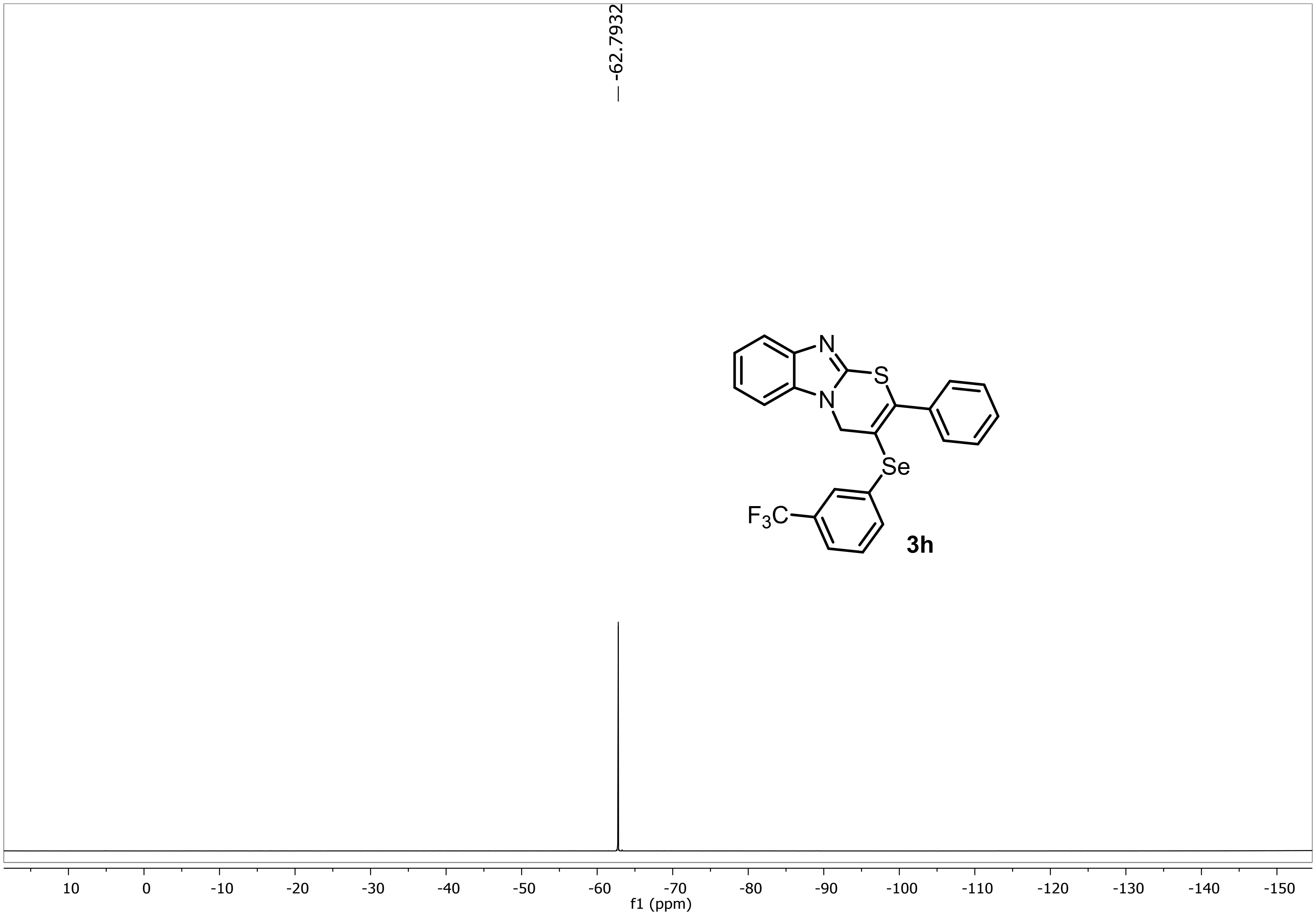

Figure S42: $^{19}F\{^{1}H\}$ NMR (376 MHz, $CDCl_3$) spectrum of compound **3h**.

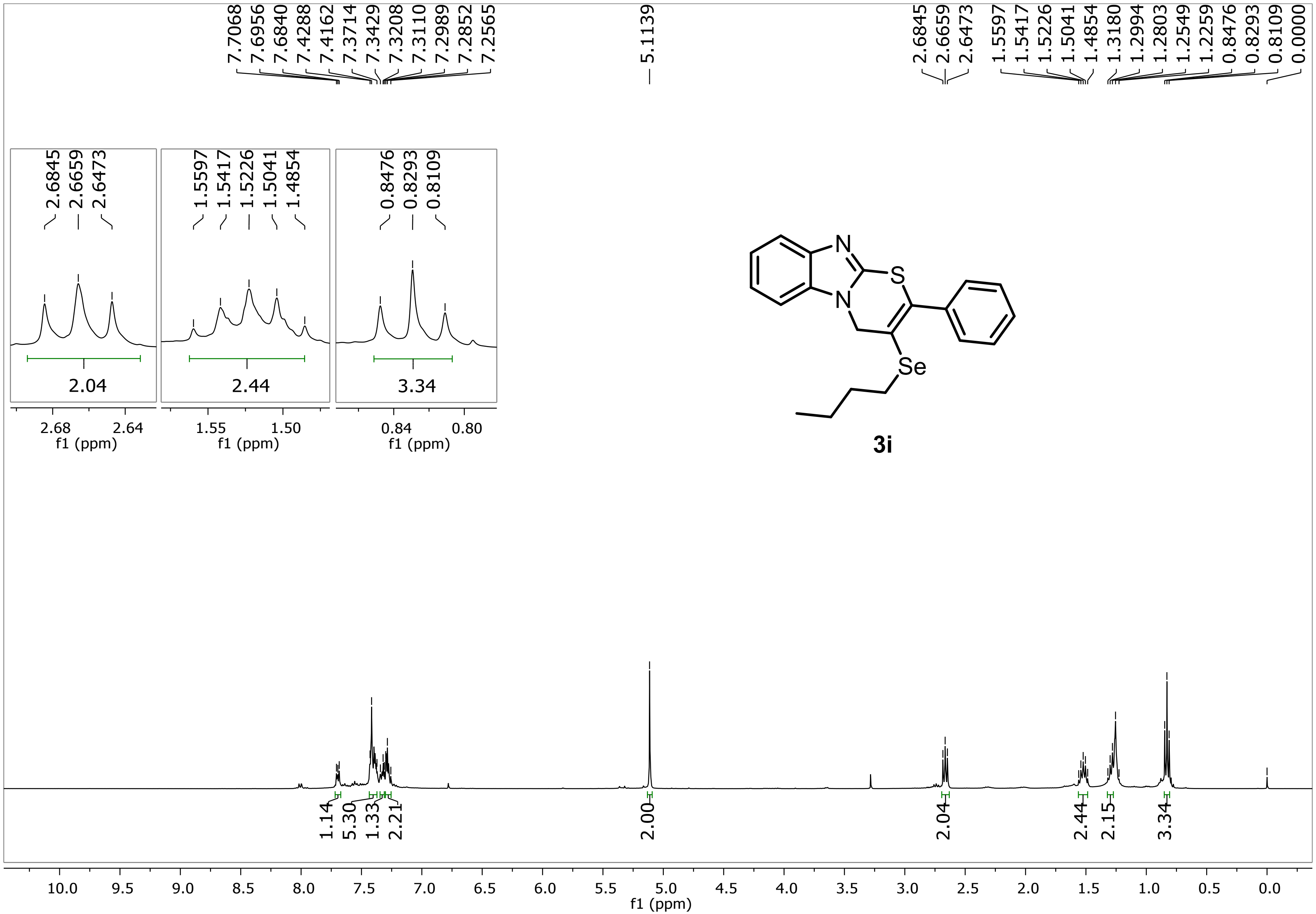

3i

Figure S43: ^{1}H NMR (400 MHz, $CDCl_3$) spectrum of compound **3i**.

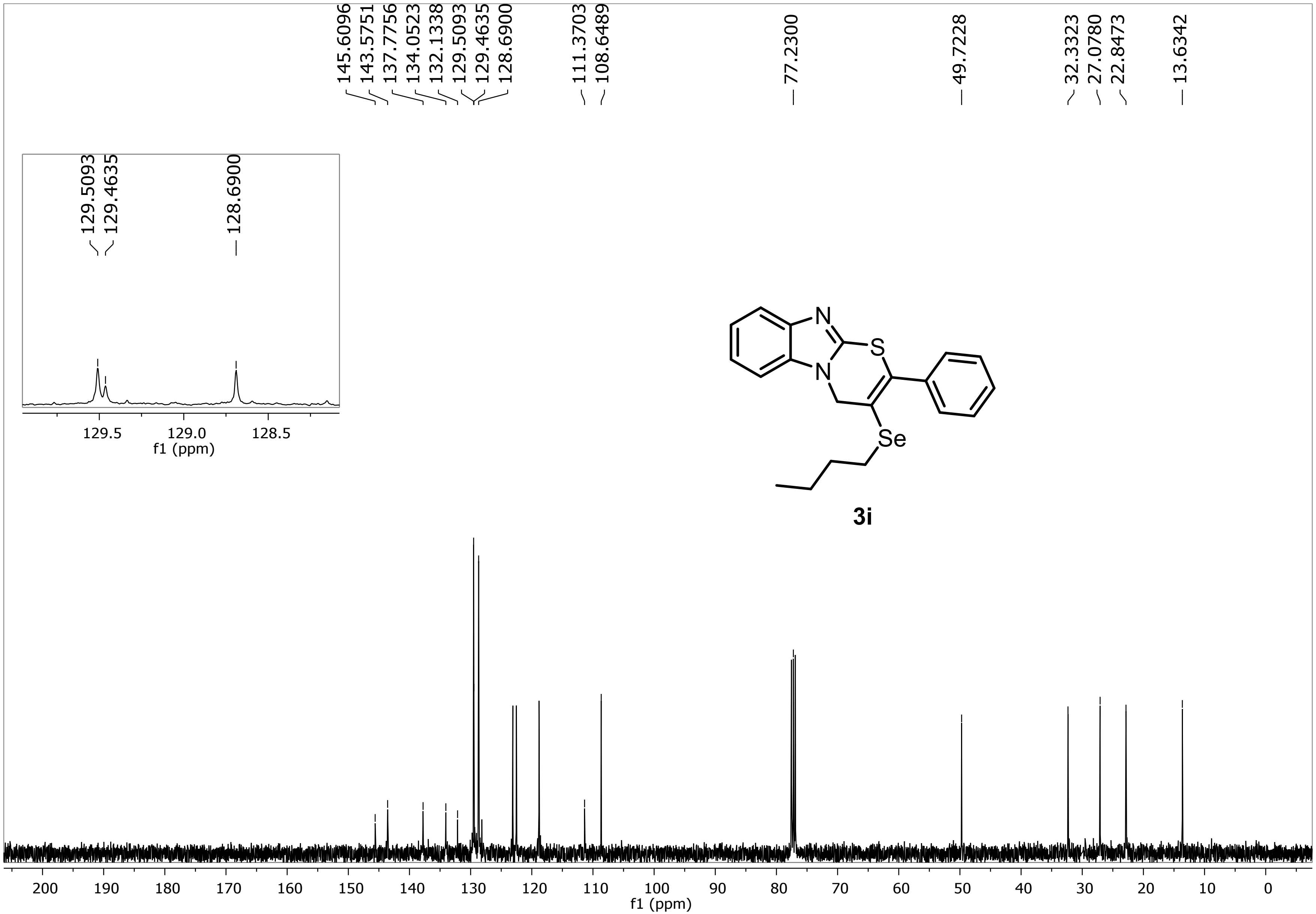

Figure S44: $^{13}C\{^{1}H\}$ NMR (100 MHz, $CDCl_3$) spectrum of compound **3i**.

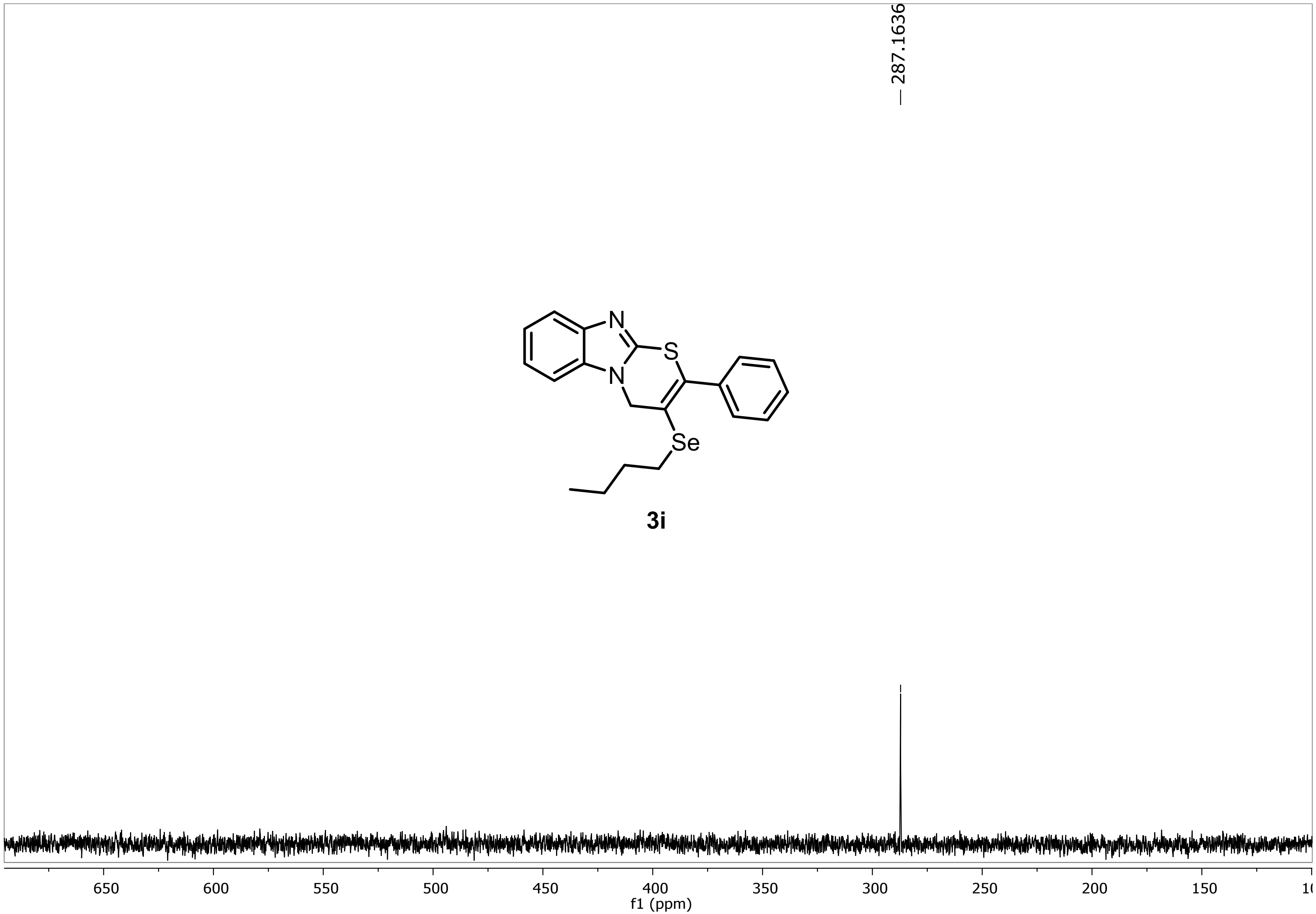

Figure S45: $^{77}Se\{^{1}H\}$ NMR (76 MHz, $CDCl_3$) spectrum of compound **3i**.

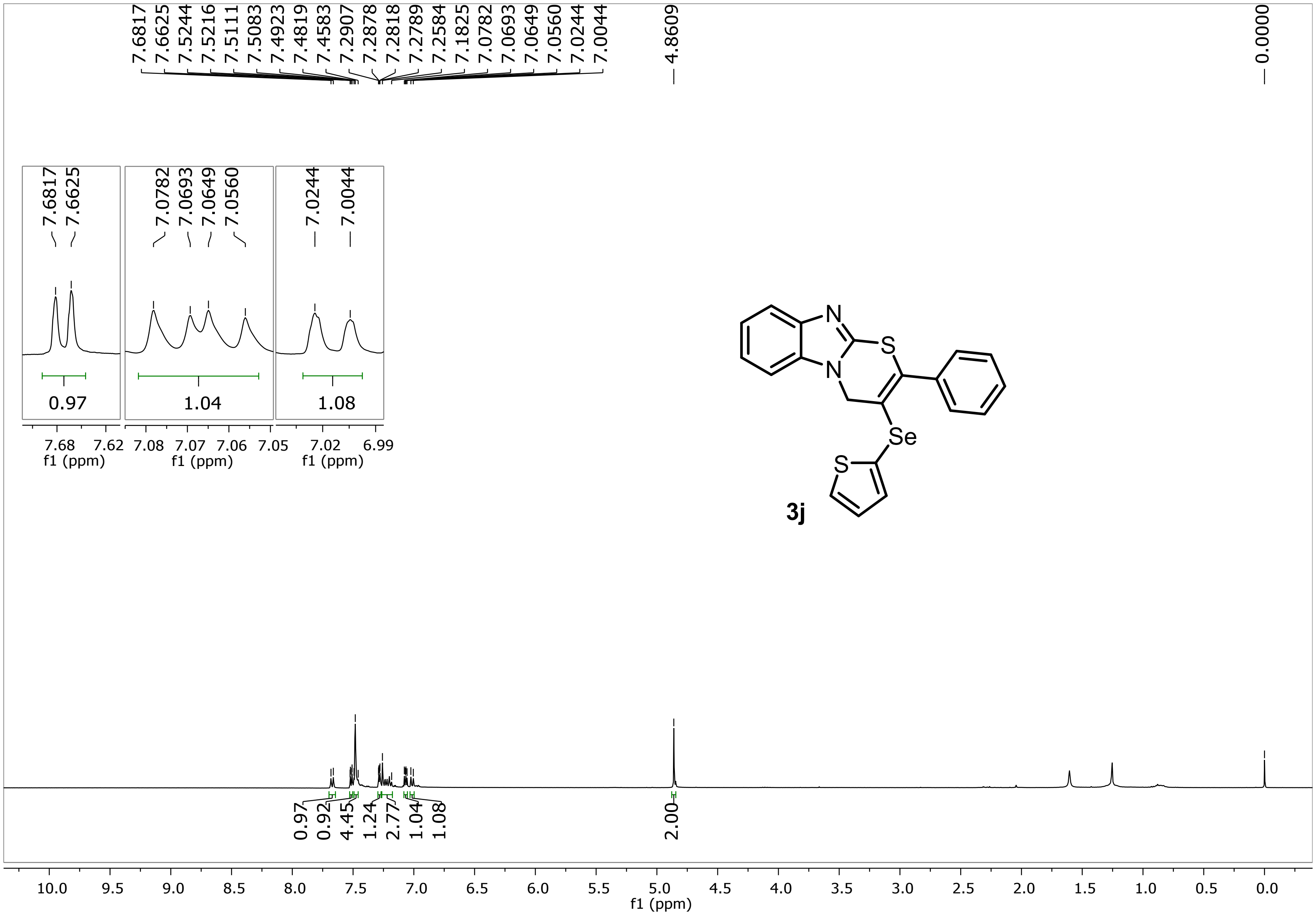

Figure S46: ^1H NMR (400 MHz, $CDCl_3$) spectrum of compound **3j**.

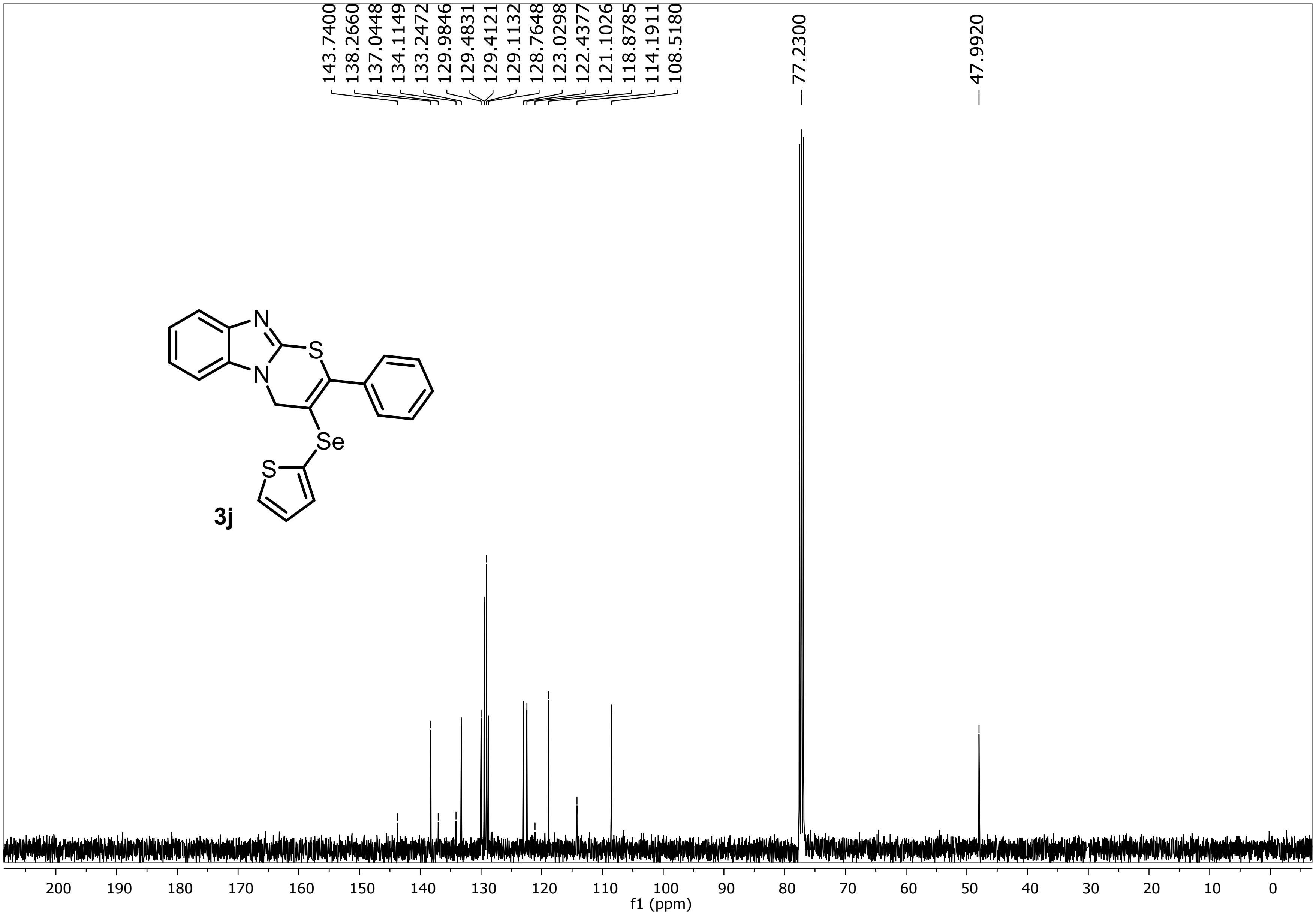

Figure S47: $^{13}C\{^{1}H\}$ NMR (100 MHz, $CDCl_3$) spectrum of compound **3j**.

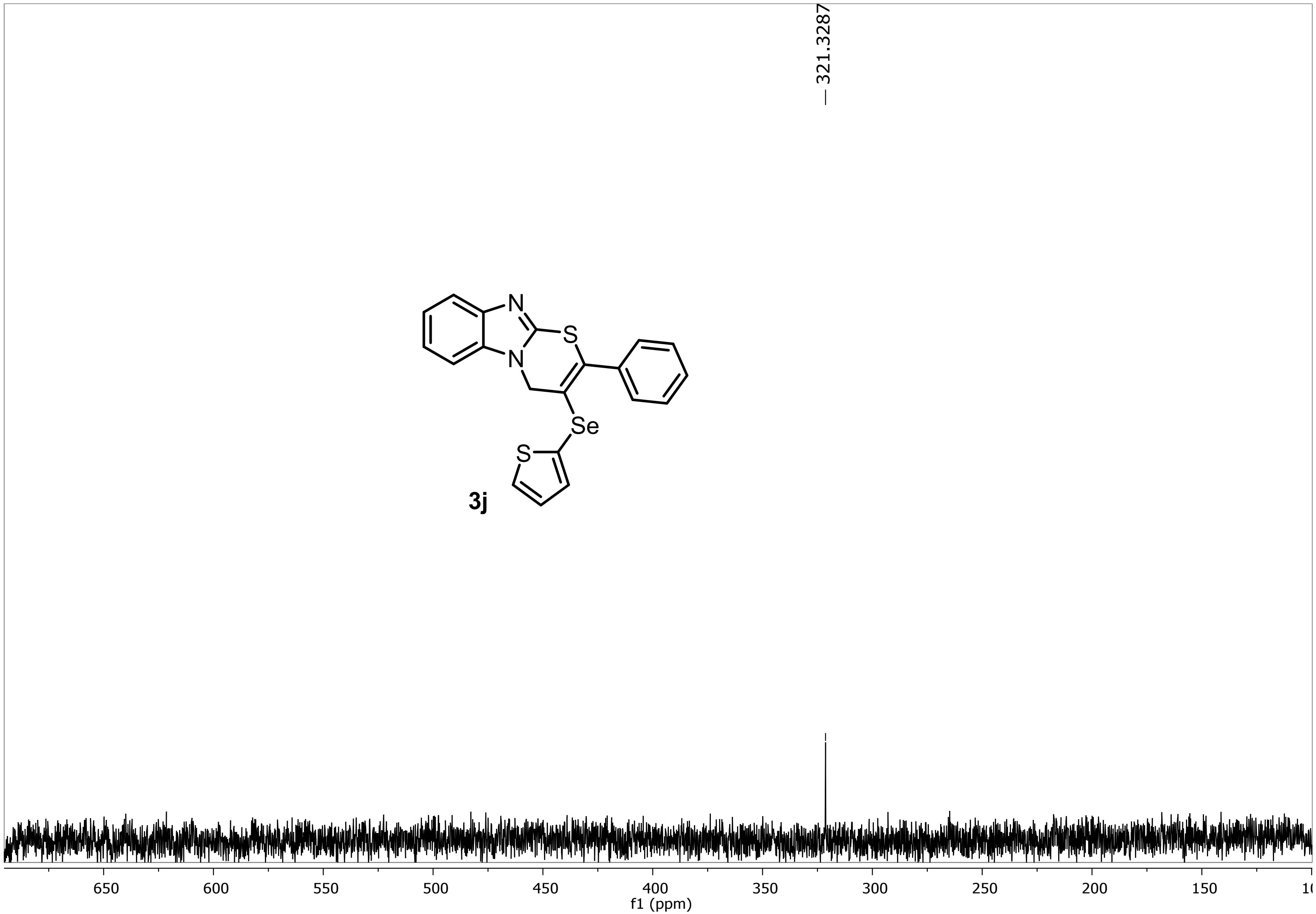

Figure S48: $^{77}Se\{^1H\}$ NMR (76 MHz, $CDCl_3$) spectrum of compound **3j**.

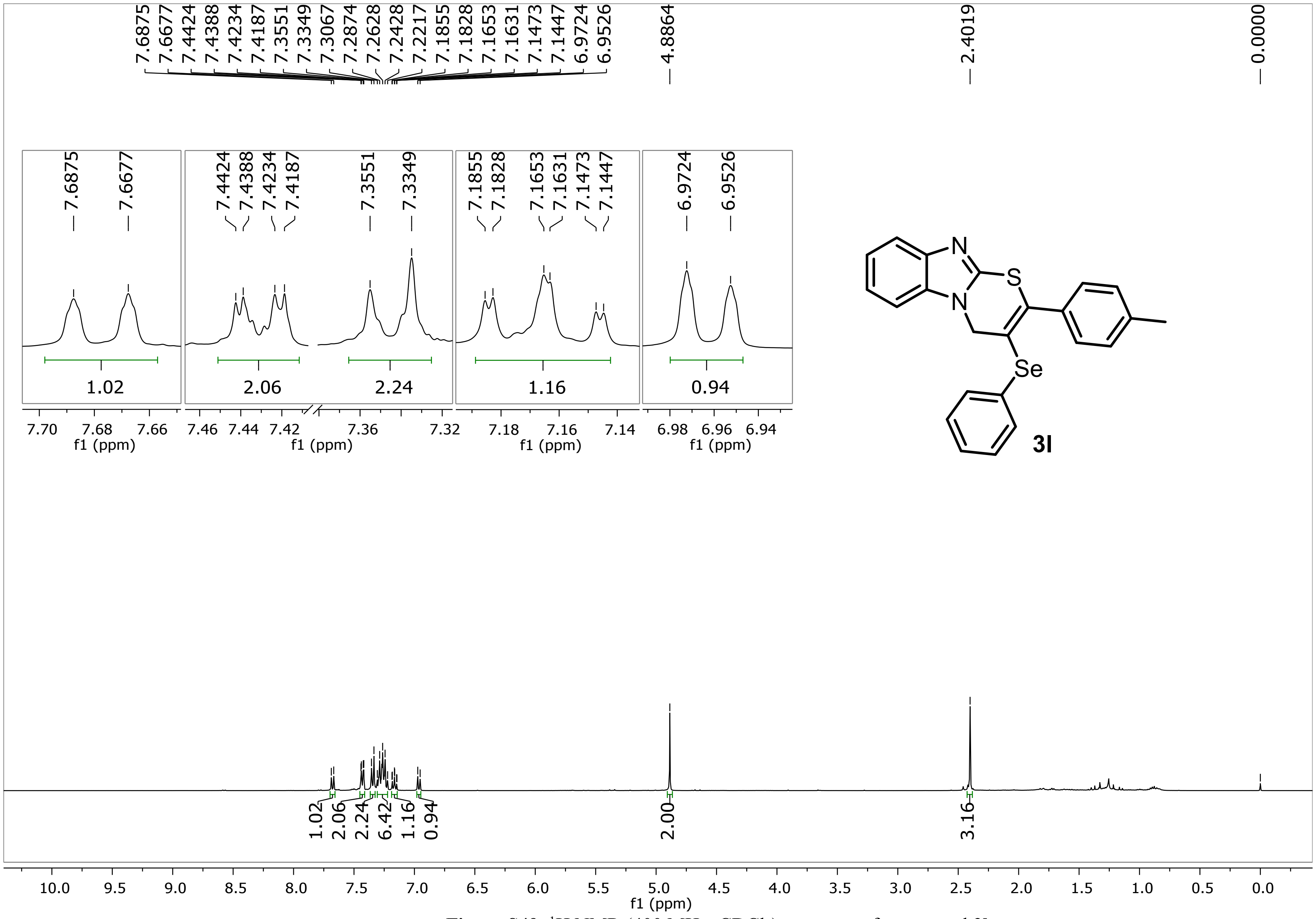

Figure S49: ^{1}H NMR (400 MHz, $CDCl_3$) spectrum of compound **3l**.

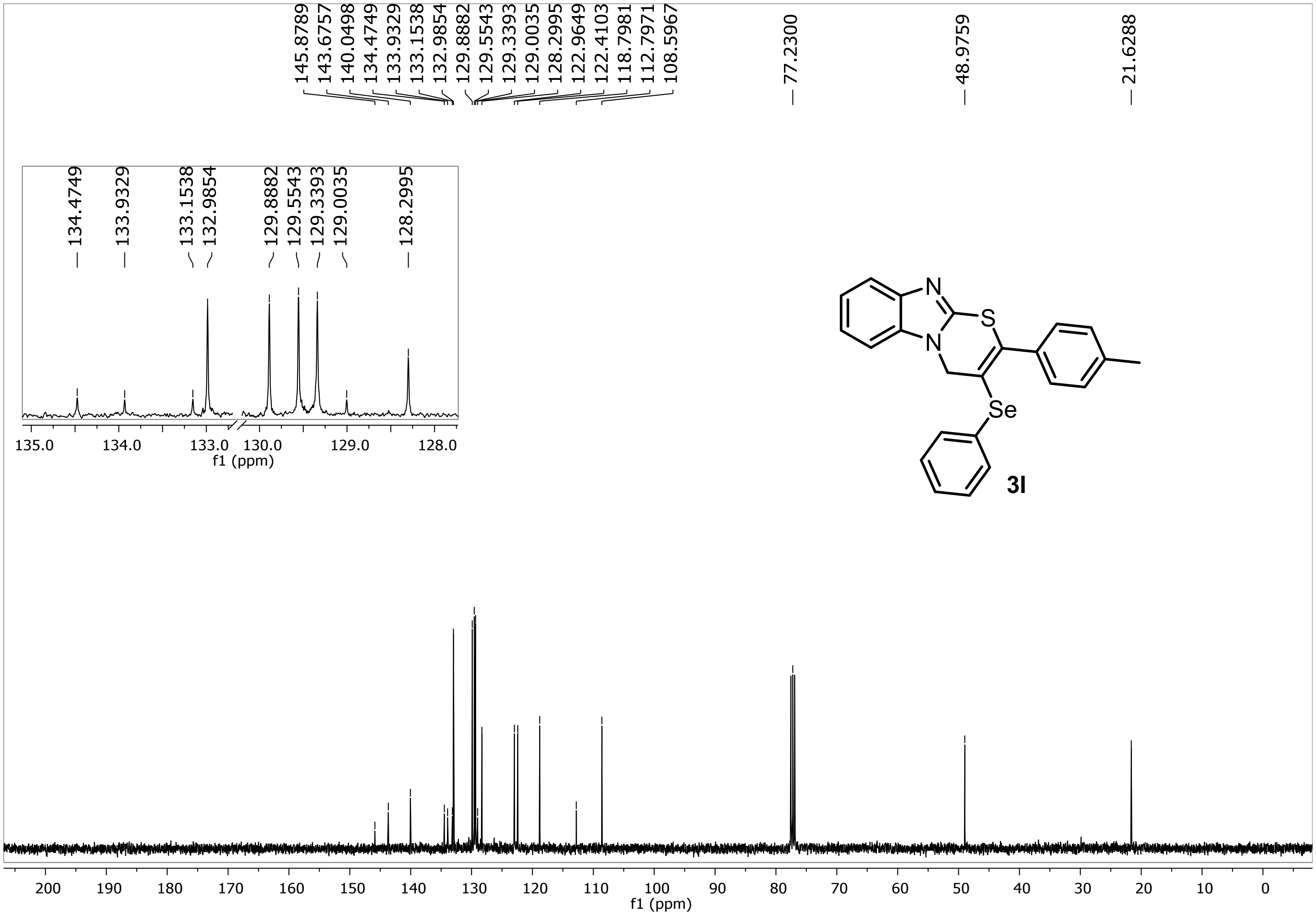

3l

Figure S50: $^{13}C\{^{1}H\}$ NMR (100 MHz, $CDCl_3$) spectrum of compound **3l**.

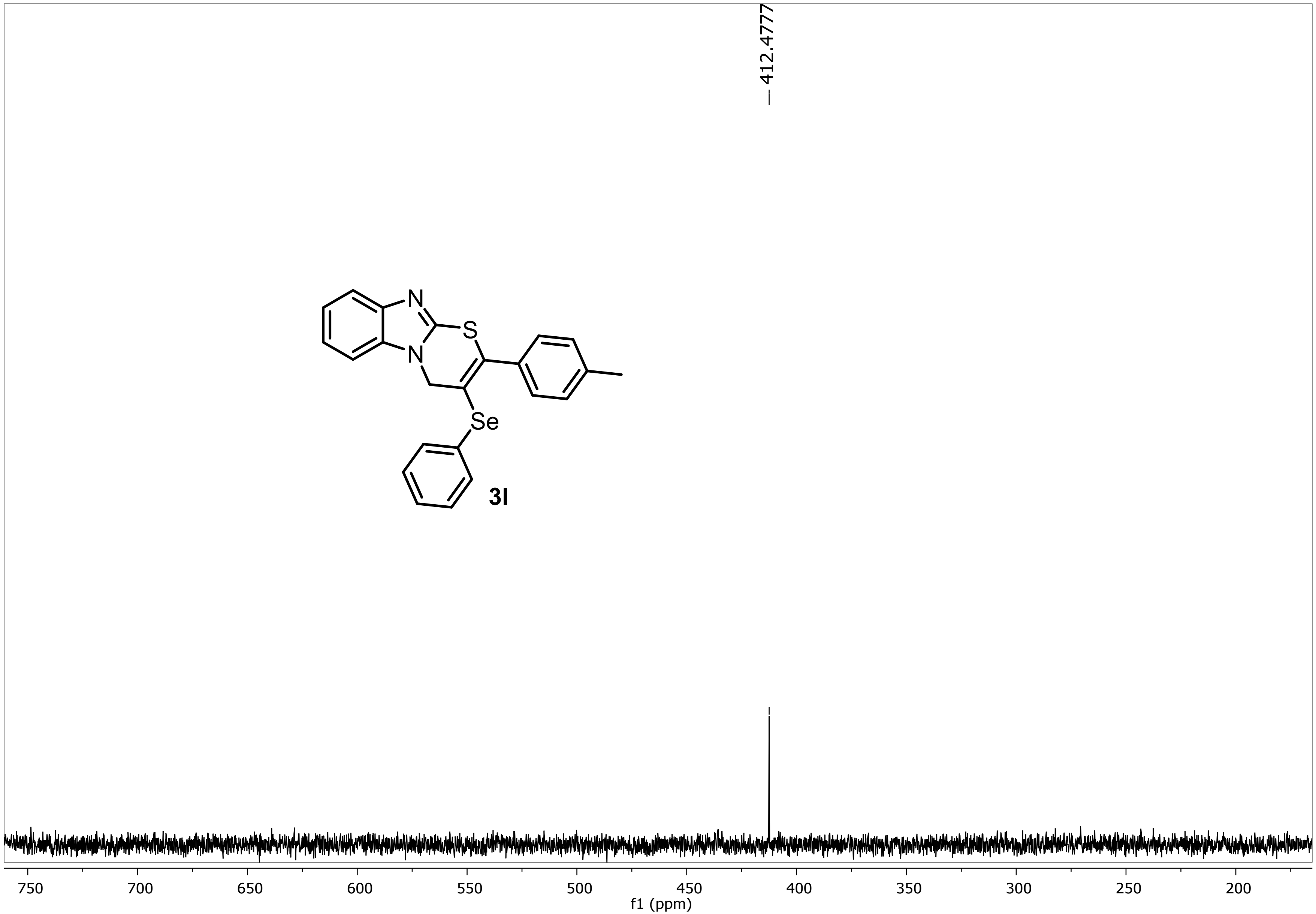

Figure S51: $^{77}Se\{^{1}H\}$ NMR (76 MHz, $CDCl_3$) spectrum of compound **3l**.

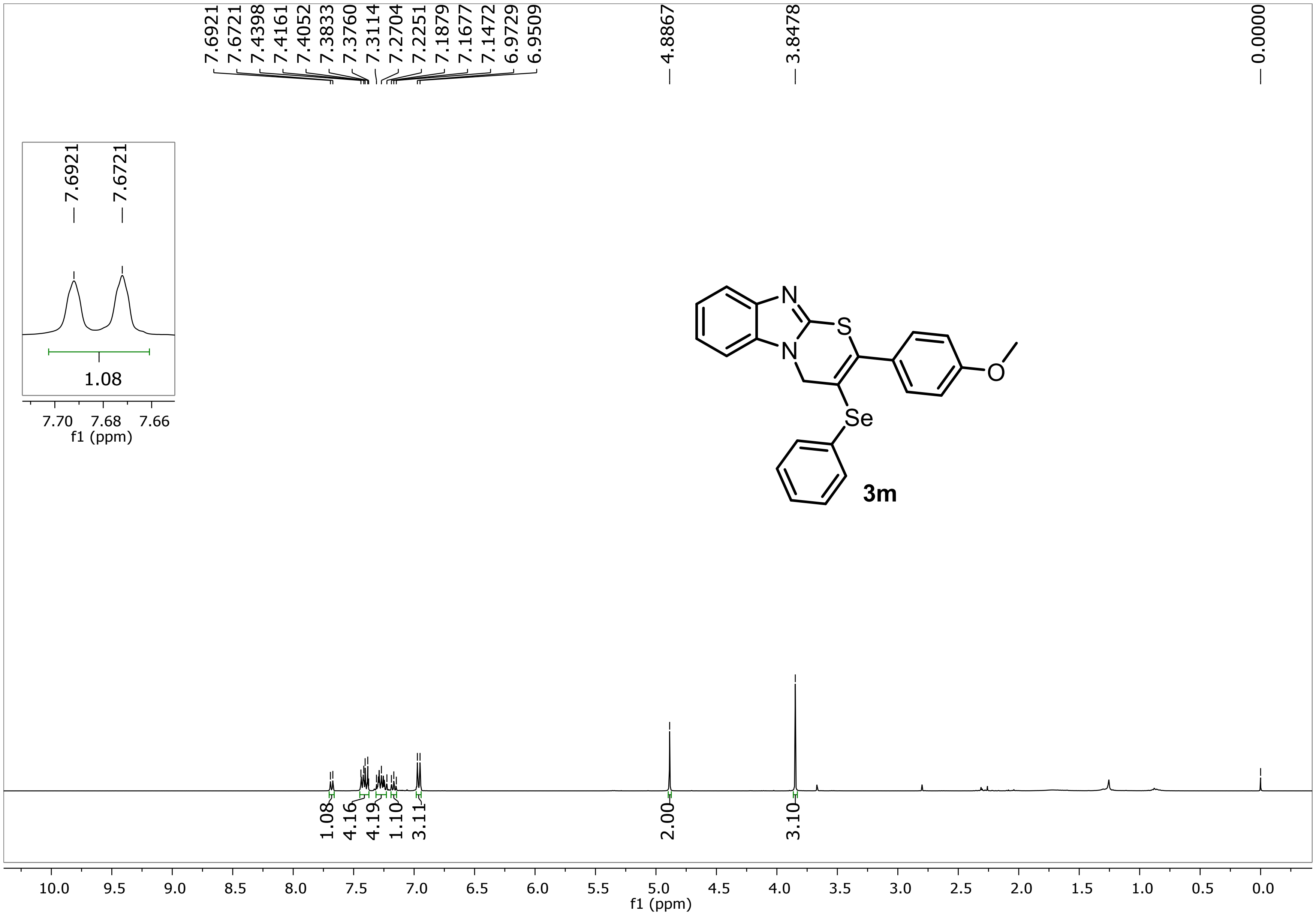

Figure S52: ^{1}H NMR (400 MHz, $CDCl_3$) spectrum of compound **3m**.

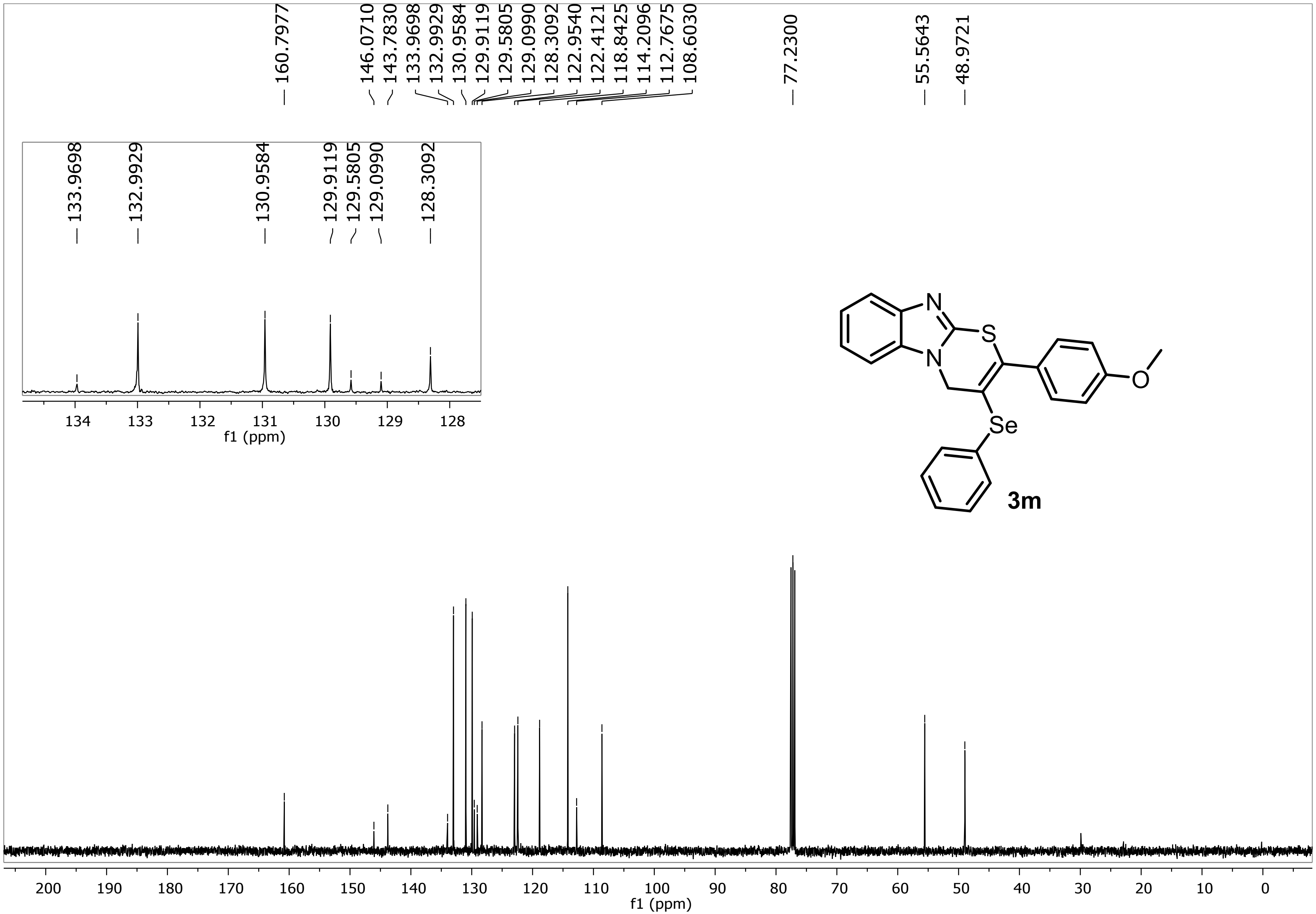

Figure S53: $^{13}C\{^{1}H\}$ NMR (100 MHz, $CDCl_3$) spectrum of compound **3m**.

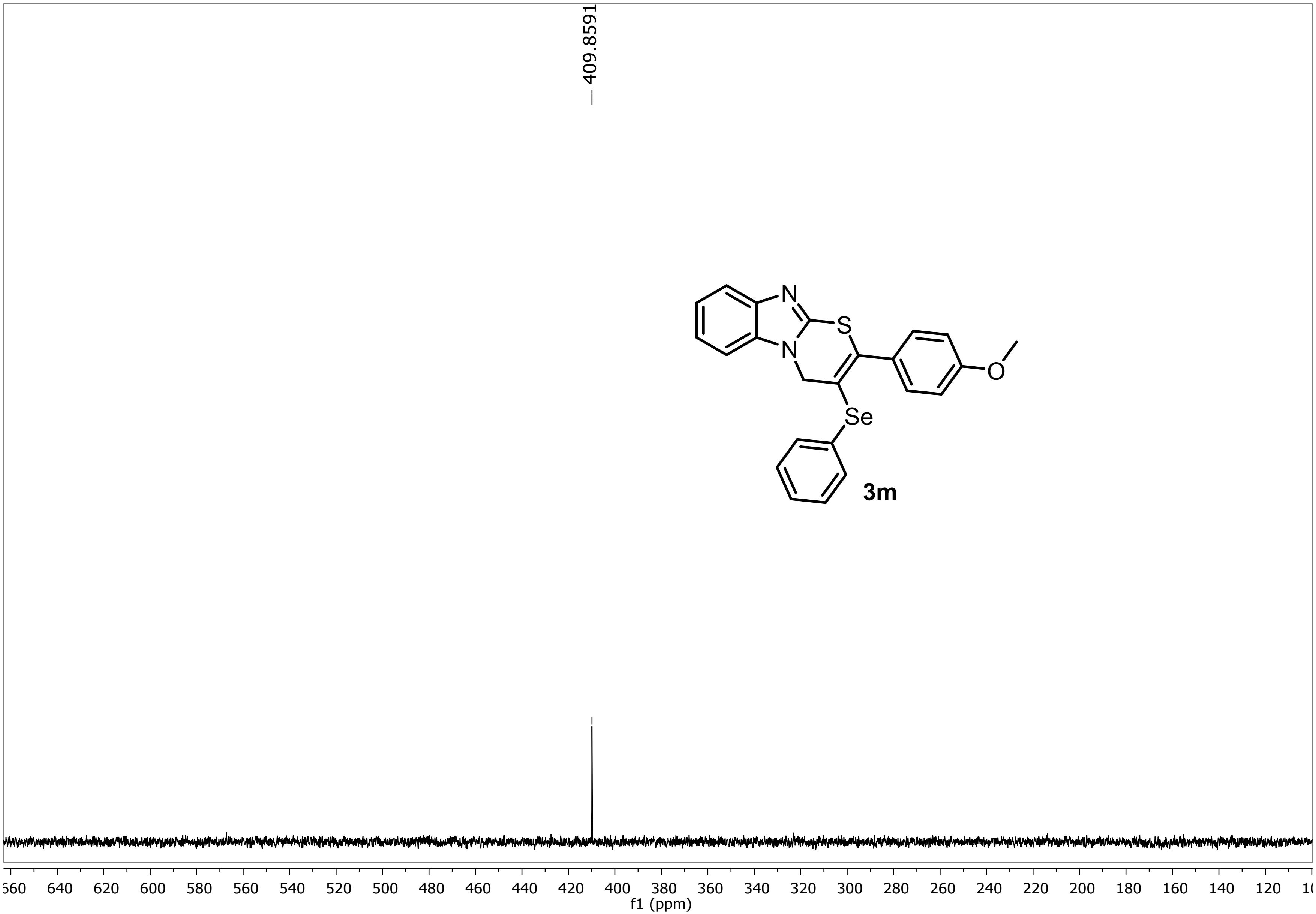

Figure S54: $^{77}Se\{^1H\}$ NMR (76 MHz, $CDCl_3$) spectrum of compound **3m**.

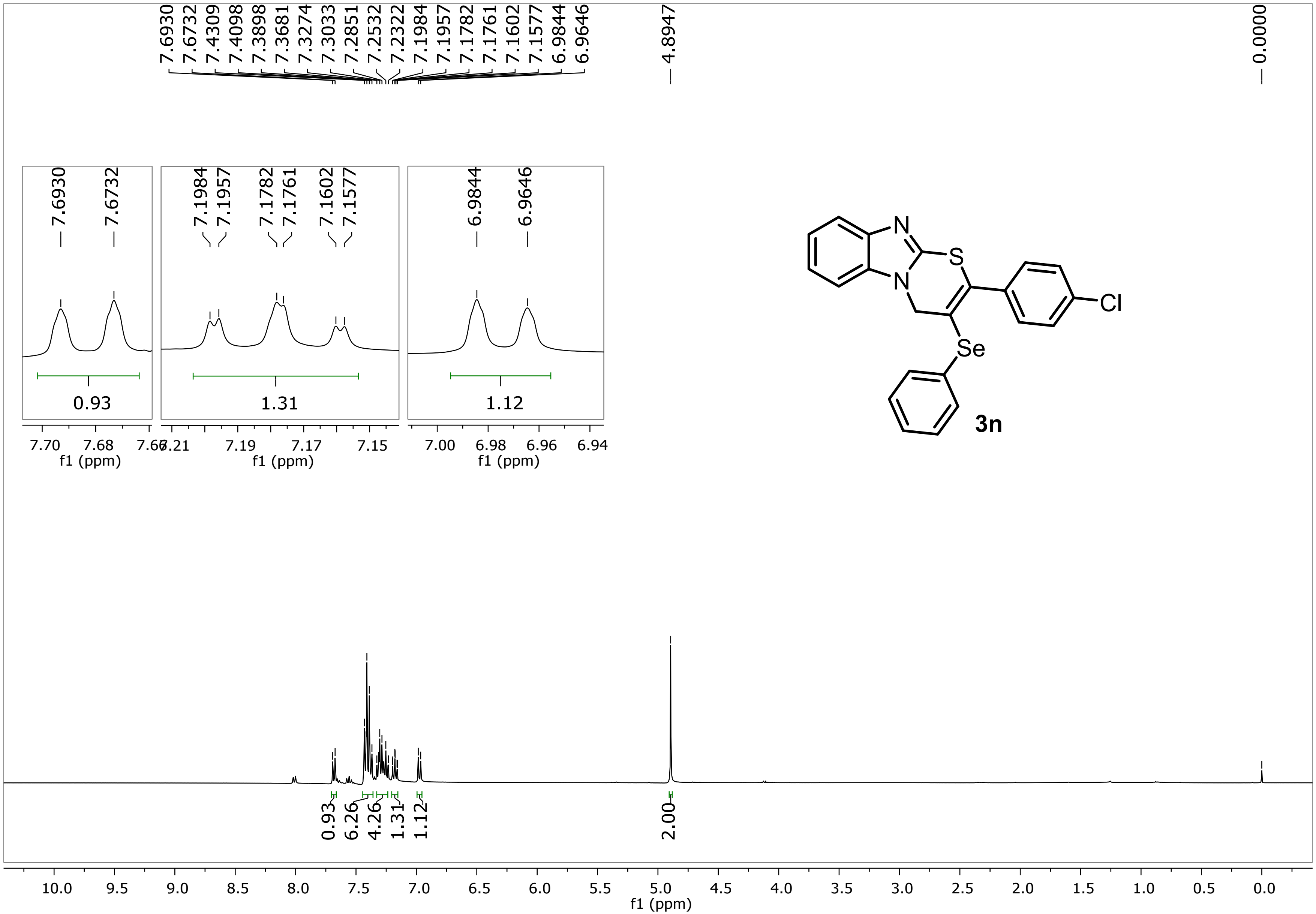

Figure S55: ^{1}H NMR (400 MHz, $CDCl_3$) spectrum of compound **3n**.

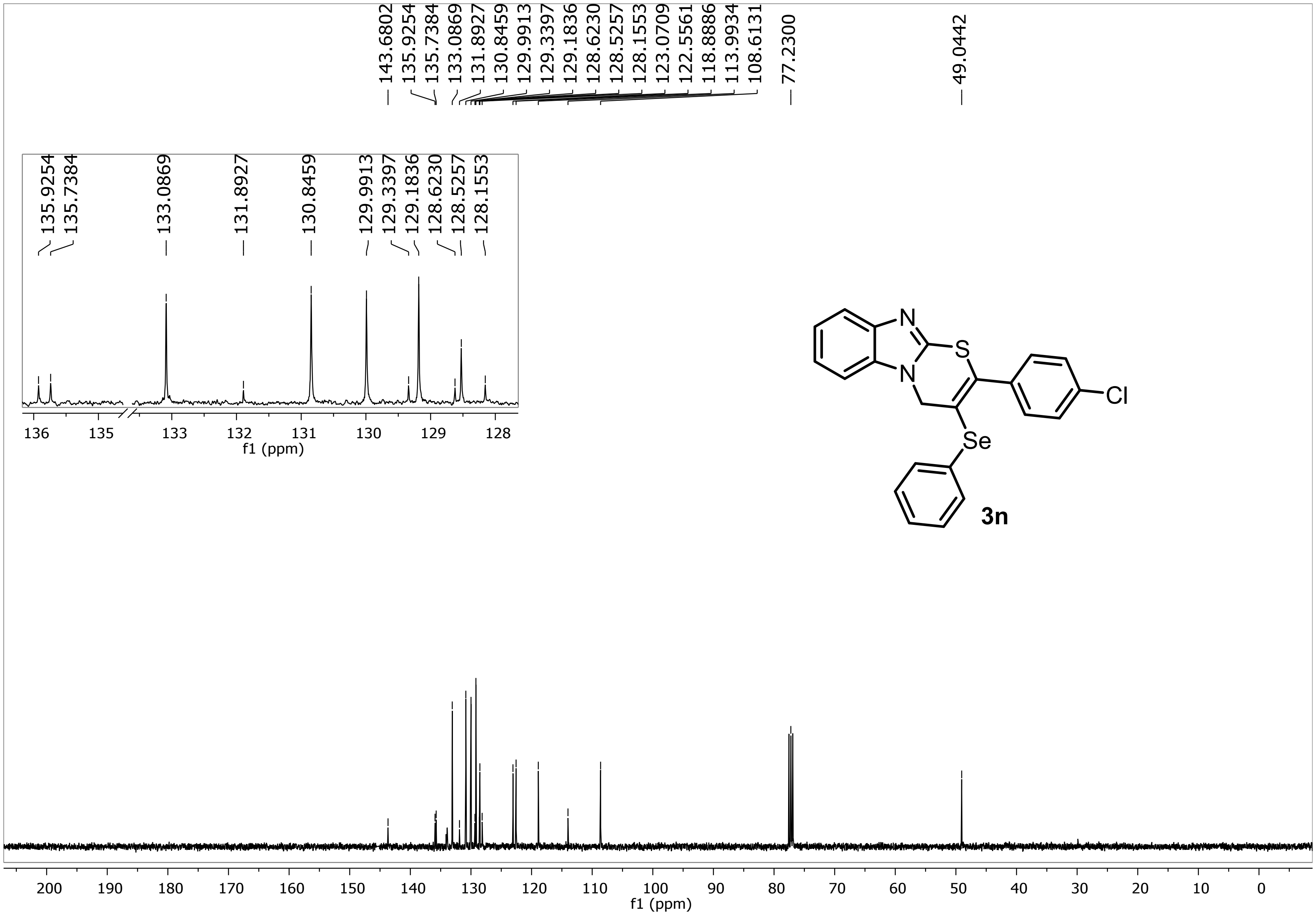

Figure S56: $^{13}C\{^{1}H\}$ NMR (100 MHz, $CDCl_3$) spectrum of compound **3n**.

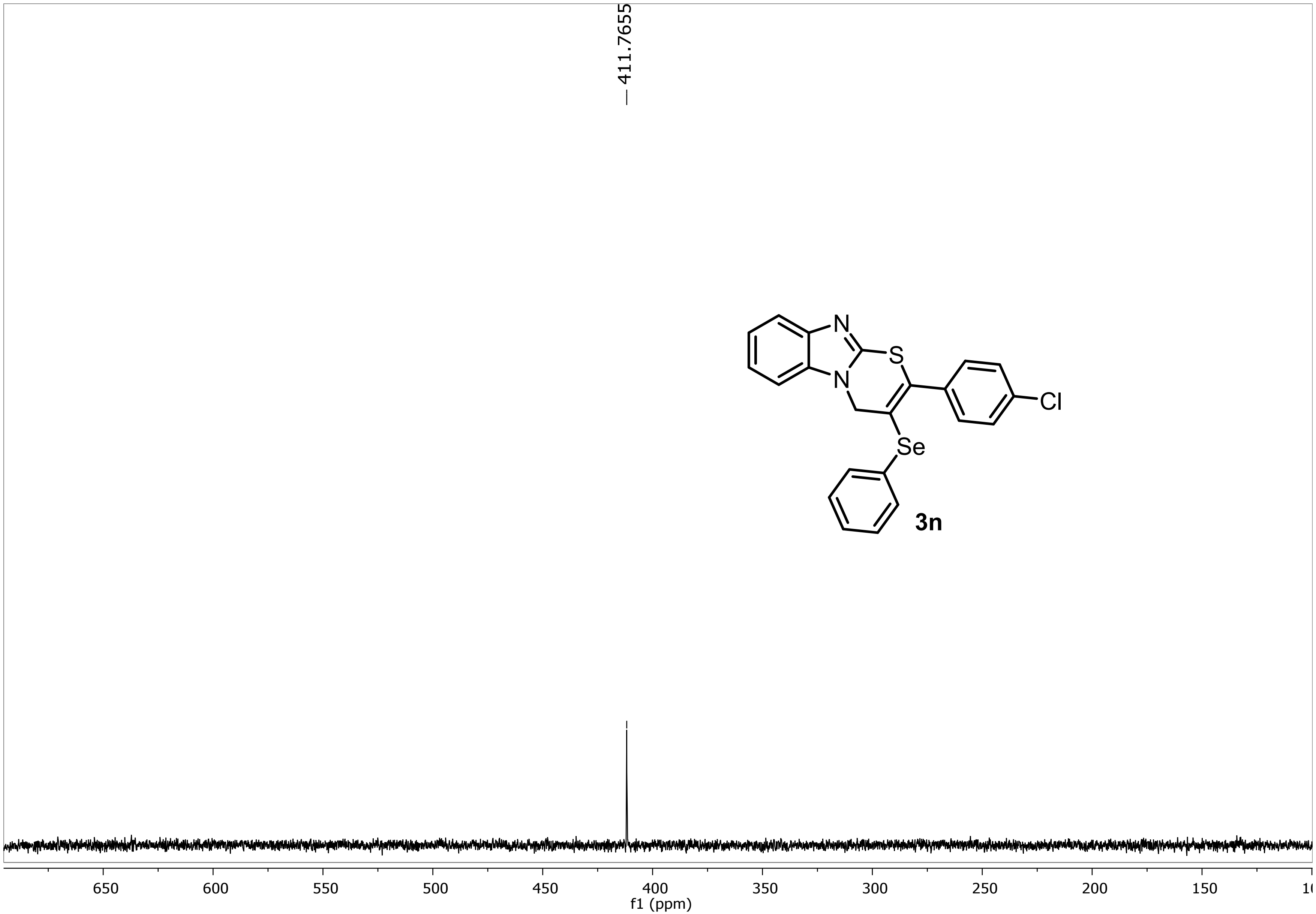

Figure S57: $^{77}Se\{^{1}H\}$ NMR (76 MHz, $CDCl_3$) spectrum of compound **3n**.

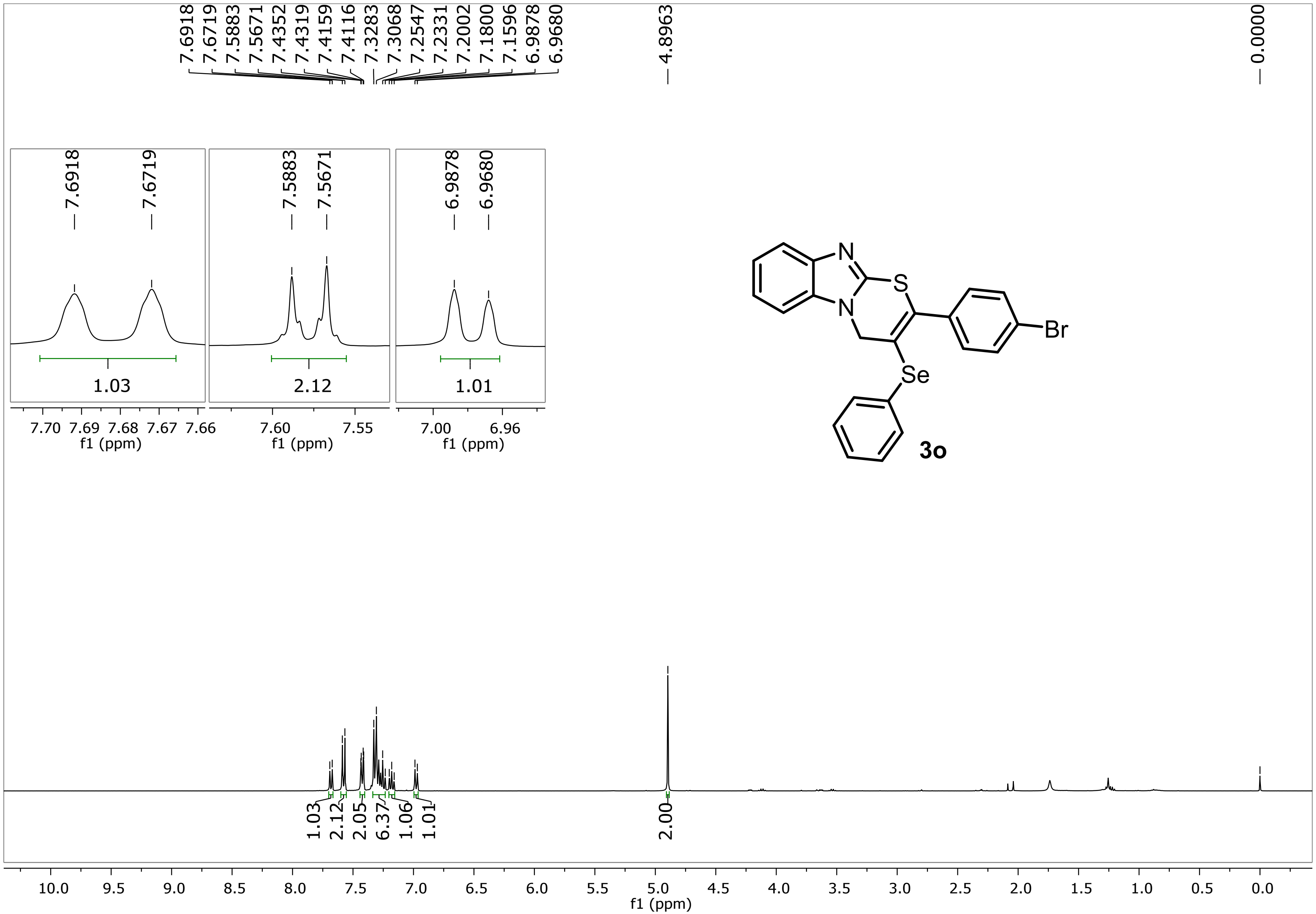

Figure S58: ^{1}H NMR (400 MHz, $CDCl_3$) spectrum of compound **3o**.

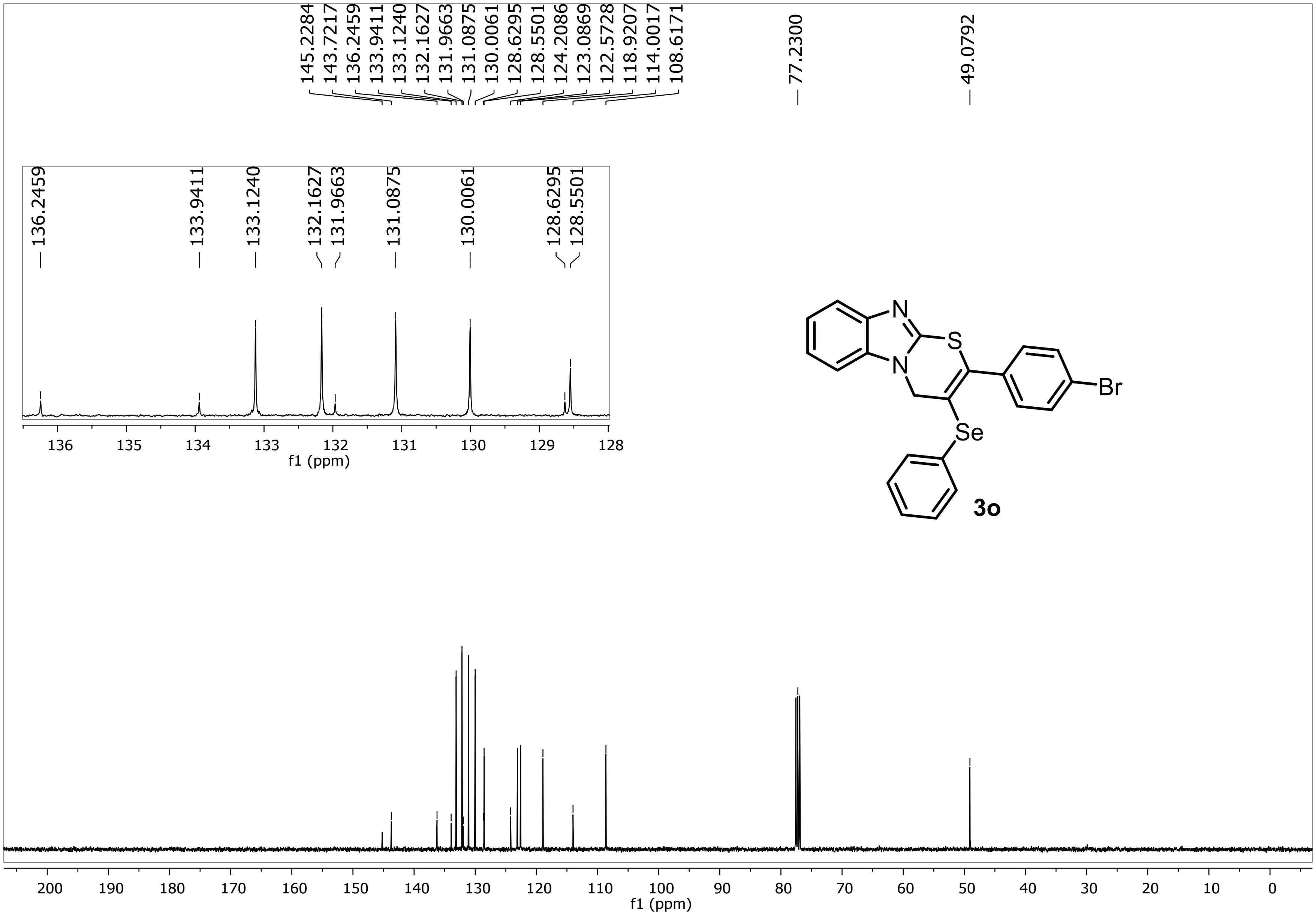

Figure S59: $^{13}C\{^{1}H\}$ NMR (100 MHz, $CDCl_3$) spectrum of compound **3o**.

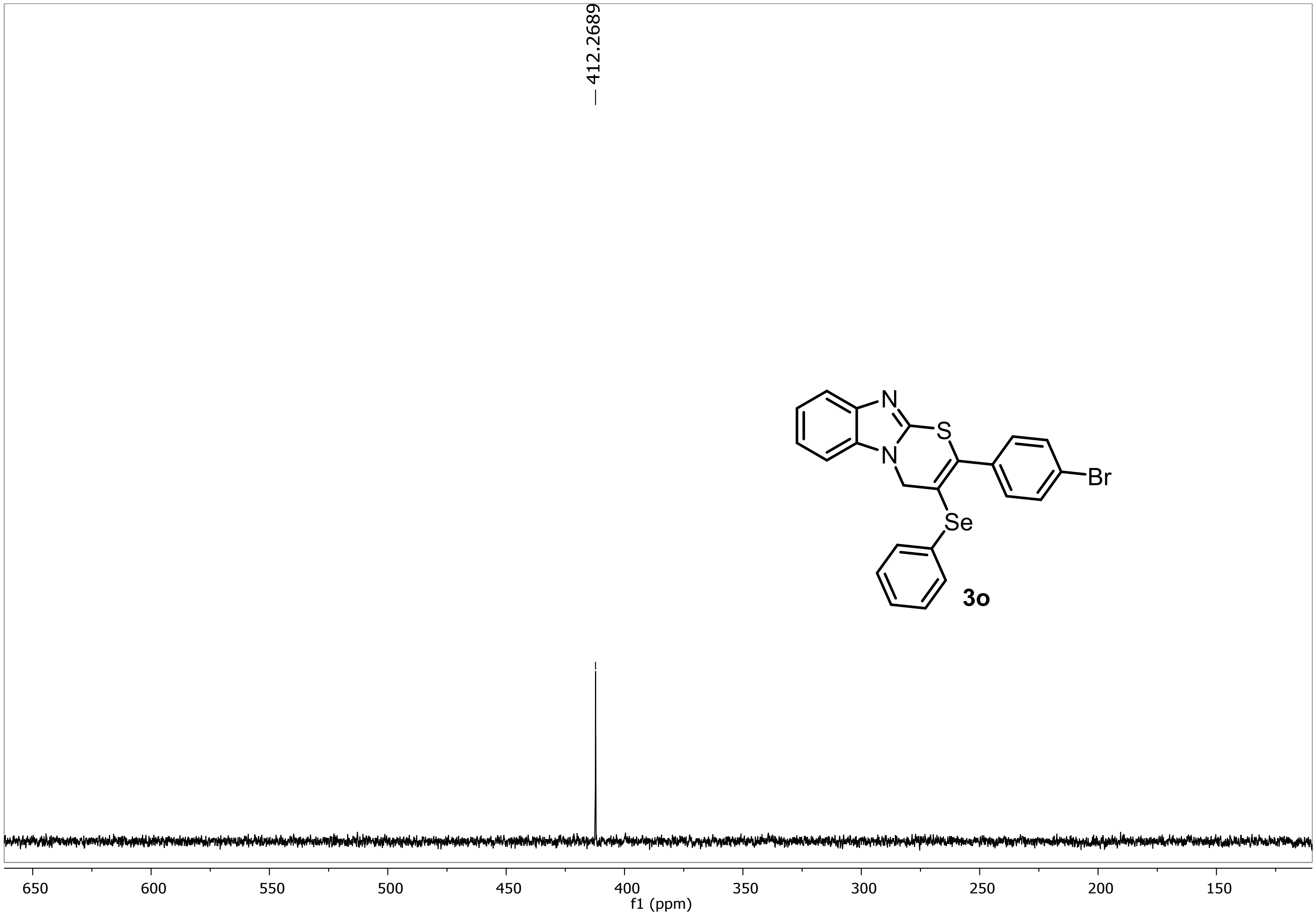

Figure S60: $^{77}Se\{^1H\}$ NMR (76 MHz, $CDCl_3$) spectrum of compound **3o**.

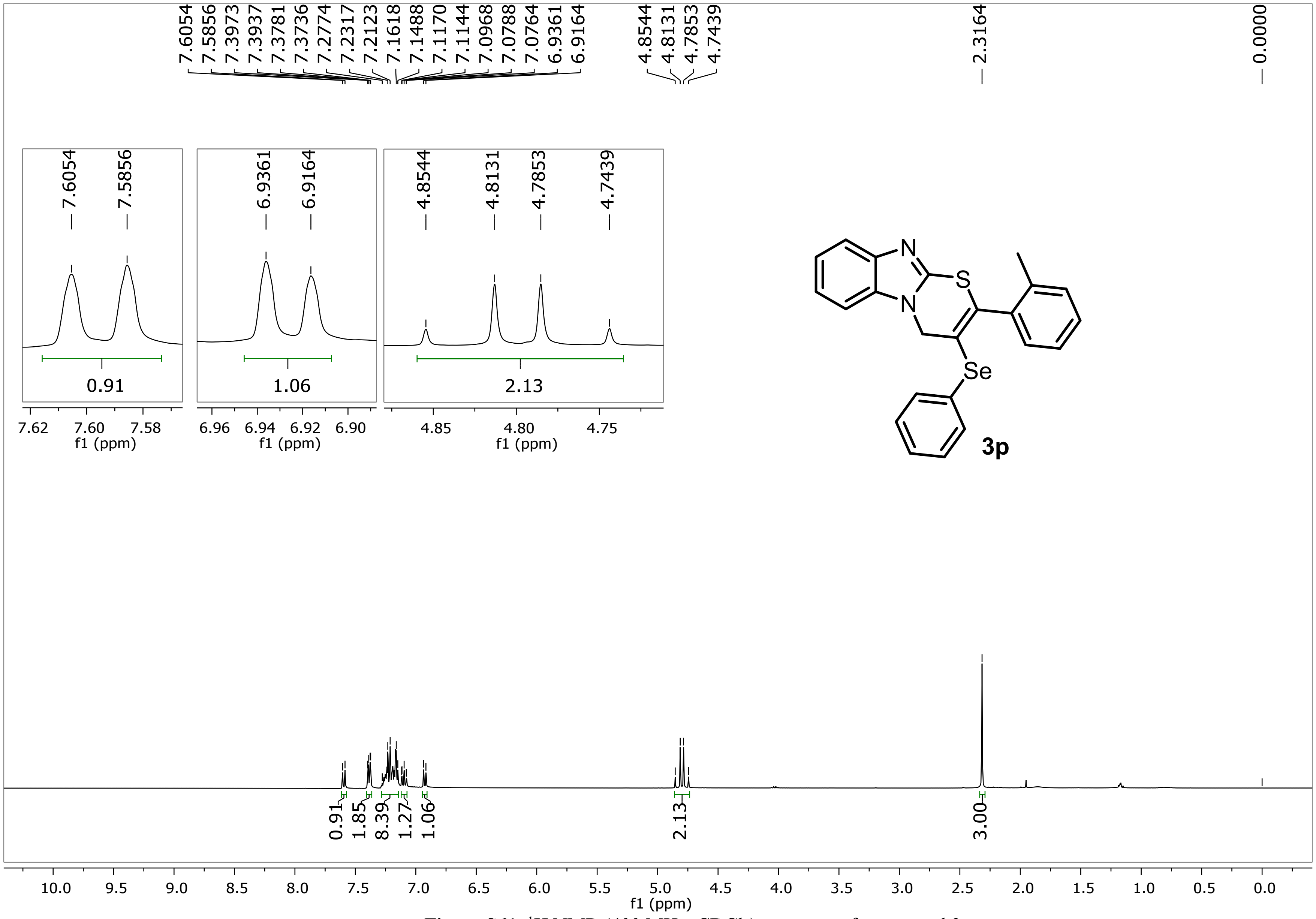

Figure S61: ^{1}H NMR (400 MHz, $CDCl_3$) spectrum of compound **3p**.

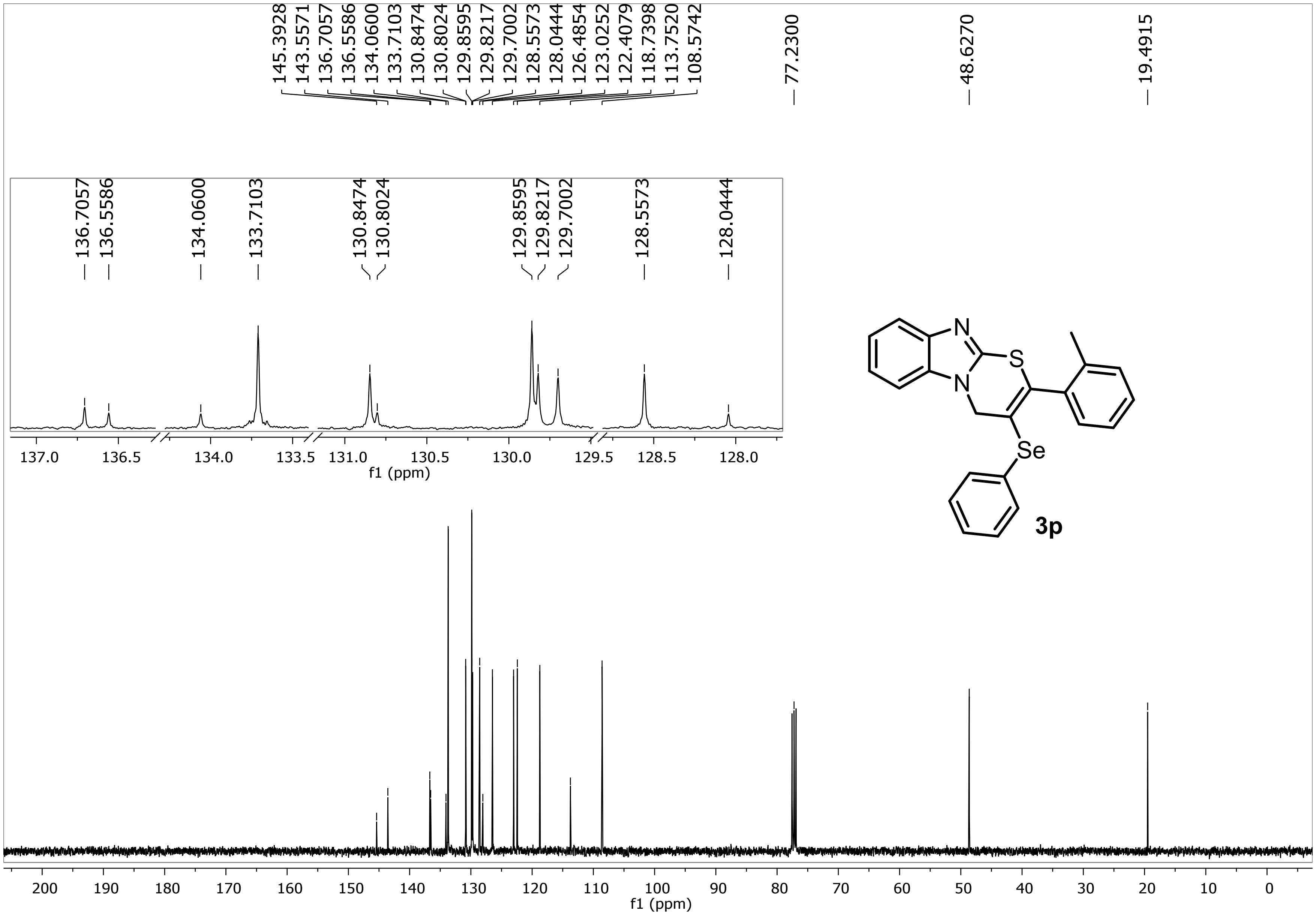

3p

Figure S62: $^{13}C\{^1H\}$ NMR (100 MHz, $CDCl_3$) spectrum of compound **3p**.

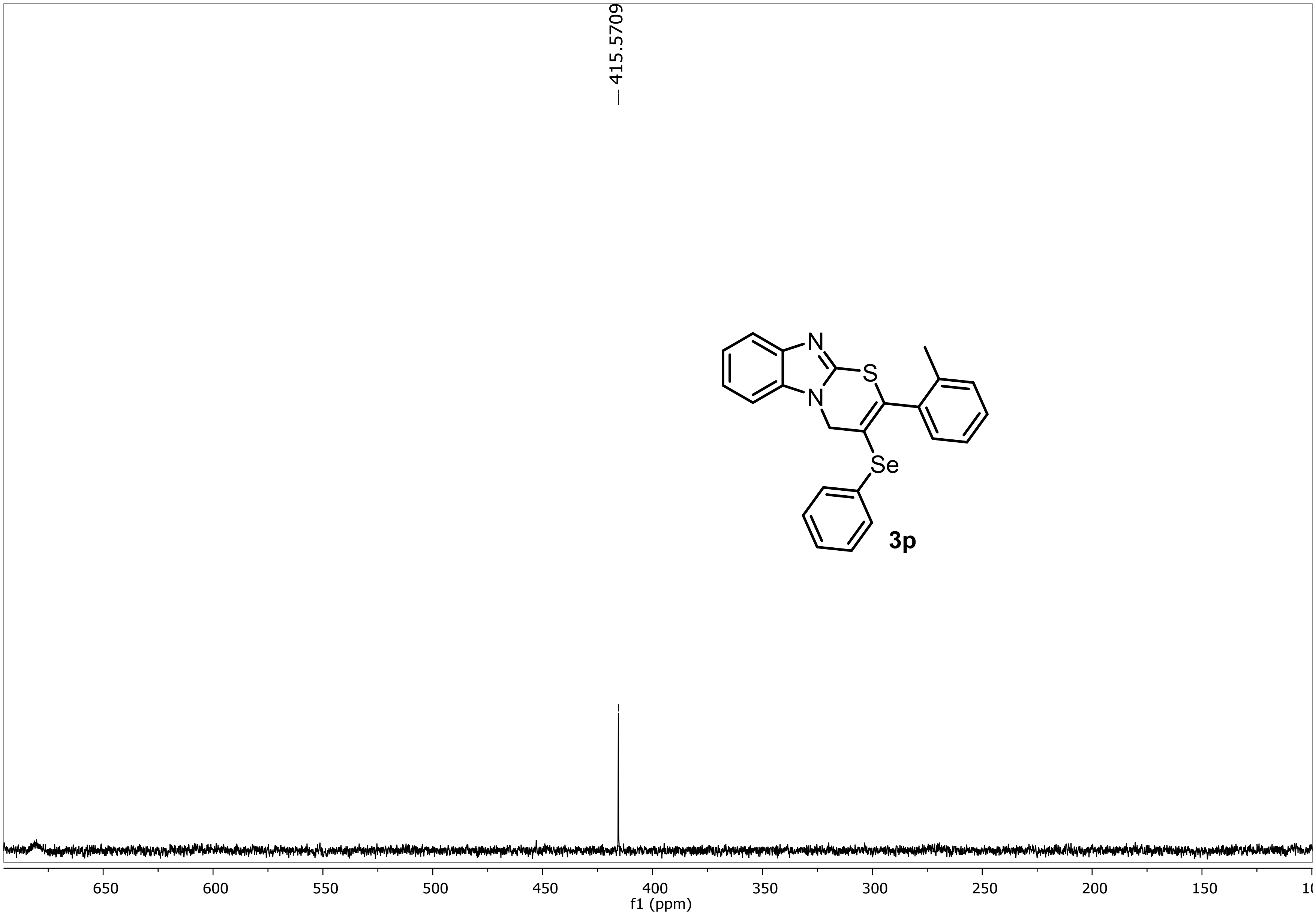

3p

Figure S63: $^{77}Se\{^1H\}$ NMR (76 MHz, $CDCl_3$) spectrum of compound **3p**.

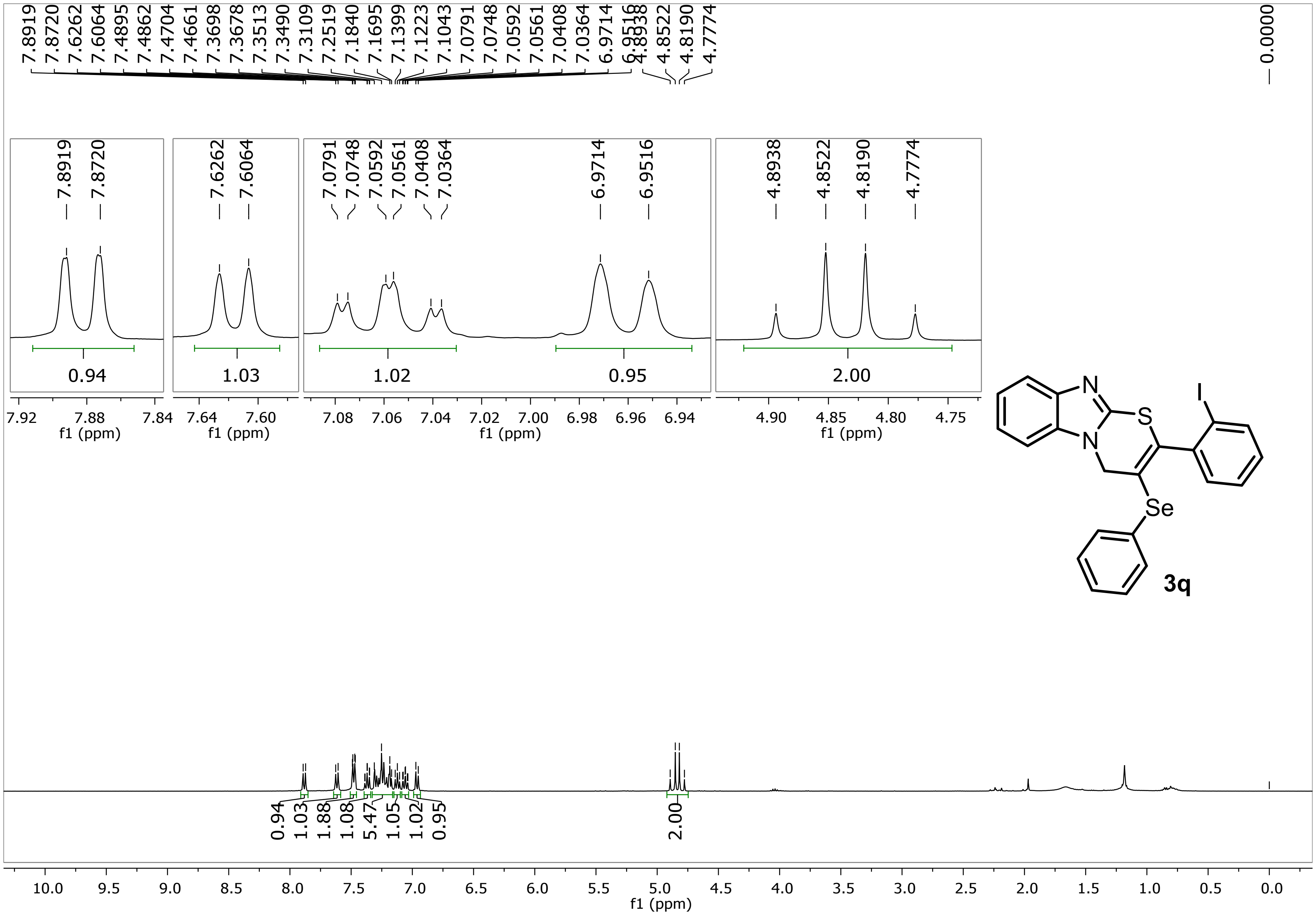

Figure S64: ^{1}H NMR (400 MHz, $CDCl_3$) spectrum of compound **3q**.

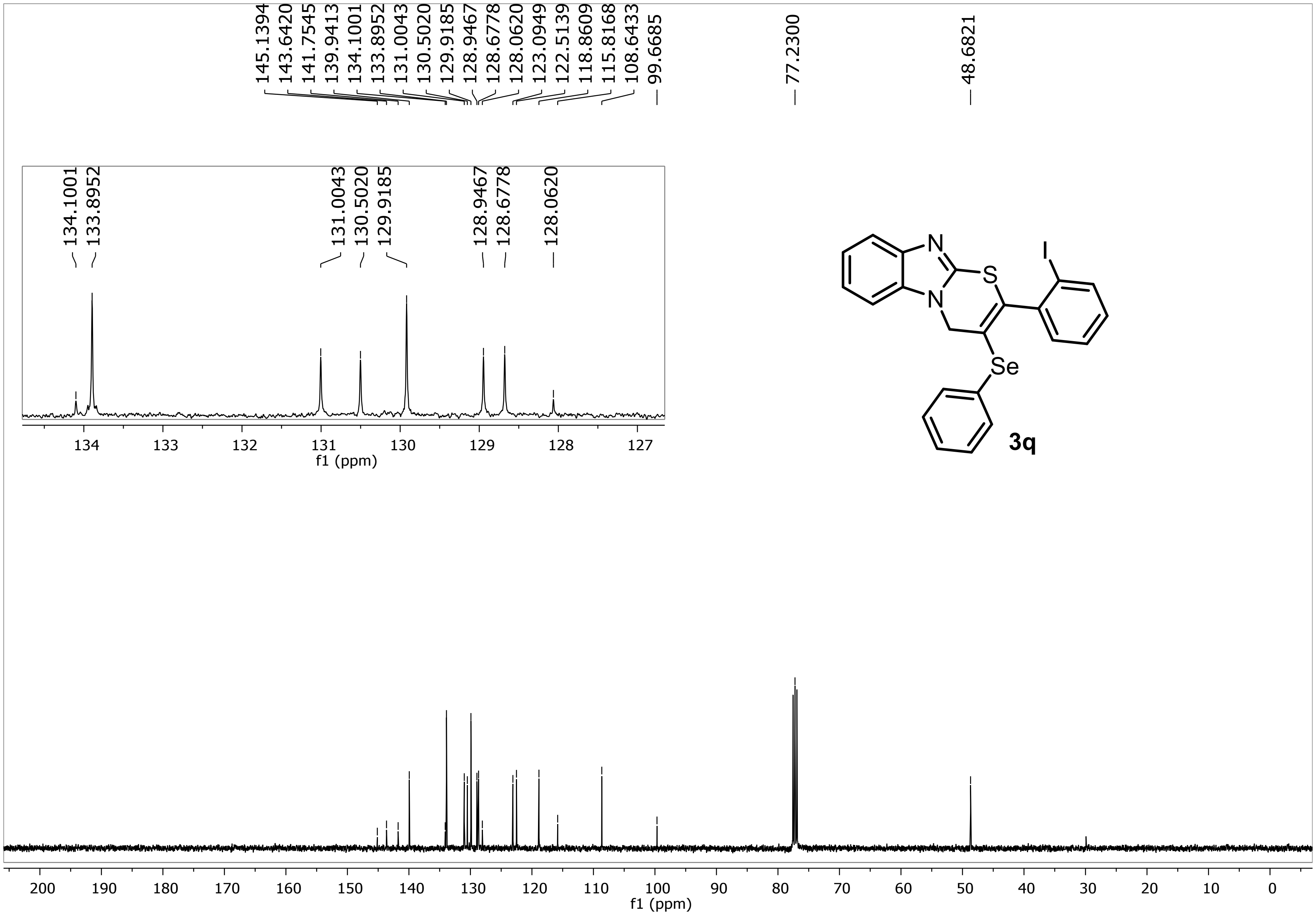

3q

Figure S65: $^{13}C\{^1H\}$ NMR (100 MHz, $CDCl_3$) spectrum of compound **3q**.

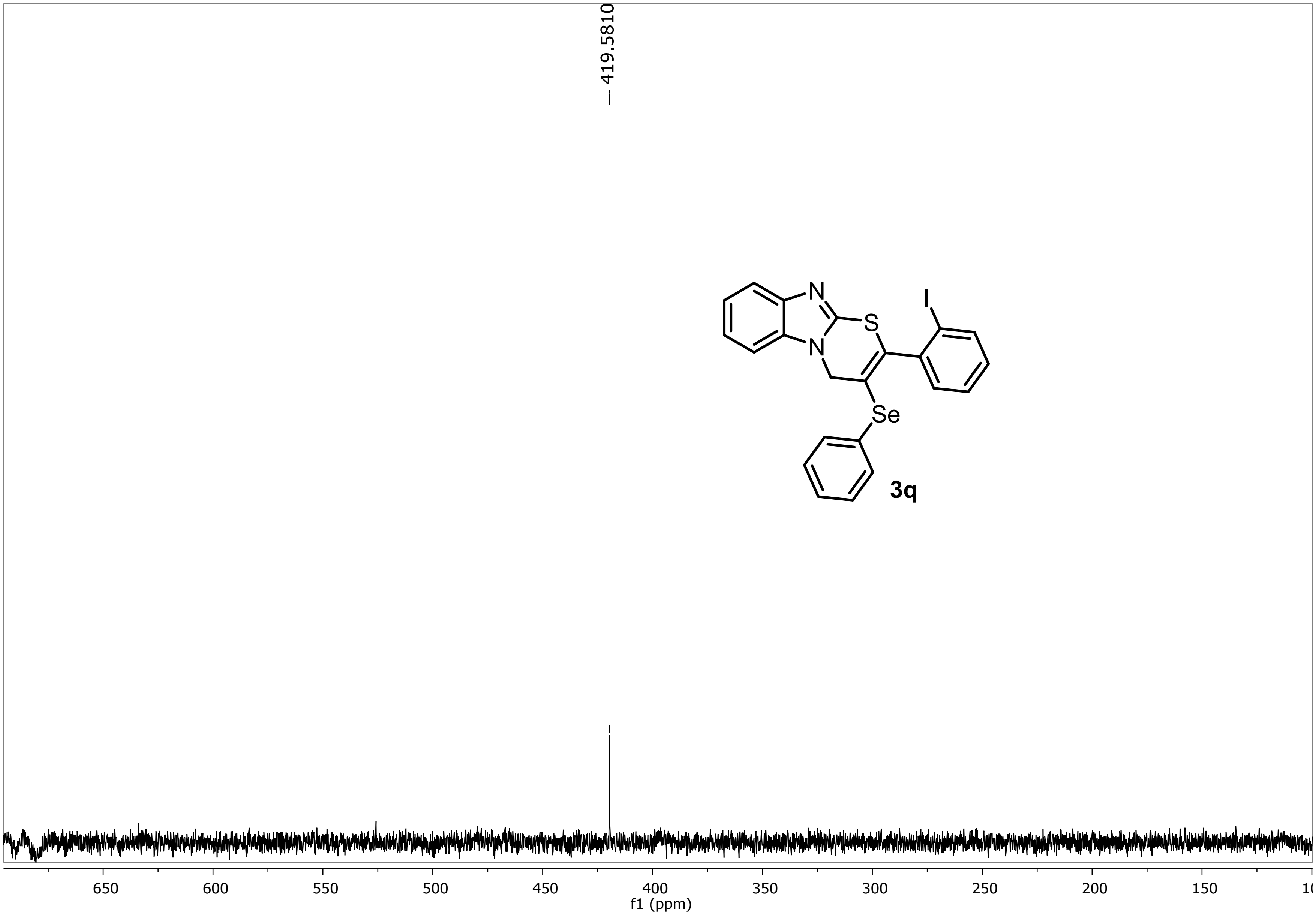

Figure S66: $^{77}Se\{^1H\}$ NMR (76 MHz, $CDCl_3$) spectrum of compound **3q**.

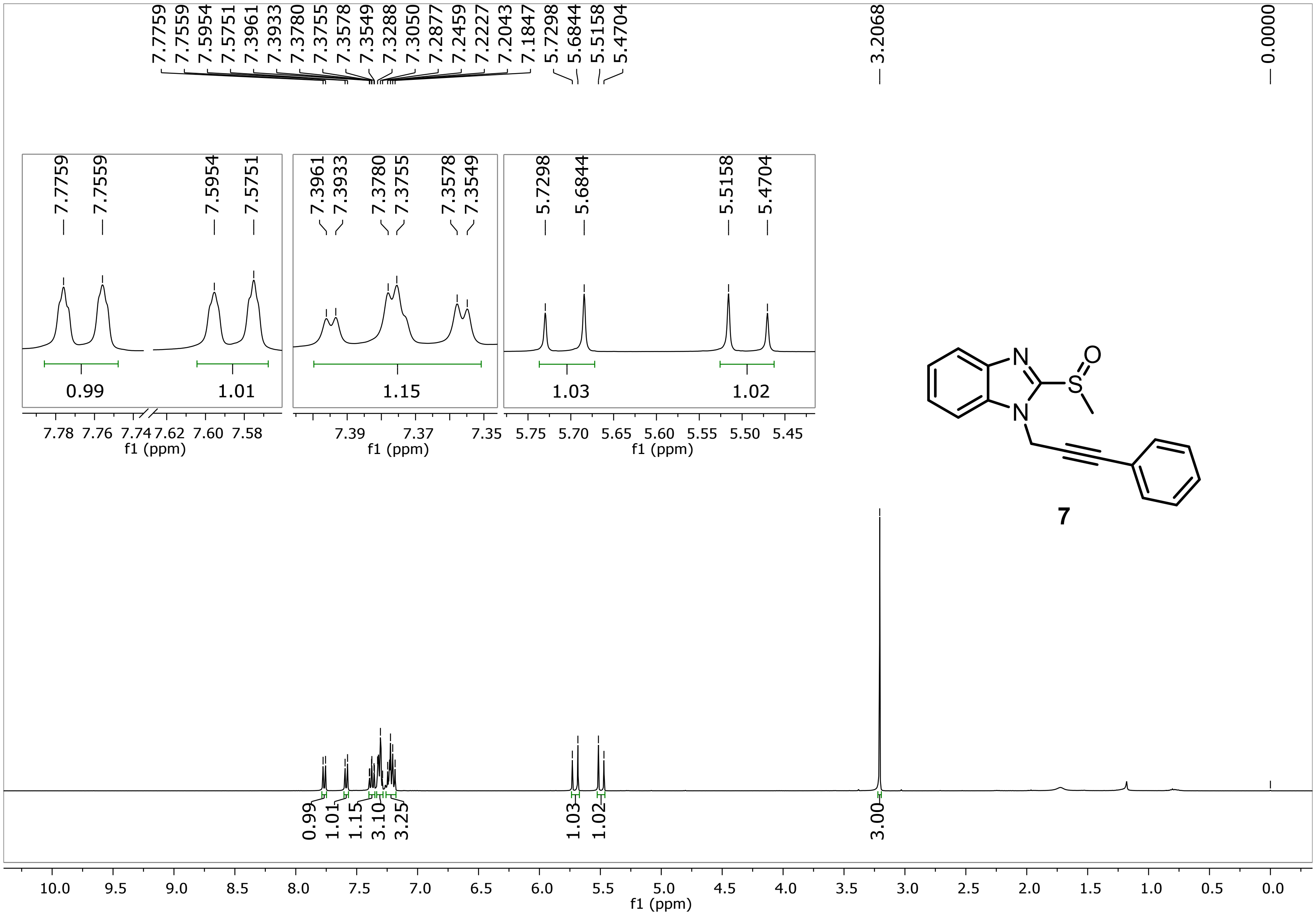

7

Figure S67: ^{1}H NMR (400 MHz, $CDCl_3$) spectrum of compound **7**.

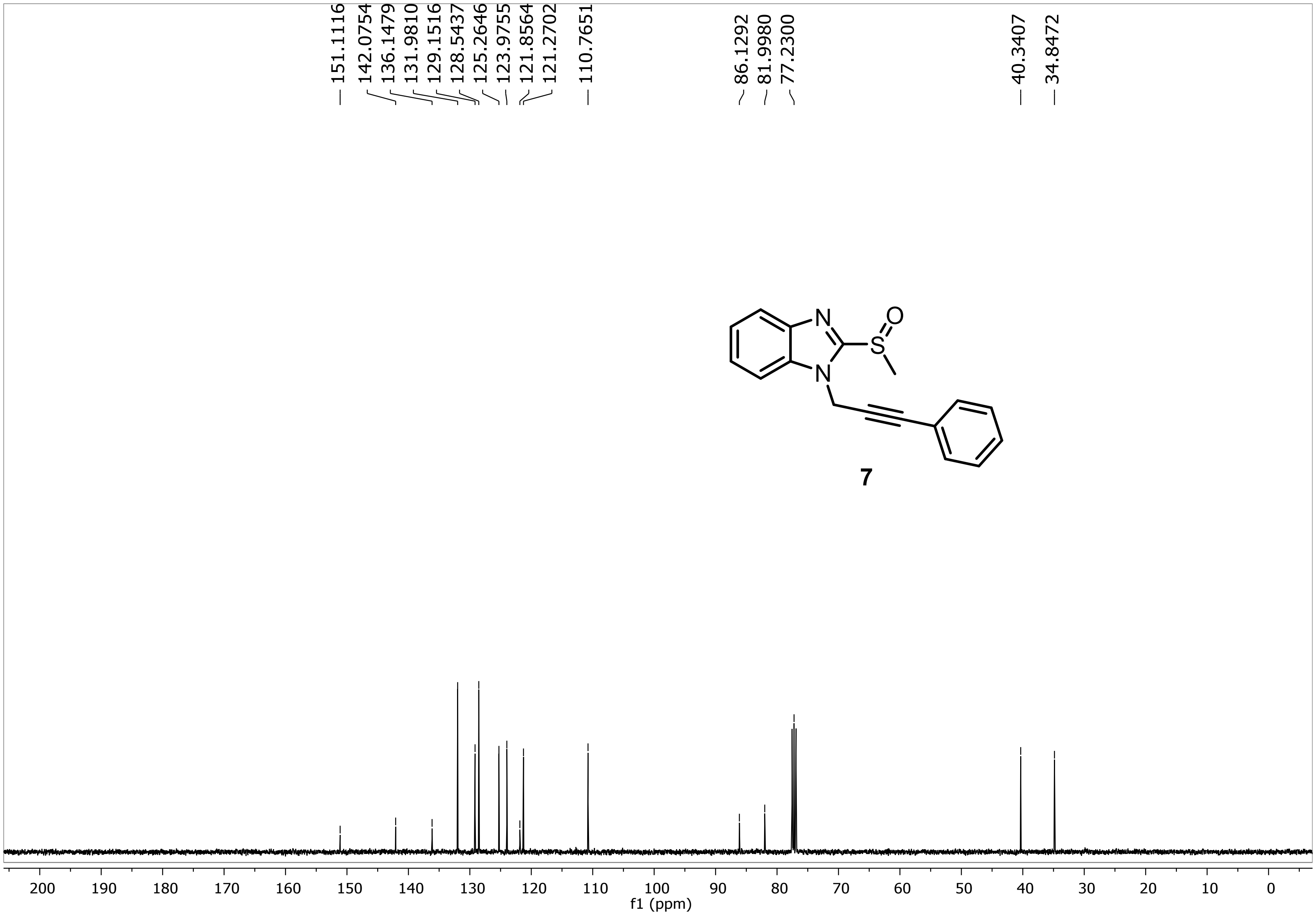

Figure S68: $^{13}C\{^{1}H\}$ NMR (100 MHz, $CDCl_3$) spectrum of compound **7**.

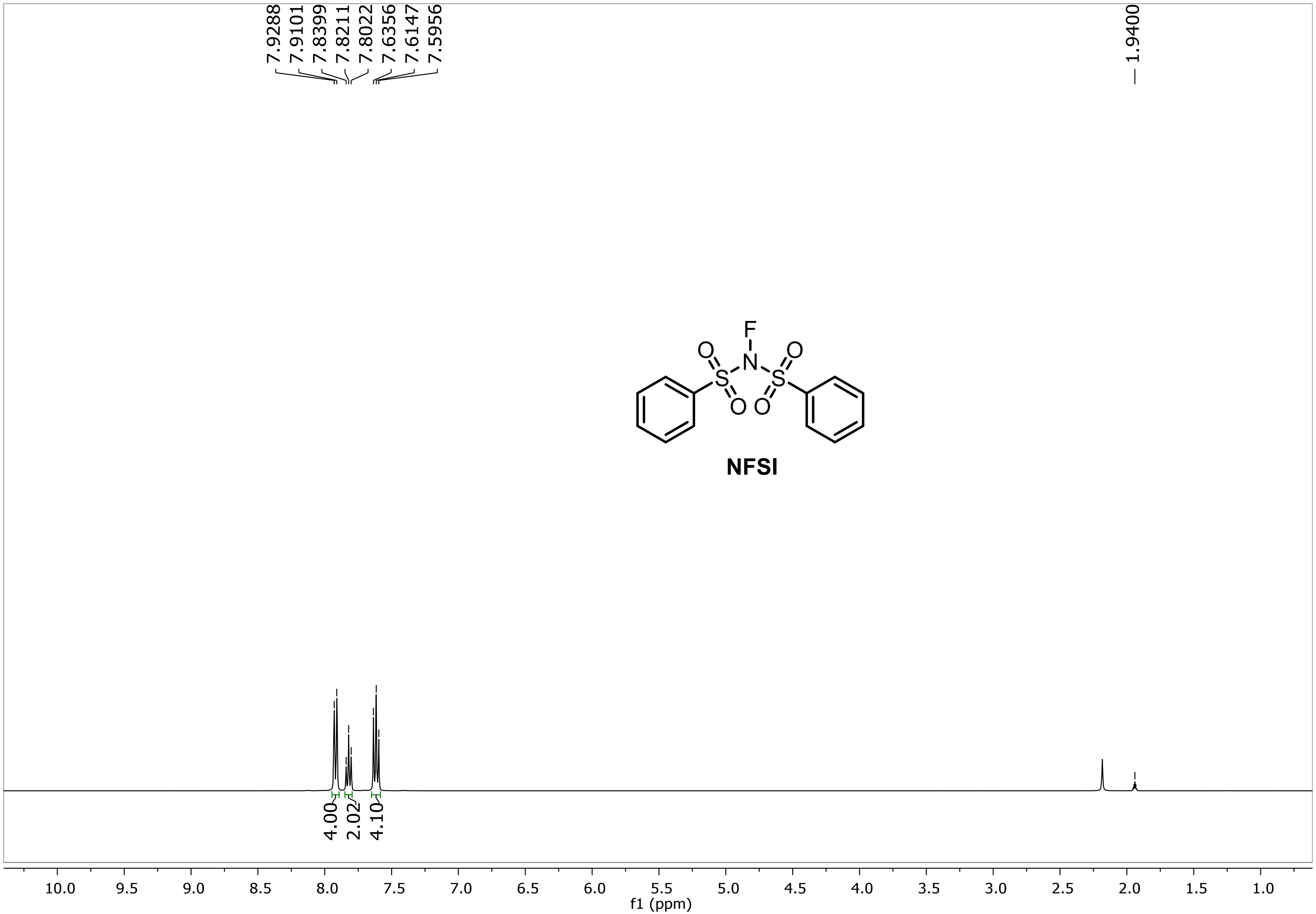

Figure S69: ^{1}H NMR (400 MHz, CD_3CN) spectrum of NFSI.

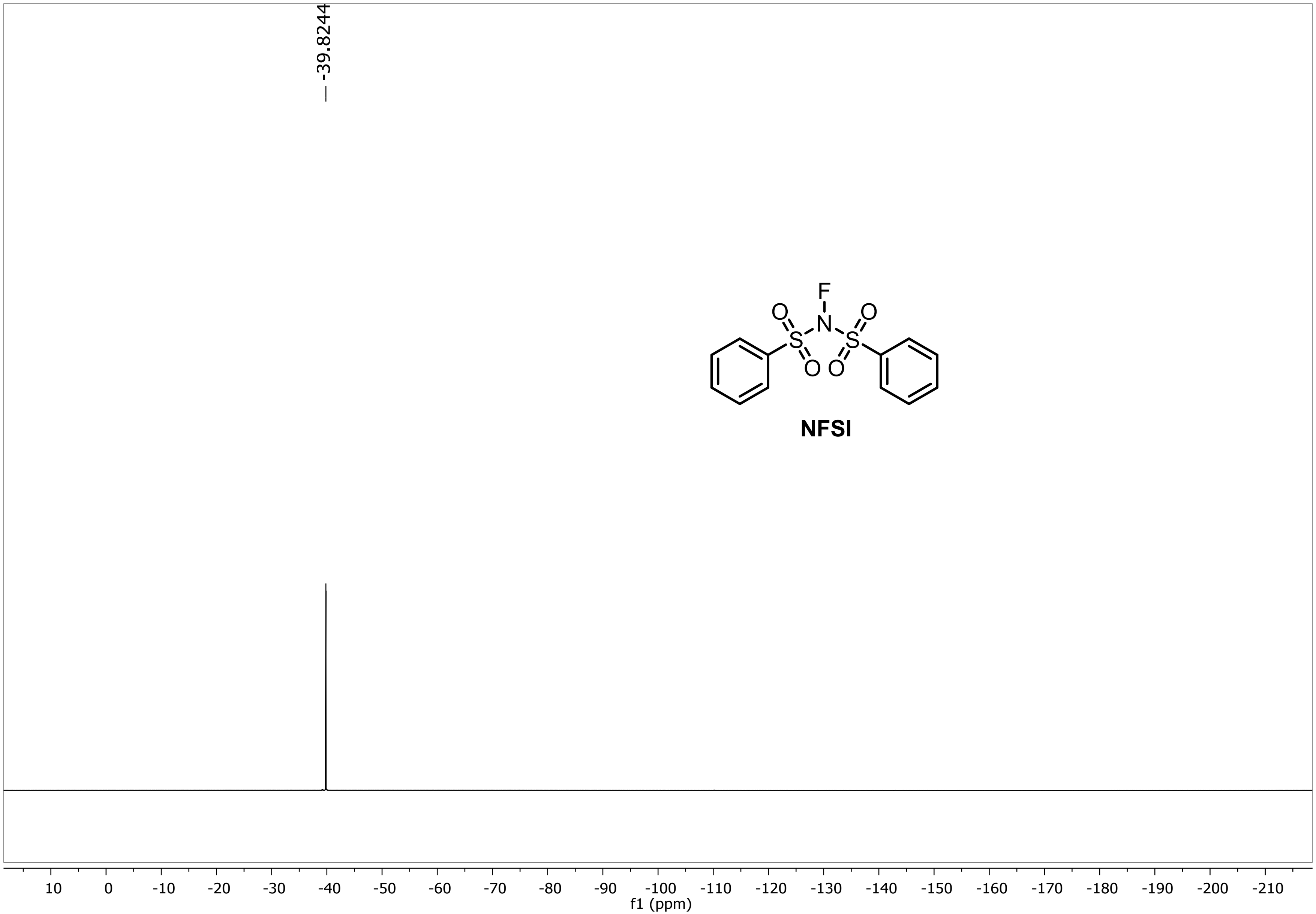

Figure S70: $^{19}F\{^{1}H\}$ NMR (376 MHz, CD_3CN) spectrum of NFSI.

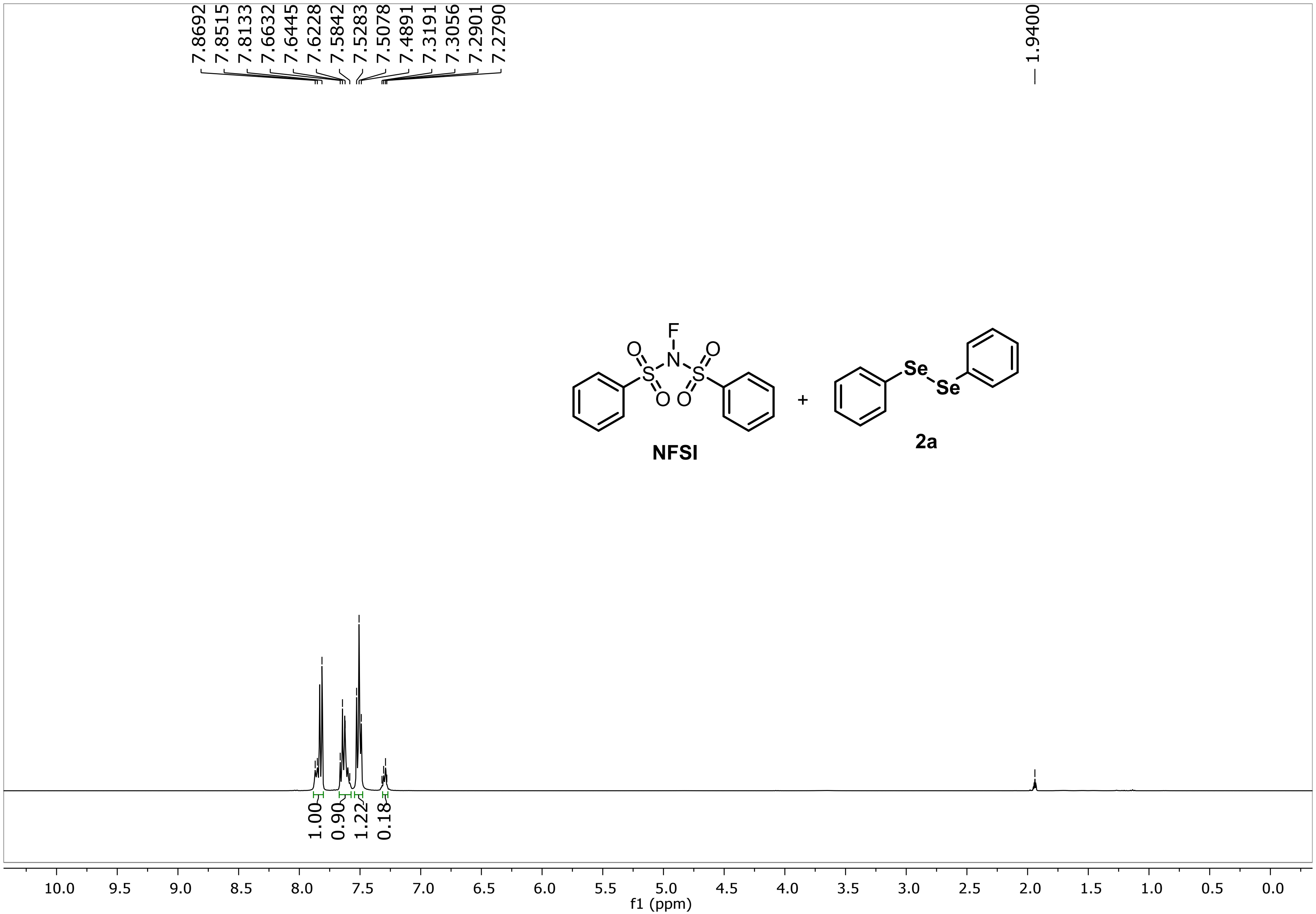

Figure S71: ^{1}H NMR (400 MHz, CD_3CN) spectrum of the mixture of NFSI + diphenyl diselenide **2a**.

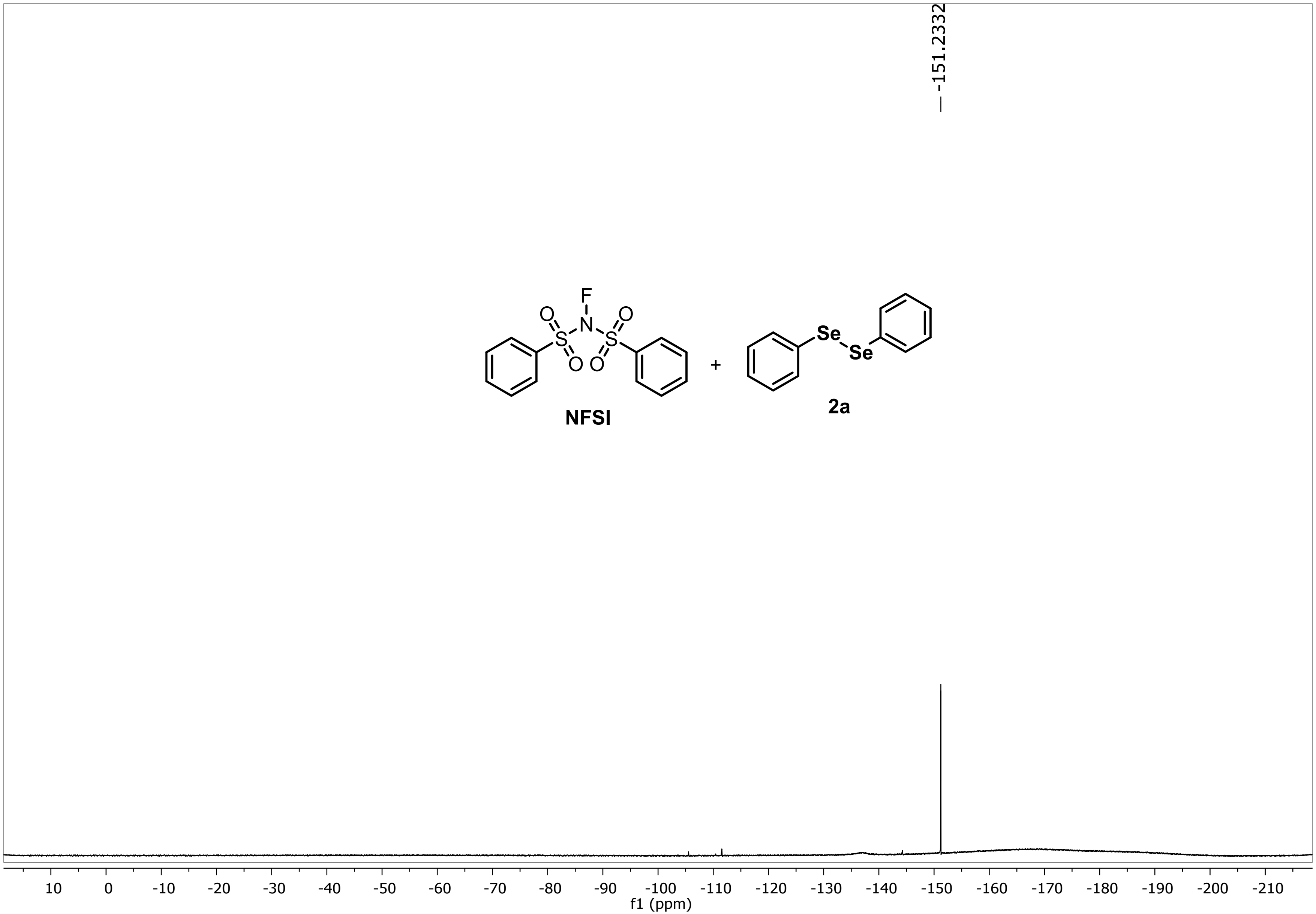

Figure S72: $^{19}F\{^{1}H\}$ NMR (376 MHz, CD_3CN) spectrum of the mixture of NFSI + diphenyl diselenide **2a**.

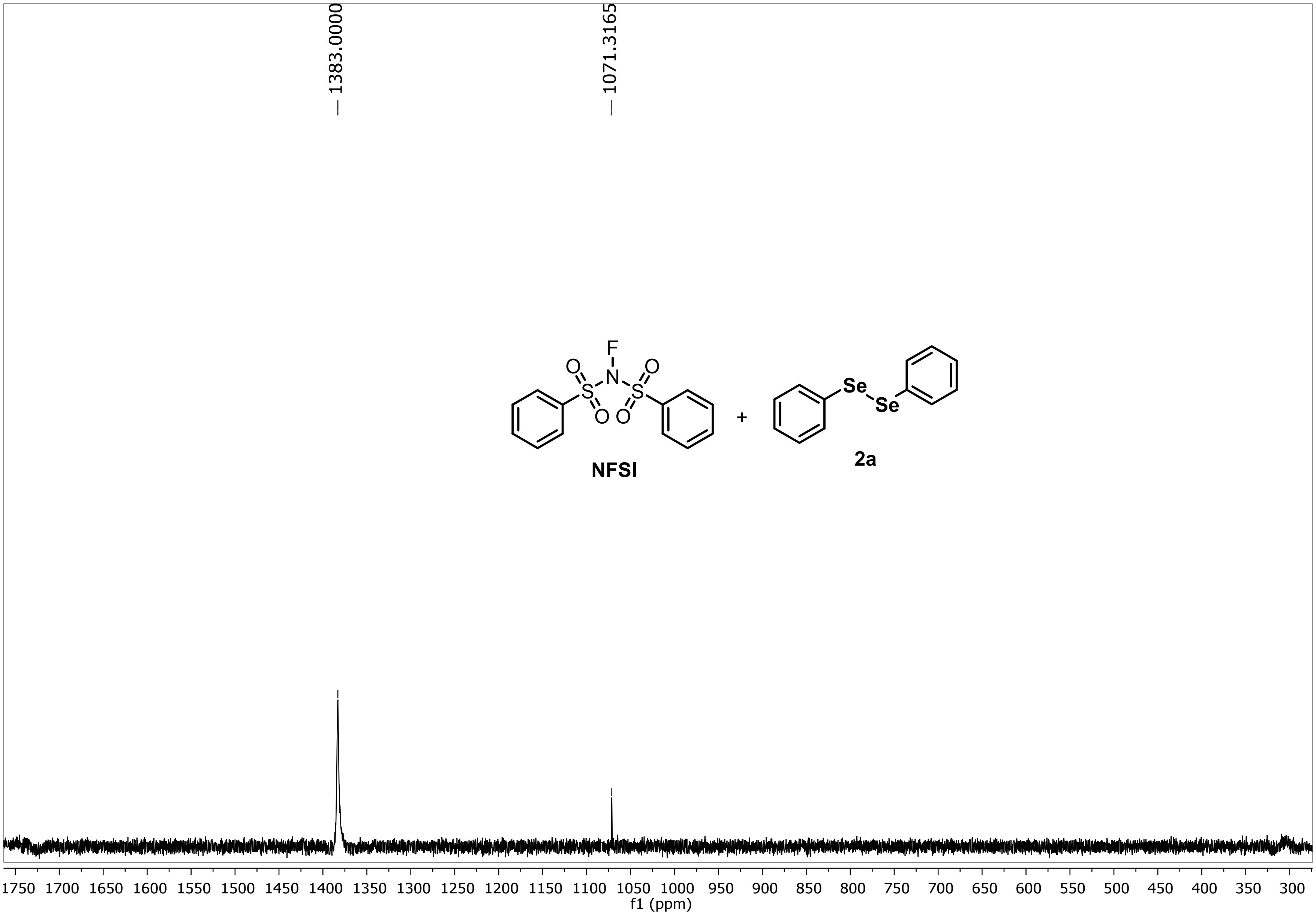

Figure S73: $^{77}Se\{^{1}H\}$ NMR (76 MHz, $CDCl_3$) spectrum of the mixture NFSI + diphenyl diselenide **2a**.

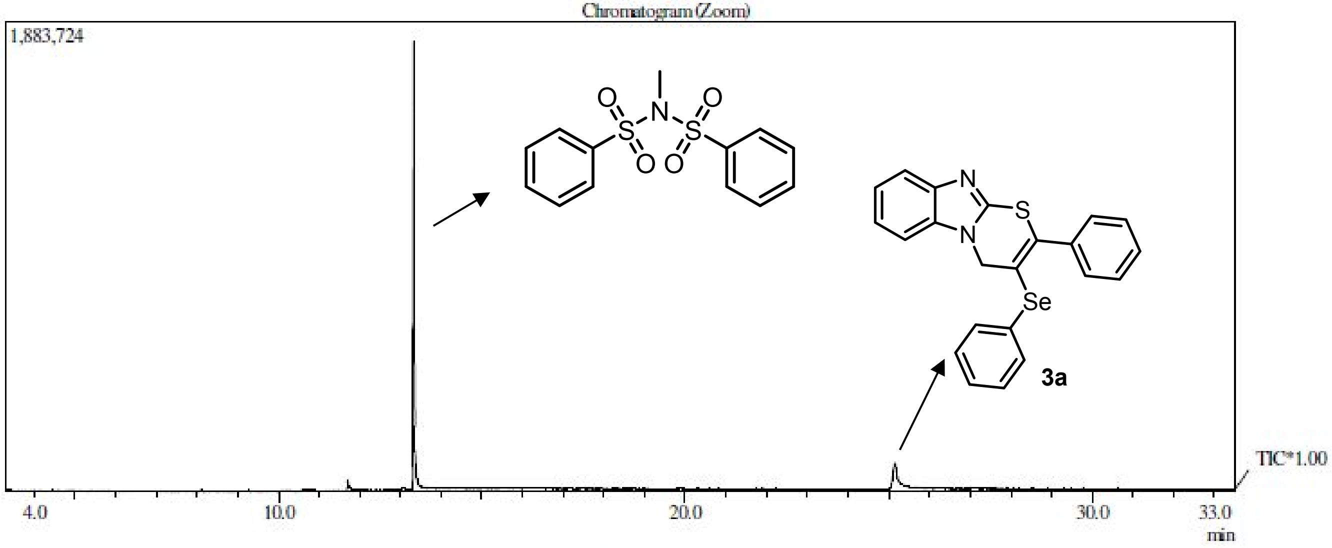

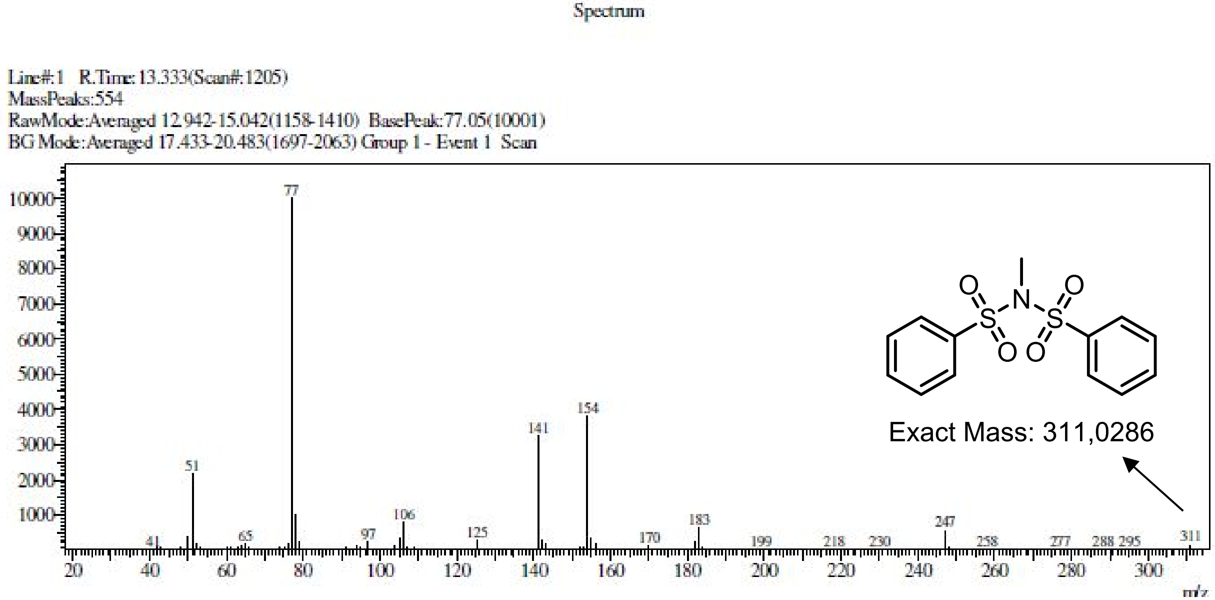

Figure S74: GC-MS of the reaction crude.

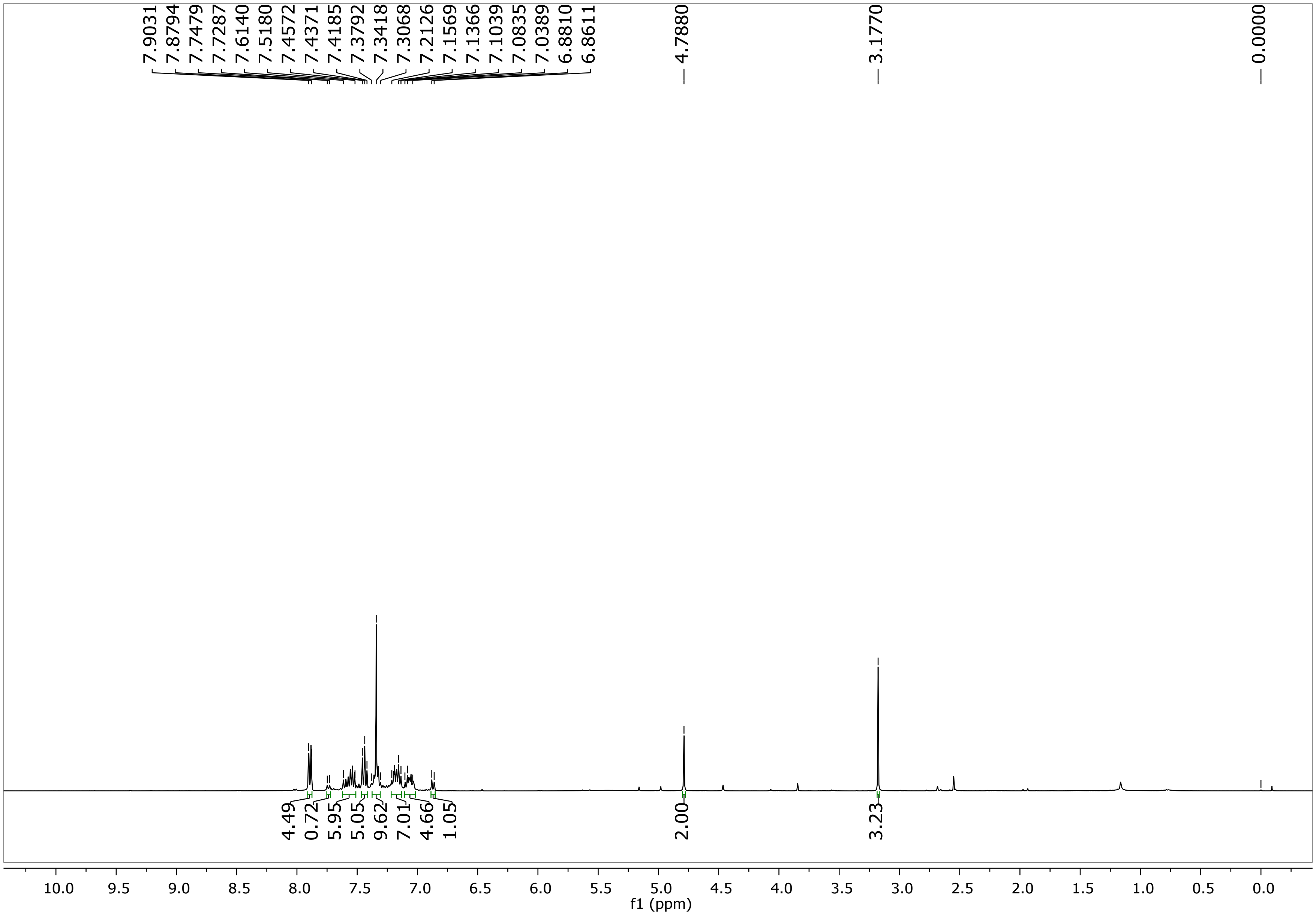

Figure S75: ^{1}H NMR (400 MHz, $CDCl_3$) spectrum of the reaction crude.
